# Whole genome sequencing reveals the genomic diversity, taxonomic classification, and evolutionary relationships of the genus *Nocardia*

**DOI:** 10.1371/journal.pntd.0009665

**Published:** 2021-08-26

**Authors:** Shuai Xu, Zhenpeng Li, Yuanming Huang, Lichao Han, Yanlin Che, Xuexin Hou, Dan Li, Shihong Fan, Zhenjun Li

**Affiliations:** 1 State Key Laboratory of Infectious Disease Prevention and Control, National Institute for Communicable Disease Control and Prevention, Chinese Center for Disease Control and Prevention, Beijing, China; 2 Key Laboratory of Medicine, Ministry of Education, School of Laboratory Medicine and Life Sciences, Wenzhou Medical University, Wenzhou, China; 3 School of Medical, Tibet University, Lhasa, Tibet, China; Lowell General Hospital, UNITED STATES

## Abstract

*Nocardia* is a complex and diverse genus of aerobic actinomycetes that cause complex clinical presentations, which are difficult to diagnose due to being misunderstood. To date, the genetic diversity, evolution, and taxonomic structure of the genus *Nocardia* are still unclear. In this study, we investigated the pan-genome of 86 *Nocardia* type strains to clarify their genetic diversity. Our study revealed an open pan-genome for *Nocardia* containing 265,836 gene families, with about 99.7% of the pan-genome being variable. Horizontal gene transfer appears to have been an important evolutionary driver of genetic diversity shaping the *Nocardia* genome and may have caused historical taxonomic confusion from other taxa (primarily *Rhodococcus*, *Skermania*, *Aldersonia*, and *Mycobacterium*). Based on single-copy gene families, we established a high-accuracy phylogenomic approach for *Nocardia* using 229 genome sequences. Furthermore, we found 28 potentially new species and reclassified 16 strains. Finally, by comparing the topology between a phylogenomic tree and 384 phylogenetic trees (from 384 single-copy genes from the core genome), we identified a novel locus for inferring the phylogeny of this genus. The *dapb1* gene, which encodes dipeptidyl aminopeptidase BI, was far superior to commonly used markers for *Nocardia* and yielded a topology almost identical to that of genome-based phylogeny. In conclusion, the present study provides insights into the genetic diversity, contributes a robust framework for the taxonomic classification, and elucidates the evolutionary relationships of *Nocardia*. This framework should facilitate the development of rapid tests for the species identification of highly variable species and has given new insight into the behavior of this genus.

## Author summary

*Nocardia* species can be responsible for opportunistic infections in humans, causing a variety of clinical presentations. They can also cause mycetoma in a normal host through direct inoculation. Although these species are often overlooked and misunderstood by modern medicine, they can cause life-threatening infections. However, most of our knowledge about *Nocardia* is based upon case reports and a few small studies of limited scope. This study provides an overview of the taxonomic and evolutionary structure of the genus *Nocardia* through extensive analysis of genome sequences. Our work aids the field by dissecting the genetic diversity of this species and improving the identification scheme for *Nocardia* species.

## Introduction

The genus *Nocardia*, first described in 1888 by Edmund Nocard, belongs to the family Nocardiaceae of the order Corynebacteriales in the phylum *Actinobacteria* [1]. The members of this genus are Gram-positive, aerobic, non-motile, and acid-fast actinomycetes. At the time of writing, there were 115 recognized species with valid names in LPSN, the List of Prokaryotic names with Standing in Nomenclature (https://lpsn.dsmz.de/genus/nocardia). Of these described species, many have been implicated to be the cause of human infections, especially in immunocompromised patients [2,3]. These infections range from cutaneous and subcutaneous diseases, to necrotizing pneumonia and even brain abscess [4,5].

Pulmonary and central nervous system diseases have been reported particularly in patients with debilitating underlying conditions, such as AIDS, organ transplants, or diabetes [6,7]. Cutaneous and subcutaneous diseases were caused by traumatic inoculation of the organism in a normal host [8]. The classical infection is the mycetoma, and this is currently listed as a neglected tropical disease by the World Health Organization (WHO). Although mycetoma is usually found in the foot, this chronic infection can spread to the muscles, lungs, and spinal cord, and may cause disability or even mortality [9–12].

Accurate taxonomy can improve any understanding of the evolution, epidemiology, and pathogenicity of bacteria. However, the phylogenetic position and genetic diversity of *Nocardia* are not yet fully understood [2,13]. Phylogenetic analysis based only on the 16S rRNA gene as a molecular marker has led to misclassifications when comparing closely related species due to their high sequence similarities [8,14]. To overcome the limitations of the 16S rRNA gene, other single loci, such as *secA1*, *sodA*, *gyrB*, *hsp65*, or *ropB*, have been analyzed [15–20]. However, the discriminatory power of these loci is not sufficiently accurate in differentiating between clinically relevant species. A multilocus sequence analysis (MLSA) that relies on multiple conserved molecular markers (*secA1*, *rpoB*, *gyrB*, and *hsp65*, along with 16S rRNA) was shown to be more reliable for species discrimination compared with single-gene sequence-based phylogeny [21,22]. However, the identification of uncommon species remains a challenge regardless of the method used.

Whole-genome sequencing (WGS) has been effectively used in the phylogenetic and taxonomic analyses of several bacterial taxa [23–27]. However, information on what delineates members of the genus *Nocardia* is rare. In this study, we established a phylogenomic approach to provide insight into the diversity and evolution of the genus *Nocardia* and formed a framework for taxonomic classification of this species, highlighting 28 potential novel species and several cases of misidentification, as well as identifying a well-suited candidate gene for species identification in this genus.

## Results and discussion

### Genomic features of the genus *Nocardia*

Our genome-scale study used the most complete sampling of the diversity of *Nocardia* species published to date. The strains investigated were originally isolated from a wide spectrum of environmental conditions and human, animal, or plant hosts. Among these strains, approximately half were recognized as human and/or animal pathogens. Genomic features of these strains, including the G+C content, genome size, and number of CDSs, are presented in S1 Table and S1 Fig. Briefly, the G+C content of the genomes of these strains ranged from 65.5% to 72.0%, with an average of 68.42%. These genomes varied in size by approximately 7.61 Mb (range from 5.04 to 10.52 Mb), with coding sequence (CDS) numbers ranging from 4626 to 9617, suggesting substantial genomic diversity of this genus.

### Pangenome construction of type strains

**Fig 1:**
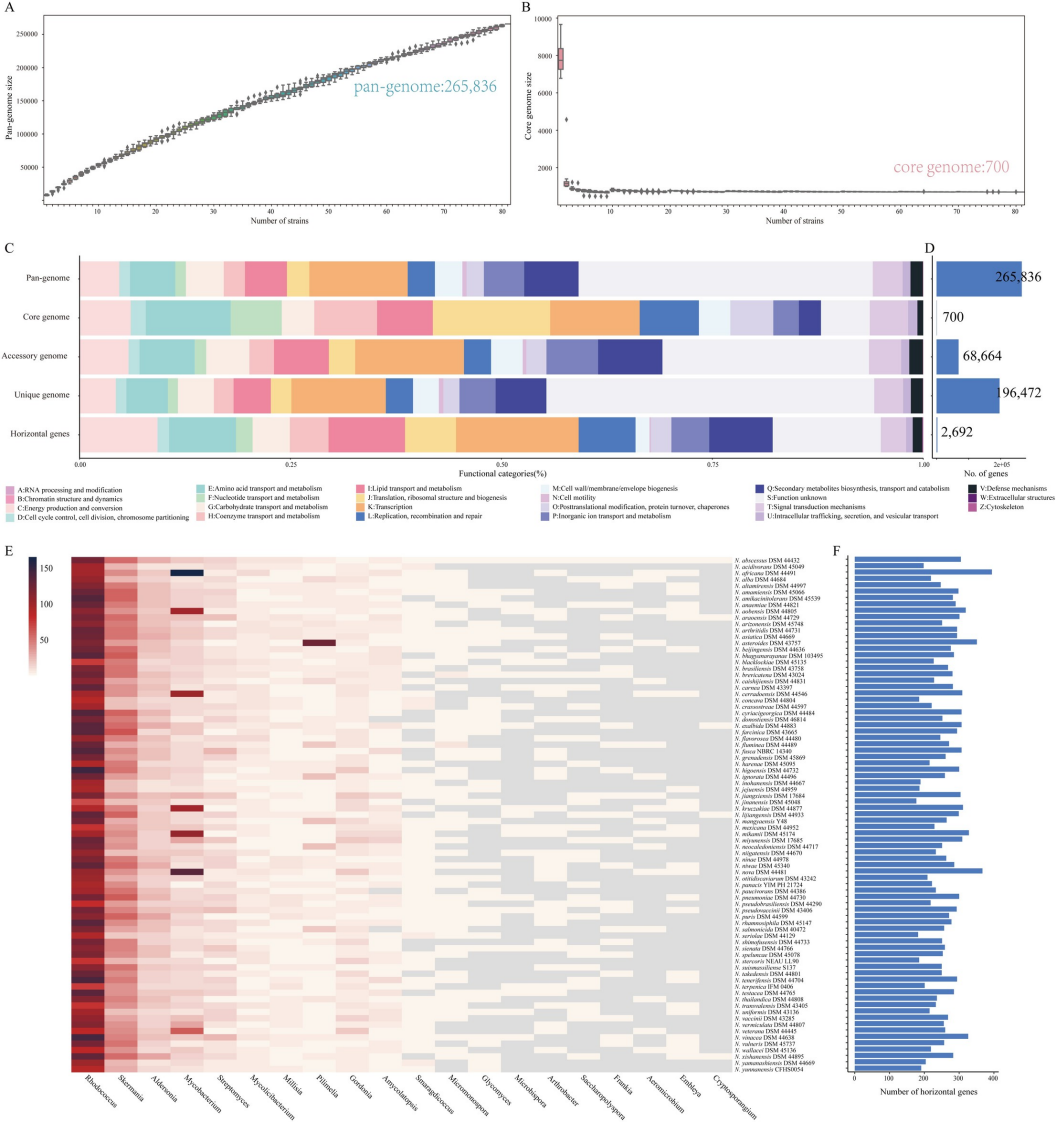
Pan-genome structure and function of type strains of *Nocardia*. (A) Gene accumulation curves for the pan-genome. (B) Gene accumulation curves for the core genome. The cumulative sizes of the pan-genome and core genome were calculated by type strains without replacement in random order 1000 times and then used to calculate the mean size. Error bars indicate one standard deviation from the mean. Five synonymous species were removed as described in Fig 2 and Table 1. (C) Distribution of functional categories in *Nocardia* core, accessory, and unique genomes and horizontal genes. (D) The number of gene families in each gene set. (E) The 20 potential donor bacterial genera providing donor genes for HGT. (F) Distribution of horizontal genes acquired in *Nocardia* spp.

To characterize the genomic composition of the genus *Nocardia*, genomes from 86 type strains were used for pan-genome analysis. Based on the gene accumulation curve, *Nocardia* exhibited an open-genome structure whose size increased continuously with the number of added genomes and contained 265,836 gene families (Fig 1A). Of these gene families, only 700 (0.26%) were identified as core genes and 68,664 (25.8%) were identified as accessory genes. The remaining 196,472 (73.9%) gene families were specific to a single strain that constituted unique genomes, suggesting a high degree of genetic variation (Fig 1D). The number of unique genes in *Nocardia* was diverse, ranging from 324 to 6387 (S2B Fig). Remarkably, *N*. *stercoris* NEAU LL90^T^ contained 5229 unique genes, accounting for 78% of its genome content. The considerable number of unique genes further reflected the heterogeneity of this genus, implying it has very high genome plasticity.

### Functional genome analyses

To gain insight into the functional features of the *Nocardia* pan-genome, we characterized the functions of the core, accessory, and unique genes by mapping them to the eggNOG database. A high proportion (34.9%) of the pan-genome was poorly characterized as “S: function unknown” since the proteins encoded by these genes were either functionally unknown or did not have homologs outside of this genus. The core genome was enriched in genes involved in the maintenance of primary cellular process, including information storage and processing (“J: translation, ribosomal structure, and biogenesis” [101 genes], “K: transcription” [77genes]) and metabolism (“E: amino acid transport and metabolism” [73 genes], and “H: coenzyme transport and metabolism” [54 genes]) (Fig 1C). We also evaluated the functional categories of all groups of core genomes. The relative distribution of these functional categories was similar (S3A Fig).

Additionally, high proportions of accessory genes (24.5%) and unique genes (39.9%) were poorly characterized. Genes assigned to “K: transcription” (9808 genes), “Q: secondary metabolite biosynthesis, transport, and catabolism” (5802 genes), “E: amino acid transport and metabolism” (4966 genes), “I: lipid transport and metabolism” (4948 genes), and “P: inorganic ion transport and metabolism” (4658 genes) were prominently represented in the accessory component of this pan-genome. Unique genes were prominently enriched in “K: transcription” (22,809 genes), “Q: secondary metabolite biosynthesis, transport, and catabolism” (12,251 genes), “E: amino acid transport and metabolism” (10,006 genes), “I: lipid transport and metabolism” (8984 genes), and “P: Inorganic ion transport and metabolism” (8729 genes). Moreover, the proportion of the unique genome assigned to “V: defense mechanisms” (1.42%) was higher than that in the core genome (0.69%). Strains such as *N*. *pseudobrasiliensis* DSM 44290^T^, *N*. *stercoris* NEAU LL90^T^, and *N*. *uniformis* DSM 43136^T^ possessed more unique genes involved in “secondary metabolite biosynthesis, transport, and catabolism” (S2A Fig), indicating their high metabolic capacities. Indeed, *Nocardia* species are known to have the ability to produce a wide variety of secondary metabolites with biological activity. Many members of this genus exhibit unique capacities, producing biological activities, such as antimicrobial, antitumor, antioxidative, and immunosuppressive activities, and metabolizing aliphatic and aromatic toxic hydrocarbons, natural or synthetic polymers, and other widespread environmental pollutants [28,29]. The tremendous metabolic diversity of *Nocardia* spp. highlights their potential and would need to be investigated in future work.

### Potential HGT in *Nocardia*

HGT is the main driver of bacterial evolution and diversity and is crucial for rapid adaptation to changing environmental conditions [30]. Thus, we examined all horizontally acquired genes and tracked their potential donor taxa. Gene transfer occurred in 2692 gene families, 980 of which were unique genes, which indicated that HGT contributed to the open pan-genome of *Nocardia* (Fig 1D). These horizontal genes were mainly involved in “K: transcription” (431 genes, 16.0%), “C: energy production and conversion” (273, 10.1%), “I: lipid transport and metabolism” (269, 10.0%), “E: amino acid transport and metabolism” (235, 11.7%), and “Q: Secondary metabolite biosynthesis, transport, and catabolism” (223, 8.3%) (Fig 1C). In addition, a total of 154 potential donor taxa were identified. *Rhodococcus*, *Skermania*, *Aldersonia*, and *Mycobacterium* appeared to be the leading donor taxa, indicating that *Nocardia* shares some properties with these genera (Fig 1E).

HGT also contributed to the core genome of *Nocardia*. A total of 107 core gene families appeared to potentially have been acquired via HGT (S2 Table), mainly from the genera *Rhodococcus*, *Skermania*, *Aldersonia*, and *Mycobacterium*, suggesting similarity in the evolution of these genera. This may have been a cause of the confusion concerning *Nocardia* taxonomy, historically speaking [31,32].

### Phylogenomic analysis of type strains

**Fig 2:**
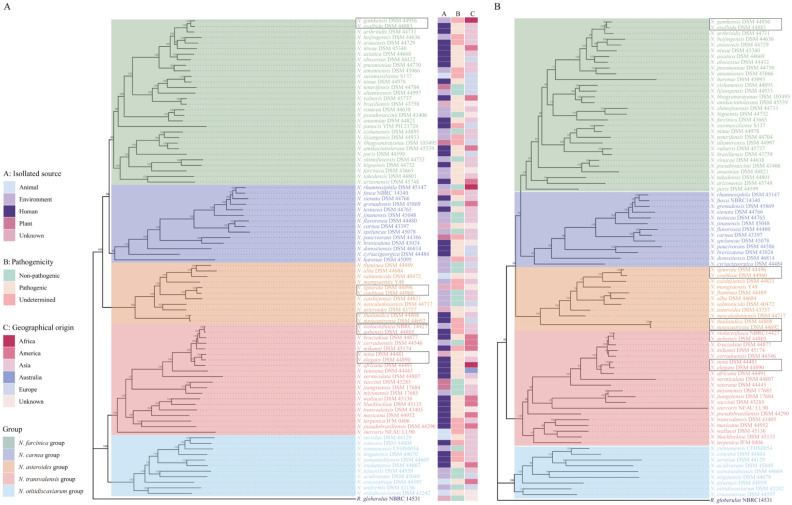
Phylogenomic and phylogenetic trees of the type strains of *Nocardia*. (A) A phylogenomic tree was constructed based on the concatenation of 384 single-copy genes from 86 type strains. The isolated source, pathogenicity, and geographical origin are shown. (B) Phylogenetic tree based on the single gene *dapb1* from 86 type strains. Each tree was constructed by the maximum likelihood method with 1000 bootstrap replicates. *Rhodococcus globerulus* NBRC 14531 served as an outgroup. Bootstrap values are indicated on the nodes. The colored branches indicate the five main phylogenetic groups. Black boxes indicate synonymous species.

To elucidate the taxonomic relationship between members of the genus *Nocardia*, we constructed a high-quality maximum-likelihood phylogenomic tree based on the concatenation of 384 conserved single-copy genes (Fig 2A and S3 Table). The phylogenomic tree revealed five main phylogroups, composed of 11 to 30 species, with robust bootstrap support. The reconstructed genome phylogeny showed independent branches between species, except for six sets of type strains, including *N*. *gamkensis–N*. *exalbida*; *N*. *vulneris–N*. *brasiliensis*; *N*. *ignorata–N*. *coubleae*; *N*. *thailandica–N*. *novocastrense*; *N*. *violaceofusca–N*. *aobensis–N*. *kruczakiae*, and *N*. *nova–N*. *elegans*, indicating their close relationship with one another.

### Synonymous species of *Nocardia*

**Fig 3:**
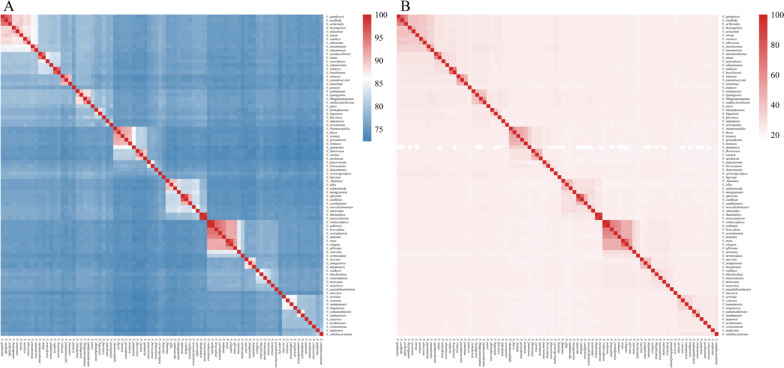
Heatmap of pairwise ANI and *is*DDH values for 86 type strains. (A) Species delineation at an ANI threshold of 96% and (B) *is*DDH threshold of 70%. The isolate names on the left are the same as those in Fig 2A with the outgroup removed. The values underlying this heatmap were provided in the S4 and S5 Tables.

The average nucleotide identity (ANI) and *in silico* DNA-DNA hybridization (*is*DDH) values of the 86 type strains of the genus *Nocardia* were used to assess the overall genome similarity (Fig 3 and S4 and S5 Tables). The current standards for a strain to be considered as belonging to the same species are: ≥ 95%–96% of ANI or ≥ 70% of *is*DDH [33,34]. If we choose a threshold of 95% ANI, the producing results could not meet the *is*DDH boundary of 70%. Given the consistency of ANI and *is*DDH, we chose 96% as our ANI cutoff. This value was conservative, but it avoided the divergence increases or inappropriate changes.

The examined members of the *Nocardia* genus were found to have ANI values higher than 72.33% or *is*DDH values higher than 13.9%. Five sets of synonymous species were identified based on the cut-off values for species delineation using ANI (96%) and *is*DDH (70%) (Fig 2 and Table 1).

**Table 1:** Inconsistent species assignment of strains in the genus *Nocardia*.

Phylogroup	Strain	Closest related type strain	ANI (%)	*is*DDH (%)	Species
*N*. *farcinica* group	*N*. *gamkensis* DSM 44956^T^	*N*. *exalbida* DSM 44883^T^	96.65	73.7	*N*. *exalbida*
*N*. *asteroides* group	*N*. *coubleae* DSM 44960^T^	*N*. *ignorata* DSM 44496^T^	96.8	74.8	*N*. *ignorata*
*N*. *asteroides* group	*N*. *novocastrense* DSM 44692^T^	*N*. *thailandica* DSM 44808^T^	98.68	89	*N*. *thailandica*
*N*. *transvalensis* group	*N*. *violaceofusca* NBRC 14427^T^	*N*. *aobensis* DSM 44805^T^	98.95	93.8	*N*. *aobensis*
*N*. *transvalensis* group	*N*. *elegans* DSM 44890^T^	*N*. *nova* DSM 44481^T^	96.72	75.1	*N*. *nova*
*N*. *farcinica* group	*N*. *brasiliensis* DSM 46032	*N*. *vulneris* DSM 45737^T^	98.58	87.9	*N*. *vulneris*
*N*. *transvalensis* group	*N*. *nova* DSM 40806	*N*. *aobensis* DSM44805^T^	96.63	74.5	*N*. *aobensis*

Notably, two minor inconsistencies were observed when the phylogenomic tree and whole-genome comparisons were compared. *N*. *vulneris* DSM 45737^T^ possessed a high genome sequence identity with *N*. *brasiliensis* DSM 43758^T^, with an ANI of 95.64% and *is*DDH of 65.6%, but had not reached the species boundary. Similarly, although *N*. *kruczakiae* DSM 44877 ^T^ was highly similar to the genome sequences of *N*. *aobensis* DSM 44805 ^T^, the observed ANI (95.49%) and *is*DDH values (65.3%) between them would not support their classification as the same species. This could be explained by the fact that our phylogenomic tree is based on single-copy core genes, and these genes can be expected to evolve slowly. In contrast, ANI and *is*DDH are based on pairwise whole-genome comparisons and thus also encompass recently gained, potentially fast-evolving genes. Additionally, it is worth mentioning that our analyses used draft genomes with a varying number of scaffolds (ranging from 2 to 492), making species designation by ANI less accurate. Considering the high diversity and high degree of HGT in *Nocardia*, our phylogenomic tree might be a better reflection of evolutionary distance. To provide a more precise answer, it will require sequencing the complete genome of strains that are taxonomically controversial for future analyses.

### Genomic diversity of *Nocardia* spp

Phylogenomic inferences coupled with whole-genome comparisons were performed to further evaluate intraspecies relationships and the genomic diversity of an additional 122 strains, including 27 reference strains and 42 clinical isolates obtained in this work. Species clusters were binned as follows: (i) genomic clusters with one type strain genome with an ANI value ≥ 96%; (ii) genomic cluster without a type strain, and/or an ANI value < 96% to the type strain; or (iii) individual strains that area type strain or formed independent branch. In total, 99 distinct species clusters were identified, of which 17 contained a single type strain from exactly one species, with an ANI value higher than 96.03%, thus allowing the taxonomic assignment of these strains (S4 Fig).

Despite some genomes being clustered with their expected type strain, many formed several independent branches inside, which were subgroups. For example, the genomes belonging to the species *N*. *abscessus*, *N*. *brasiliensis*, *N*. *cyriacigeorgica*, *N*. *carnea*, *N*. *nova*, *N*. *transvalensis*, *N*. *pseudobrasiliensis*, and *N*. *otitidiscaviarum* had a type strain in different subgroups. Further ANI analyses revealed that these species clusters contained at least one member with inconsistent species classification except for *N*. *otitidiscaviarum*.

Our intraspecies analysis highlighted two species clusters (*N*. *transvalensis* and *N*. *pseudobrasiliensis*) with two members representing different species. For example, although *N*. *transvalensis* DSM 46068 was closely related to *N*. *transvalensis* DSM 43405^T^, comparisons between the two strains resulted in an ANI value of 90.99%.

Moreover, five species clusters contained two or more subgroups. The *N*. *abscessus* cluster had two subgroups. The genome sequences of one reference strain (DSM 44557) and one clinical strain (CDC 167) generated ANI values lower than 95.34% compared to *N*. *abscessus* DSM 44432^T^, suggesting that they were distinct species. The *N*. *nova* cluster had two strains (MDA3139 and MDA0897) displaying an ANI of < 93.6% compared to *N*. *nova* DSM44481^T^ and thus were considered to be separate species. Moreover, one genome sequence was mislabeled as an *N*. *nova* species (strain DSM 40806) in the *N*. *aobensis* cluster (Table 1).

The members of the species *N*. *cyriacigeorgica* were observed to have high genomic diversity. The genomes belonging to subgroup 2 (clinical strain CDC 327, CDC 182), subgroup 3 (EML446, EML1456), and subgroup 4 (DSM 43005, DSM 43004, DSM 46058, DSM40350, CDC 332, and GUH-2) produced ANI values that were below 92% compared to *N*. *cyriacigeorgica* DSM44484^T^ and should be considered separate species rather than subspecies, indicating that 50% of the currently named *N*. *cyriacigeorgica* genomes should be taxonomically revised.

A similar situation occurred for the *N*. *brasiliensis* cluster, which included five subgroups, representing five putative distinct species. Notably, *N*. *brasiliensis* DSM 46032 was shown to be more similar to *N*. *vulneris* DSM 45737^T^ than the representative species *N*. *brasiliensis* DSM43758^T^, implying this species had a high probability of being misclassified (Table 1).

The remaining seven clusters had no type strains, potentially representing novel species of *Nocardia* (Table 2). These species include five new clinical isolates from patients in China (CDC 188, CDC 186, CDC 159/CDC 141, CDC 160, and CDC 153) and two new species that were wrongly assigned to *N*. *asteroids* (strain DSM 43258) and *N*. *nova* (strain SH22a).

**Table 2:** General features of novel species.

	Phylogroup	Strain	Closest related type strain	ANI (%)	*is*DDH (%)
1	*N*. *farcinica* group	*N*. *brasiliensis* IFM 10847, CDC 144, CDC 163, CDC 196	*N*. *brasiliensis* DSM 43758^T^	95.15	61.7
2	*N*. *farcinica* group	*N*. *brasiliensis* DSM 46059	*N*. *brasiliensis* DSM 43758^T^	94.95	61.9
3	*N*. *farcinica* group	*N*. *brasiliensis* HUJEG 1	*N*. *brasiliensis* DSM 43758^T^	94.84	60.3
4	*N*. *farcinica* group	*N*. *abscessus* DSM 44557, CDC 167	*N*. *abscessus* DSM 44432^T^	95.37	66
5	*N*. *farcinica* group	CDC 188	*N*. *abscessus* DSM 44432^T^	89.42	38.3
6	*N*. *farcinica* group	CDC 186	*N*. *beijingensis* DSM 44636^T^	94.36	56.8
7	*N*. *farcinica* group	*Nocardia* sp. SYSU K10002	*N*. *takedensis* DSM 44801^T^	80.25	23.3
8	*N*. *farcinica* group	*Nocardia* sp. CNY 236	*N*. *amamiensis* DSM 45066^T^	80.48	24.2
9	*N*. *farcinica* group	*Nocardia* sp. CICC 11023	*N*. *tenerifensis* DSM 44704^T^	86.45	31.8
10	*N*. *carnea* group	CDC 182, CDC 327	*N*. *cyriacigeorgica* DSM 44484^T^	91.02	43.5
11	*N*. *carnea*group	EML1456, EML446	*N*. *cyriacigeorgica* DSM 44484^T^	91.99	47.1
12	*N*. *carnea* group	*N*. *cyriacigeorgica* GUH-2, CDC 332, *N*. *cyriacigeorgica* DSM40350, *N*. *cyriacigeorgica* DSM46058, *N*. *cyriacigeorgica* DSM43004, *N*. *cyriacigeorgica* DSM43005	*N*. *cyriacigeorgica* DSM 44484^T^	90.34	47.1
13	*N*. *carnea* group	*N*. *carnea* DSM 46071, *N*. *carnea* DSM 44558, *N*. *carnea* DSM 44582	*N*. *carnea* DSM 43397^T^	94.87	64.5
14	*N*. *asteroides* group	*N*. *asteroides* DSM 43258	*N*. *asteroids* DSM 43757^T^	87.73	33.7
15	*N*. *transvalensis* group	*N*. *nova* MDA0897, *N*. *nova* MDA 3139	*N*. *nova* DSM 44481^T^	93.55	55.9
16	*N*. *transvalensis* group	*N*. *nova* SH22a	*N*. *vermiculata* DSM 44807^T^	81.3	24.5
17	*N*. *transvalensis* group	*N*. *pseudobrasiliensis* IFM 0761	*N*. *pseudobrasiliensis* DSM 44291^T^	94.08	57.9
18	*N*. *transvalensis* group	CDC 141, CDC 159	*N*. *pseudobrasiliensis* DSM 44290^T^	84.71	28.9
19	*N*. *transvalensis* group	*N*. *transvalensis* DSM 46068	*N*. *transvalensis* DSM 43405^T^	90.99	43.2
20	*N*. *transvalensis* group	*Nocardia* sp. BMG111209	*N*. *stercoris* NEAU LL90^T^	77.02	21.3
21	*N*. *transvalensis* group	*Nocardia* sp. RB56	*N*. *stercoris* NEAU LL90^T^	77.11	21.5
22	*N*. *transvalensis* group	*Nocardia* sp. BMG51109	*N*. *blacklockiae* DSM 45135^T^	83.56	27.7
23	*N*. *transvalensis* group	*Nocardia* sp. RB20	*N*. *vaccinii* DSM 43285^T^	86.53	32.4
24	*N*. *otitidiscaviarum* group	CDC 160	*N*. *concava* DSM 44804^T^	86.36	31.5
25	*N*. *otitidiscaviarum* group	CDC 153	*N*. *concava* DSM 44804^T^	86.43	31.7
26	*N*. *otitidiscaviarum* group	*Nocardia* sp. SYP-A9097	*N*. *acidivorans* DSM 45049^T^	87.76	34.7
27	*N*. *otitidiscaviarum* group	*Nocardia* sp. CT2-14	*N*. *niigatensis* DSM 44670^T^	87.98	35.2
28	*N*. *otitidiscaviarum* group	*Nocardia* sp. ET3-3	*N*. *concava* DSM 44804^T^	86.55	32.3

Overall, the phylogenomic taxonomy of the 203 available genomes of *Nocardia* revealed the putative existence of 18 novel species and several inconsistencies with the traditional classification of strains into species. To obtain more insight into the species classification of *Nocardia*, the physiological and biochemical characteristics, as well as the antimicrobial profiles of these potentially new species should be investigated further in the future.

### Genome reclassifications

**Fig 4:**
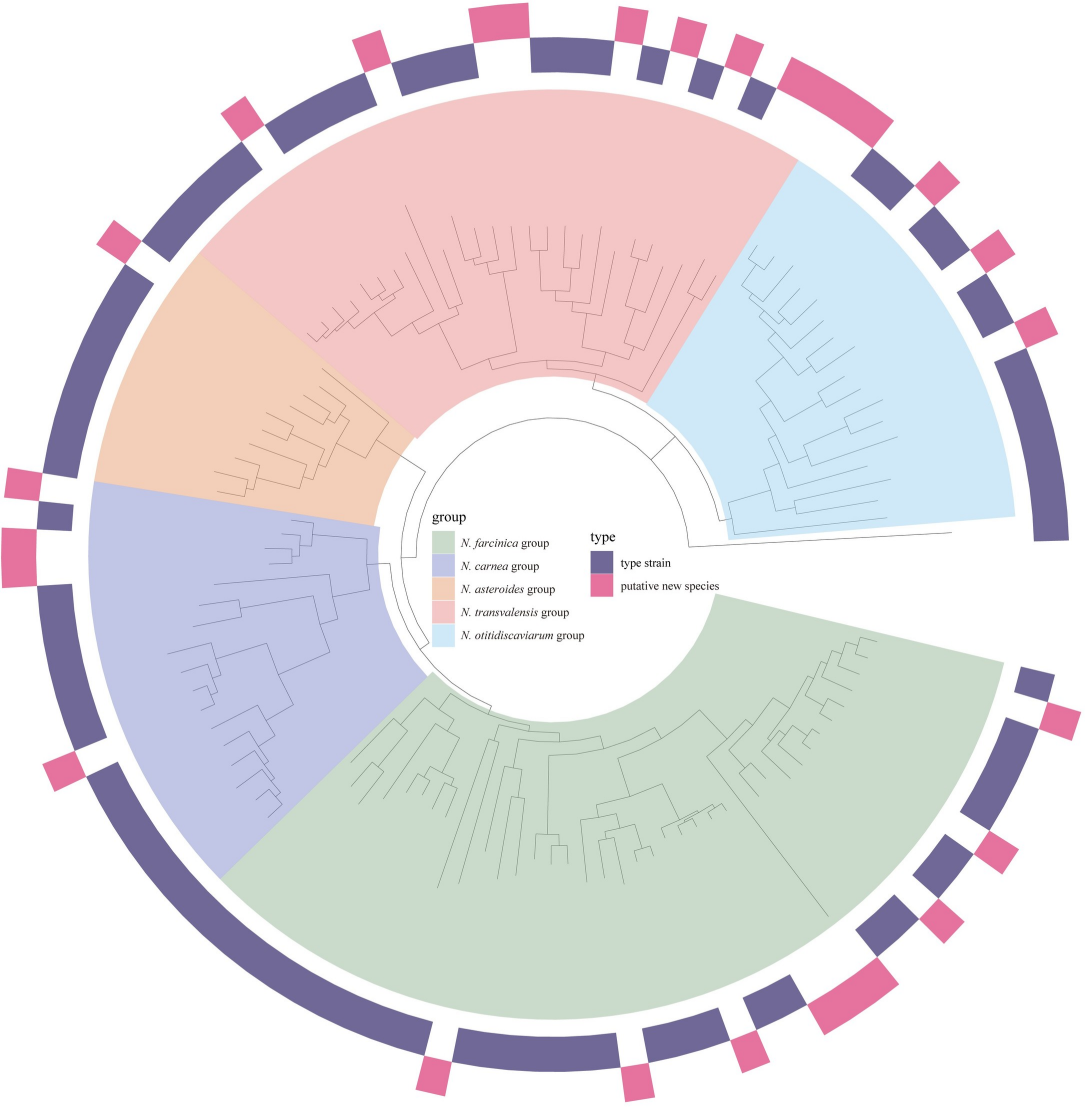
Phylogenomic tree of members of the genus *Nocardia*. A maximum-likelihood phylogeny tree was built based on the concatenation of 298 single-copy genes from 81 type strains and 28 putative new species with 1000 bootstrap replicates. The genome of *Rhodococcus globerulus* NBRC 14531 was included as an outgroup. Phylogenetic groups are highlighted in different colors.

Next, the taxonomic statuses of 21 unclassified genomes at the species level in the NCBI database were also re-evaluated. Based on our phylogenomic pipelines, nine previously unclassified genomes were assigned to an existing species, two were reclassified to other species, and ten were considered as new species (S6 Table). Finally, a phylogenomic tree was reconstructed with 109 strains of *Nocardia* (81 type strains and 28 novel taxa), allowing updating of the phylogeny of the genus *Nocardia* (Fig 4).

### Phylogenetic reconstruction using usual markers and novel loci

Although whole-genome sequencing can effectively differentiate *Nocardia* species and facilitate identifying unknown isolates, this approach is not yet feasible for routine use in a clinical laboratory, especially in developing countries. Therefore, we attempted to identify a single locus that allowed reliable species identification of *Nocardia*, as this is more affordable and easier to use than whole-genome sequencing. This gene needed to meet the following criteria: (i) single-copy gene; and (ii) good discriminatory power in comparison with traditional methods. We first reanalyzed the MLSA tree based on five commonly used markers (*gyrB*, *16S*, *secA1*, *hsp65*, and *rpoB*) from earlier studies [21]. Except for *secA1*, the remaining genes had multiple copies in some species, and *rpoB* was acquired via HGT events, leading to misidentification. The concatenated sequences of these five genes with a low nucleotide diversity value (below 0.075) reduced the discriminatory power and resulted in unstable subtrees with low bootstrap values (S5 Fig).

Thus, we searched among the genes of the core genome, built individual gene trees for all 384 single-copy genes, and compared their topologies with the phylogenomic tree topology as a reference. We observed that apart from the *dapb1* locus, a tree topology built from a single locus was not likely to agree with that of the phylogenomic tree. The *dapb1* gene, which encodes a 721 amino acid dipeptidyl aminopeptidase BI in *N*. *abscessus* NBRC 100374^T^, and is an enzyme for removing dipeptides from the amino-termini of peptides and proteins, was less susceptible to HGT and capable of reproducing a tree with similar topology (48 RFD and 85% of similarity) as our genome-based phylogeny [35]. The sequences of the *dapb1* gene had a high nucleotide diversity value (0.261) and yielded a tree that effectively separated the strains into the phylogenetic groups defined by genome-based phylogeny (Fig 2B).

This gene showed greater discriminatory power and yielded robust evolutionary relationships among species. It also provides a good target for developing sequence-based analysis or real-time PCR assays to detect *Nocardia* species. The discriminatory power of the *dapb1* locus made it possible to improve the accuracy of species identification within the genus *Nocardia*. To our knowledge, this is the first time that the *dapb1* gene has been used as a phylogenetic marker within a bacterial genus.

## Methods

### Strains and culture conditions

The three type strains and 27 reference strains of *Nocardia* used in this study were obtained from the DSMZ (Leibniz-Institut DSMZ-Deutsche Sammlung von Mikroorganismen und Zellkulturen GmbH, Brunswick, Germany). Additionally, 42 clinical strains were isolated from patients in China (S7 Table). Strains used in this study are available at the National Center for Human Pathogen Collection, Beijing, China. These isolates were stored at -80°C in brain heart infusion broth with 25% glycerol. Strains were grown on brain heart infusion agar with 10% sheep’s blood and incubated at 37°C with agitation for 48–72 h. Stains used in this study are available at the National Center for Human Pathogen Collection, Beijing, China.

### Genome sequencing, assembly, and annotation

The genomes of 72 *Nocardia* strains were sequenced. Genomic DNA was extracted using the Wizard Genomic DNA Purification Kit (Promega, Madison, WI, USA) following the manufacturer’s instructions. Whole-genome sequencing was performed using the Illumina NovaSeq platform in the PE150 mode to generate 350 bp paired-end read libraries using NEBNext DNA Library Prep Kit (New England Biolabs, USA). All sequencing depth exceeded 100-fold. Low-quality reads were filtered using the software readfq v10 if they met the following criteria: (i) reads containing more than 40 bp of low-quality bases (mass value ≤ 38); (ii) reads containing more than 10 bp of N bases; and (iii) reads with overlaps and adapter sequence exceeding 15 bp. All good-quality paired-end reads were the *de novo* assembled into 11 to 101 contigs using SPAdes v3.8.0 [36]. The annotation of sequenced genomes was performed using Prokka v1.13 [37].

### Downloading of publicly available assemblies and quality control

All genome sequences annotated as *Nocardia* were downloaded from the National Center for Biotechnology Information (NCBI) public database on 3 January 2020 using in house-scripts. All publicly available assemblies were subjected to quality control by Quast v5.0.2 [38]. Genomes with N75 values of < 10,000 bp and > 500 undetermined bases per 100,000 bases were discarded [39]. One *N*. *terpenica* genome (GCA_000320925.1) with an extremely large number of contigs was also discarded. Finally, 83 type strains, 53 validly published strains, and 21 unclassified *Nocardia* sp. passed these quality control checks. This resulted in a pool of 229 genomes of *Nocardia*.

### Pangenome analysis and functional annotation

The annotation GFF formatted files derived from Prokka were analyzed using Roary v3.13.0 [40]. Homologous genes are clustered into gene families with a minimum identity of 85%. Core genes were defined as those belonging to a gene family that was present in > 90% of the genomes analyzed. All gene families were scanned against a hidden Markov model (HMM) database of eggNOG (v5.0) profile HMMs using HMMER v3.3 [41].

### Identification of potential horizontal genes

The software HGTector v2.0 was used to determine the presence of horizontal genes using the cutoffs of 90% identity and an E value of 1e^-5^ [42]. The distribution of horizontal genes between *Nocardia* genomes and potential donors were identified and extracted from the HGTector output files.

### Phylogenetic tree construction of type stains

The coding sequences from all type strains were collected together, and a non-redundant homologous gene set was computed for them using CD-HIT v4.6.6 [43]. We then used BLAST 2.9.0+ to identify the homologous genes in the non-redundant homologous gene set. Here, if the homologous gene was present in 90% of the type strains and had just one copy in these strains, the gene was defined as a single-copy gene. The DNA sequences of all single-copy genes were aligned using clustalw2 and then merged. A final alignment was used to construct a maximum likelihood (ML) tree in iqtree v1.6.11 using the GTR+I+G model with 1000 bootstrap replicates [44]. The genome of *Rhodococcus globerulus* NBRC 14531^T^ (GCA_001894805.1) served as an outgroup. Bootstrap values were indicated on each node. The resulting phylogenetic tree was visualized using the R package ggtree v2.2.4 [45].

### Genome similarity assessment

Pairwise average nucleotide identity (ANI) values were estimated using a Perl script based on the methodology described by Li *et al* [46]. The *in silico* DNA-DNA hybridization (*is*DDH) values were calculated via the Genome-to-Genome Distance Calculator 2.1 (GGDC) (http://ggdc.dsmz.de/ggdc.php) using “Formula 2” [47]. The results were visualized and plotted with the R package pheatmap.

### Screening for phylogenetic markers

The nucleotide sequences of the 16S rRNA, *gyrB*, *secA1*, *hsp65*, and *rpoB* genes were extracted from the genomes of type strains. An MLSA tree was also constructed using individual alignments in the following order: *gyrB* (600 nt)– *16S* (462 nt)–*secA1* (426 nt)–*hsp65* (441 nt)–*rpoB* (438 nt). The phylogenetic trees of every single-copy gene were constructed using the maximum likelihood method with 1000 bootstrap repeats in iqtree v1.6.11.

The topological distances and similarities between our phylogenomic tree and 384 phylogenetic trees were computed using ete-compare v3.1.2 [48]. The nucleotide diversity (π) values of these genes were calculated using pegas in R [49]. The single-copy genes were ranked according to the Robinson–Foulds distance (RFD) and percentage of edge similarity and then according to their π values [50]. The functional annotation of each gene was obtained from the UniProt database.

## Conclusion

The present study evaluated the genetic diversity, the taxonomic position, and the evolutionary relationships of *Nocardia* based on pan-genome and comparative genomic analyses. The open pan-genome of *Nocardia* possesses extensive genetic diversity and a large and flexible gene repertoire. HGTs were drivers of genetic diversity that shaped the *Nocardia* pan-genome and core genome, which has made it difficult to distinguish this genus from other related taxa (especially *Rhodococcus*, *Skermania*, *Aldersonia*, and *Mycobacterium*). Furthermore, the phylogeny based on single-copy genes revealed five major phylogenetic groups, leading to the identification of five sets of type strains that could be merged. In addition, we discovered 28 potentially novel species and 16 reclassified species. Finally, we identified a novel locus for inferring the phylogeny of *Nocardia* that was more discriminatory and robust than other widely used markers, allowing it to be used for future molecular identification of these species.

## Supporting information

S1 Table Genome sequences of strains used in this study. (PDF)

S2 Table List of predicted horizontally transferred genes in core genome. (PDF)

S3 Table Complete list of 384 single copy genes. Robinson-Foulds distance and branch congruence measure were used to provide the differences and coincidences between single gene trees and phylogenomic tree. The single copy genes were ranked according to their topology similarity with the phylogenomic tree, and the nucleotide diversity was also taken into account.
(PDF)

S4 Table Average nucleotide identity values between type strains. (PDF)

S5 Table *In silico* DNA-DNA hybridization values between type strains. (PDF)

S6 Table Reclassification of genomes that are currently unclassified *Nocardia* species. (PDF)

S7 Table General features of sequenced *Nocardia* species. (PDF)

S1 Fig Genome size and GC content for the genus *Nocardia*. Different colors indicated taxonomic grouping as described in Fig 2.
(PDF)

S2 Fig Distribution of functional categories in the *Nocardia* unique genome. Genes with “Function unknown” were not included.
(PDF)

S3 Fig Distribution of functional categories for all group core genomes. (A) Functional categories for core gene families in each phylogroup. (B) The number of core gene families in each phylogroup.
(PDF)

S4 Fig Phylogenomic tree across 203 *Nocardia* strains. A maximum likelihood phylogenetic tree was constructed based on the concatenation of 241 single-copy genes of 81 type strains and 122 additional genomes of *Nocardia* spp. with 1000 bootstrap replicates using *Rhodococcus globerulus* NBRC 14531 as an outgroup. Bootstrap values are indicated on the nodes. Phylogenetic groups are highlighted in different colors. Red boxes indicate the same species has a type strain in different subgroup. The asterisk represents clusters lacking a type strain.
(PDF)

S5 Fig Phylogenetic tree of type strains of *Nocardia* based on the sequences of five commonly used markers. Phylogenetic tree was constructed by the maximum likelihood method based on the five concatenated gene sequences (*gyrB*, *16S*, *secA1*, *hsp65*, and *rpoB*) of 81 *Nocardia* type strains using *Rhodococcus globerulus* NBRC 14531 as an outgroup. Bootstrapping was carried out using 1000 replicates and values are shown at the nodes.
(PDF)
