# Supplementary material for: Whole genome sequencing reveals the genomic diversity, taxonomic classification, and evolutionary relationships of the genus Nocardia
Source: PLoS Negl Trop Dis. 2021 Aug 26;15(8):e0009665. doi: 10.1371/journal.pntd.0009665 (PMC8437295; doi:10.1371/journal.pntd.0009665)
Supplement: S1 Table — (PDF) [file pntd.0009665.s001.pdf]

S1 Table. Genome sequences of strains used in this study.

Nocardia	Strain	Genome Size (Mb)	No. of CDSs	%GC	Accession Number*	References
abscessus	DSM 44432^{T}	8.41	7811	68.2	GCA_000308455.1	[1]
abscessus	DSM 44557	8.08	7366	68.47	This study	[1]
abscessus	CDC 167	7.98	7209	68.37	This study	This study
acidivorans	DSM 45049^{T}	7.57	7110	66.9	GCA_001625085.1	This study
africana	DSM 44491^{T}	7.81	7124	67.9	GCA_001612635.1	[1]
africana	DSM 44500	7.73	7083	67.93	This study	This study
africana	DSM 44502	7.75	7319	67.91	GCA_900454335.1	This study
alba	DSM 44684^{T}	7.29	6613	67.7	GCA_004339125.1	This study
altamirensis	DSM 44997^{T}	9.83	8727	66.8	GCA_001612685.1	This study
amamiensis	DSM 45066^{T}	8.24	7818	67.4	GCA_001612745.1	[1]
amikacinitolerans	DSM 45539^{T}	7.64	6815	68.5	GCA_001612615.1	This study
amikacinitolerans	DSM 45538	7.63	6718	68.6	This study	This study
amikacinitolerans	DSM 45537	7.38	6579	68.68	GCA_900215415.1	This study
anaemiae	DSM 44821^{T}	8.62	7791	65.5	GCA_001612725.1	[1]
aobensis	DSM 44805^{T}	7.55	7010	68	GCA_000308375.1	[1]
araoensis	DSM 44729^{T}	7.72	7193	68.4	GCA_000308435.1	This study
arizonensis	DSM 45748^{T}	7.2	6547	67.9	GCA_001618405.1	This study
arizonensis	W9405	7.19	6584	67.94	GCA_001310275.1	This study
arthritidis	DSM 44731^{T}	7.12	6551	68.5	GCA_001612765.1	[1]
asiatica	DSM 44668^{T}	8.46	8008	68.4	GCA_000308415.1	[1]
asiatica	DSM 44700	8.19	7430	68.82	This study	This study
asiatica	CDC 193	8.92	8219	68.39	This study	This study
asiatica	CDC 194	7.66	6980	68.71	This study	This study
asteroides	DSM 43757^{T}	6.98	6422	69.9	GCA_900637185.1	[2]
asteroides	DSM 43258	7.57	6898	68.46	This study	This study
asteroides	DSM 43373	6.95	6371	69.92	GCA_900114685.1	This study
beijingensis	DSM 44636^{T}	7.48	6856	68.9	GCA_001612785.1	[1]
beijingensis	CDC 186	7.20	6574	69.13	This study	This study
beijingensis	CDC 188	7.44	6875	68.04	This study	This study
bhagyanarayanae	DSM 103495^{T}	7.56	6789	68.9	GCA_006716565.1	This study
blacklockiae	DSM 45135^{T}	8.29	7361	69.9	This study	This study
blacklockiae	CDC 190	8.10	7267	69.82	This study	This study
brasiliensis	DSM 43758^{T}	8.92	8080	68.2	GCA_900454265.1	[1]
brasiliensis	DSM 43009	9.58	8644	68.23	This study	This study
brasiliensis	DSM 46032	9.09	8198	68.27	This study	This study

brasiliensis	DSM 46059	9.30	8385	68.12	This study	This study
brasiliensis	CDC 144	9.39	8630	67.94	This study	This study
brasiliensis	CDC 163	9.91	8913	67.88	This study	This study
brasiliensis	CDC 196	9.39	8584	68.1	This study	This study
brasiliensis	HUJEG-1	9.44	8517	68.05	GCA_000250675.3	[3]
brasiliensis	FDAARGOS 352	8.94	8083	68.24	GCA_002209125.2	This study
brasiliensis	IFM 10847	9.15	8450	68.07	GCA_000710915.1	This study
brevicatena	DSM 43024^{T}	7.01	6646	67	GCA_000308495.1	[1]
caishijiensis	DSM 44831^{T}	6.29	5680	68.2	GCA_001612825.1	[1]
carnea	DSM 43397^{T}	7.5	6858	67.1	GCA_000308515.1	[1]
carnea	DSM 44558	7.88	7144	66.99	This study	[1]
carnea	DSM 44582	7.97	7219	66.98	This study	This study
carnea	DSM 46055	7.60	6973	67.09	This study	This study
carnea	DSM 46071	8.17	7451	67.05	This study	This study
carnea	NRRL B-1997	7.50	6871	67.09	GCA_000717405.1	This study
cerradoensis	DSM 44546^{T}	7.6	7135	68.2	GCA_000308535.1	[4]
cerradoensis	CNM20130759	8.99	8327	67.74	GCA_002236815.1	This study
concava	DSM 44804^{T}	8.93	8421	67.7	GCA_000308815.1	This study
coubleae	DSM 44960^{T}	6.62	6102	67.9	GCA_001612805.1	[1]
crassostreae	DSM 44597^{T}	8.29	7762	67.7	GCA_001613405.1	This study
cyriacigeorgica	DSM 44484^{T}	6.31	5676	68.2	GCA_005863225.1	[1]
cyriacigeorgica	DSM 40350	6.10	5524	68.48	This study	This study
cyriacigeorgica	DSM 43004	6.54	6149	68.38	This study	This study
cyriacigeorgica	DSM 43005	6.54	6144	68.38	This study	This study
cyriacigeorgica	DSM 43208	6.65	5934	68.26	This study	[1]
cyriacigeorgica	DSM 46058	6.36	5865	68.4	This study	This study
cyriacigeorgica	CDC 140	6.20	5569	68.38	This study	This study
cyriacigeorgica	CDC 156	6.68	6064	68.29	This study	This study
cyriacigeorgica	CDC 182	6.48	5826	68.38	This study	This study
cyriacigeorgica	CDC 197	6.49	5863	68.35	This study	This study
cyriacigeorgica	CDC 322	6.54	5857	68.26	This study	This study
cyriacigeorgica	CDC 323	6.42	5823	68.37	This study	This study
cyriacigeorgica	CDC 327	6.23	5616	68.46	This study	This study
cyriacigeorgica	CDC 332	6.21	5647	68.46	This study	This study
cyriacigeorgica	GUH-2	6.19	5572	68.37	GCA_000284035.1	[5]
cyriacigeorgica	3012STDY6756504	6.48	5779	68.24	GCA_900683635.1	This study
cyriacigeorgica	MDA3349	6.46	5836	68.28	GCA_002949635.1	This study
cyriacigeorgica	MDA3732	6.59	5935	68.22	GCA_002933455.1	This study
cyriacigeorgica	EML 446	6.83	5934	68.02	GCA_005863245.1	[6]
cyriacigeorgica	EML 1456	6.52	6226	68.23	GCA_005863295.1	[6]

donostiensis	DSM 46814^T	5.79	5190	66.6	GCA_002081715.1	[7]
donostiensis	X1655	5.79	5159	66.57	GCA_001989395.1	[7]
donostiensis	W9944	5.77	5102	66.59	GCA_002081795.1	[7]
elegans	DSM 44890^T	7.54	6937	67.9	GCA_001612845.1	This study
exalbida	DSM 44883^T	7.37	6772	68.6	GCA_000308575.1	[1]
farcinica	DSM 43665^T	6.46	6114	70.61	GCA_001182745.2	[1]
farcinica	DSM 46028	6.07	5640	70.93	This study	This study
farcinica	CDC 25	6.04	5607	70.92	This study	[8]
farcinica	CDC 27	6.26	5849	70.62	This study	[8]
farcinica	CDC 30	6.30	5888	70.61	This study	[8]
farcinica	CDC 31	6.26	5837	70.62	This study	[8]
farcinica	CDC 32	6.26	5848	70.62	This study	[8]
farcinica	CDC 35	6.30	5886	70.61	This study	[8]
farcinica	CDC 38	6.30	5884	70.61	This study	[8]
farcinica	CDC 42	6.11	5705	70.89	This study	[8]
farcinica	CDC 46	6.39	5929	70.72	This study	[8]
farcinica	CDC 48	6.59	6093	70.77	This study	[8]
farcinica	CDC 53	6.39	5934	70.72	This study	[8]
farcinica	CDC 56	6.33	5854	70.76	This study	[8]
farcinica	CDC 59	6.39	5929	70.72	This study	[8]
farcinica	CDC 65	6.14	5771	70.86	This study	[8]
farcinica	CDC 142	6.11	5693	70.72	This study	This study
farcinica	IFM 10152	6.29	5967	70.69	GCA_000009805.1	[9]
farcinica	W6977	6.43	6080	70.66	GCA_003367415.1	This study
farcinica	DSM 43257	6.41	6074	70.68	GCA_900156775.1	This study
farcinica	3012STDY6733949	6.14	5711	70.88	GCA_900683605.2	This study
farcinica	CNM20091955	6.73	6384	70.49	GCA_002571325.1	[10]
farcinica	CNM20080921	6.75	6430	70.48	GCA_002571345.1	[10]
farcinica	TRH1	5.23	4946	68	GCA_001704135.1	[11]
flavorosea	DSM 44480^T	7.44	6840	67.1	GCA_001613385.1	[1]
fluminea	DSM 44489^T	8.06	7597	67.5	GCA_002846365.1	[1]
fusca	NBRC 14340^T	8.04	7283	68.1	GCA_001618425.1	This study
gamkensis	DSM 44956^T	7.71	7093	68.4	GCA_001612985.1	[1]
grenadensis	DSM 45869^T	6.52	6014	68.2	GCA_001613445.1	This study
harenae	DSM 45095^T	6.14	5608	72	GCA_001612885.1	This study
higoensis	DSM 44732^T	6.98	5985	69.3	GCA_000308595.1	This study
ignorata	DSM 44496^T	7.02	6614	67.7	GCA_004362495.1	[1]
inohanensis	DSM 44667^T	8.12	7423	67.8	GCA_001612945.1	This study
inohanensis	CDC 153	8.22	7589	67.93	This study	This study
jejuensis	DSM 44959^T	8.65	7619	67.6	GCA_001613145.1	This study

jiangxiensis	DSM 17684[T]	10.45	9617	66.8	GCA_000308615.1	This study
jinanensis	DSM 45048[T]	5.04	4626	67.4	GCA_001482675.1	[12]
kruczakiae	DSM 44877[T]	7.32	6692	68.1	GCA_001612965.1	[1]
lijiangensis	DSM 44933[T]	8.16	7287	68.5	GCA_001613045.1	This study
mangyaensis	Y48[T]	7.31	6591	68.1	GCA_001886715.1	This study
mexicana	DSM 44952[T]	9	8161	68.7	GCA_003350525.1	This study
mikamii	DSM 45174[T]	7.56	6577	68	GCA_001613505.1	This study
miyunensis	DSM 17685[T]	10.52	8990	67	GCA_001613065.1	This study
neocaledoniensis	DSM 44717[T]	7.32	6798	69.7	GCA_003182135.1	[1]
niigatensis	DSM 44670[T]	8.22	7266	68.2	GCA_000308655.1	This study
ninae	DSM 44978[T]	9.7	8884	66.9	GCA_007990755.1	This study
niwae	DSM 45340[T]	7.3	6408	68.8	GCA_001613465.1	This study
nova	DSM 44481[T]	7.84	7203	67.9	GCA_001613005.1	[1]
nova	DSM 43207	7.86	7081	67.86	This study	[1]
nova	DSM 40806	7.85	7141	67.79	This study	This study
nova	DSM 43209	7.87	7090	67.86	This study	This study
nova	DSM 44559	7.81	7061	67.94	This study	This study
nova	CDC 141	7.86	7376	68.57	This study	This study
nova	CDC 159	7.87	7381	68.57	This study	This study
nova	SH22a	8.35	7579	67.77	GCA_000523235.1	[1]
nova	MDA3397	8.01	7299	67.86	GCA_002933545.1	This study
nova	ATCC 10905	7.85	7076	68.02	GCA_002933515.1	This study
nova	ATCC 33727	7.55	6939	67.91	GCA_003023865.1	This study
nova	BAA2227	7.97	7265	67.87	GCA_002933495.1	This study
nova	MDA5529	7.71	7032	68	GCA_002933575.1	This study
nova	MDA3139	7.49	7047	67.31	GCA_002933535.1	This study
nova	MDA0897	7.07	6565	67.46	GCA_002933465.1	This study
novocastrense	DSM 44692[T]	6.69	6308	71.7	This study	This study
otitidiscaviarum	DSM 43242[T]	7.54	6888	69	GCA_900454305.1	[1]
otitidiscaviarum	DSM 44565	7.33	6653	69.04	This study	[1]
otitidiscaviarum	DSM 43010	7.36	6729	68.99	This study	This study
otitidiscaviarum	DSM 43398	7.51	6766	69.03	This study	This study
otitidiscaviarum	CDC 155	7.25	6596	69.08	This study	This study
otitidiscaviarum	CDC 157	7.61	6922	68.97	This study	This study
otitidiscaviarum	CDC 195	7.30	6645	69.03	This study	This study
otitidiscaviarum	CDC 324	7.66	6938	68.97	This study	This study
otitidiscaviarum	CDC 326	7.57	6912	69.04	This study	This study
otitidiscaviarum	NEB252	7.69	6874	69.06	GCA_007362295.1	This study
otitidiscaviarum	IFM 11049	7.90	7410	68.88	GCA_000710895.1	[2]
paucivorans	DSM 44386[T]	6	5048	66.6	GCA_000308675.1	This study

panacis	YIM PH 21724^T	7.36	6802	67	GCA_003598715.1	[13]
pneumoniae	DSM 44730^T	7.59	6729	68.1	GCA_000308755.1	[1]
pseudobrasiliensis	DSM 44290^T	8.4	7895	67.3	GCA_003350585.1	[1]
pseudobrasiliensis	IFM 0761	8.39	7908	67.55	GCA_001304815.1	This study
pseudovaccinii	DSM 43406^T	9.94	9284	65.7	GCA_001613225.1	This study
puris	DSM 44599^T	7.7	7045	69.8	GCA_003315035.1	This study
rhamnosiphila	DSM 45147^T	7.66	6875	68.5	GCA_001613485.1	[1]
salmonicida	DSM 40472^T	8.25	7417	67	GCA_001613085.1	This study
salmonicida	NBRC 100378	7.50	6956	67.07	GCA_001612865.1	This study
salmonicida	NBRC 100376	7.55	7022	67.05	GCA_001613285.1	This study
seriolae	DSM 44129^T	7.6	7138	68.3	GCA_007990715.1	This study
seriolae	EM150506	8.30	7781	68.08	GCA_001865855.1	[14]
seriolae	UTF1	8.12	7648	68.14	GCA_002356035.1	[14]
seriolae	CK-14008	8.37	7893	68.09	GCA_001885535.1	[14]
seriolae	HSY-NS01	7.91	7441	68.19	GCA_003013595.1	This study
seriolae	SY-24	7.89	7446	68.21	GCA_002093935.1	[14]
seriolae	HSY-NS02	7.76	7274	68.23	GCA_003667075.1	This study
seriolae	ZJ0503	7.71	7268	68.25	GCA_000763165.1	[14]
seriolae	N-2927	7.76	7319	68.27	GCA_000583715.2	[14]
seriolae	U-1	7.77	7317	68.26	GCA_001192935.1	[14]
seriolae	KH 21	7.72	7262	68.26	GCA_009712555.1	This study
seriolae	PY 31	7.68	7241	68.28	GCA_009712525.1	This study
seriolae	VT 62	7.70	7260	68.28	GCA_009712405.1	This study
seriolae	VT 45	7.94	7506	68.2	GCA_009712575.1	This study
seriolae	NT 50	7.96	7515	68.17	GCA_009712505.1	This study
seriolae	KH 11	7.66	7249	68.28	GCA_009712515.1	This study
seriolae	PY 37	7.55	7236	68.29	GCA_009712525.1	This study
shimofusensis	DSM 44733^T	6.33	5670	69.1	GCA_001613125.1	This study
sienata	DSM 44766^T	6.84	6262	68.2	GCA_001613205.1	[1]
speluncae	DSM 45078^T	7.4	6765	66.9	GCA_001613245.1	This study
stercoris	NEAU-LL90^T	7.55	6670	69.1	GCA_003696265.1	This study
suismassiliense	S-137^T	9.47	8501	67.1	GCA_900269665.1	[15]
takedensis	DSM 44801^T	8.03	7206	69.5	GCA_000308695.1	This study
tenerifensis	DSM 44704^T	9.84	8917	68.5	GCA_003202065.1	This study
terpenica	IFM 0406	9.28	8681	68.5	GCA_001625105.1	This study
testacea	DSM 44765^T	7.27	6817	68.5	GCA_000308775.1	This study
thailandica	DSM 44808^T	6.82	6520	71.6	GCA_000308795.1	[1]
transvalensis	DSM 43405^T	8.38	7815	69.2	GCA_000308875.1	[1]
transvalensis	DSM 46068	8.72	8075	69.23	This study	[1]
uniformis	DSM 43136^T	8.77	8055	66	GCA_001613345.1	This study

vaccinii	DSM 43285^T	9.22	8405	66.7	GCA_001613305.1	This study
vaccinii	CDC 160	8.57	7787	67.45	This study	This study
vermiculata	DSM 44807^T	6.69	6157	67	GCA_001613265.1	This study
veterana	DSM 44445^T	6.79	6188	68.2	GCA_000308855.1	[1]
veterana	CDC 181	6.54	5890	68.29	This study	This study
vinacea	DSM 44638^T	10.16	9349	65.5	GCA_000308835.1	[1]
violaceofusca	NBRC 14427^T	7.51	6828	68	GCA_001613525.1	This study
vulneris	DSM 45737^T	9.37	8459	68.1	GCA_001613425.1	[16]
wallacei	DSM 45136^T	8.04	7325	69.1	This study	[1]
wallacei	DSM 45846	7.29	6479	69.48	This study	This study
xishanensis	DSM 44895^T	7.69	7049	68.4	GCA_001613365.1	This study
yamanashiensis	DSMZ 44669^T	9.1	8385	68.1	GCA_001613325.1	This study
yunnanensis	CFHS0054^T	7.93	7477	68.4	This study	[17]
Nocardia sp.	CS682	8.92	8117	67.26	GCA_004634405.1	[18]
Nocardia sp.	BMG51109	9.14	7726	69.19	GCA_000526215.1	This study
Nocardia sp.	W9405	7.19	6587	67.94	GCA_001310275.1	This study
Nocardia sp.	NRRL WC-3656	7.27	6598	67.93	GCA_000720265.1	[19]
Nocardia sp.	Root136	7.28	6710	67.32	GCA_001427185.1	This study
Nocardia sp.	852002-20019_SCH5090214	8.27	7573	67.88	GCA_001665865.1	This study
Nocardia sp.	852002-51101_SCH5132738	7.66	7079	67.85	GCA_001665695.1	This study
Nocardia sp.	852002-51244_SCH5132740	7.69	7087	67.9	GCA_001665525.1	This study
Nocardia sp.	FDAARGOS_372	6.46	6047	70.64	GCA_002554515.1	This study
Nocardia sp.	MDA0666	7.02	6380	68.01	GCA_003023875.1	This study
Nocardia sp.	SYSU K10002	9.37	8651	67.36	GCA_003123685.1	This study
Nocardia sp.	CICC 11023	10.03	9175	67.4	GCA_008704205.1	This study
Nocardia sp.	RB20	8.54	7517	67.22	GCA_009604405.1	This study
Nocardia sp.	RB56	8.62	7610	69.4	GCA_009604425.1	This study
Nocardia sp.	SYP-A9097	9.05	8293	65.81	GCA_009649815.1	This study
Nocardia sp.	CT2-14	7.38	6615	67.86	GCA_009708175.1	This study
Nocardia sp.	BMG111209	9.14	8068	69.19	GCA_000381925.1	[20]
Nocardia sp.	CNY236	5.3	4960	65.14	GCA_000482385.1	This study
Nocardia sp.	ET3-3	9.31	8681	68	GCA_009760405.1	This study
Nocardia sp.	348MFTsu5	5.71	5303	63.9	GCA_000383535.1	This study
Nocardia sp.	NRRL S-836	9.85	9058	70.56	GCA_001279525.1	This study

^{T} type strain

*The sequence data for newly sequenced isolates have been deposited in the GSA under accession number CRA003805.

Reference

1. McTaggart LR, Richardson SE, Witkowska M, Zhang SX. Phylogeny and Identification of *Nocardia* Species on the Basis of Multilocus Sequence Analysis. *J Clin Microbiol*. 2010;48: 4525–4533. doi:10.1128/JCM.00883-10

2. Komaki H, Ichikawa N, Hosoyama A, Takahashi-Nakaguchi A, Matsuzawa T, Suzuki K, *et al*. Genome based analysis of type-I polyketide synthase and nonribosomal peptide synthetase gene clusters in seven strains of five representative *Nocardia* species. *BMC Genomics*. 2014;15: 323. doi:10.1186/1471-2164-15-323

3. Vera-Cabrera L, Ortiz-Lopez R, Elizondo-Gonzalez R, Ocampo-Candiani J. Complete Genome Sequence Analysis of *Nocardia brasiliensis* HUJEG-1 Reveals a Saprobic Lifestyle and the Genes Needed for Human Pathogenesis. Gordon SV, editor. *PLoS ONE*. 2013;8: e65425. doi:10.1371/journal.pone.0065425

4. Carrasco G, Monzón S, Jiménez P, Cuesta I, Bartolomé-Alvarez J, Valdezate S. First Draft Genome Sequence of a Clinical Strain of *Nocardia cerradoensis*. *Genome Announc*. 2017;5. doi:10.1128/genomeA.00551-17

5. Zoropogui A, Pujic P, Normand P, Barbe V, Belli P, Graindorge A, et al. The *Nocardia cyriacigeorgica* GUH-2 genome shows ongoing adaptation of an environmental Actinobacteria to a pathogen's lifestyle. *BMC Genomics.* 2013;14: 286. doi:10.1186/1471-2164-14-286

6. Vautrin F, Bergeron E, Dubost A, Abrouk D, Martin C, Cournoyer B, *et al.* Genome Sequences of Three *Nocardia cyriacigeorgica* Strains and One *Nocardia asteroides* Strain. *Microbiol Resour Announc*. 2019;8: e00600-19. doi:10.1128/MRA.00600-19

7. Ercibengoa M, Bell M, Marimón JM, Humrighouse B, Klenk H-P, Pötter G, *et al*. *Nocardia donostiensis* sp. nov., isolated from human respiratory specimens. *Antonie Van Leeuwenhoek*. 2016;109: 653–660. doi:10.1007/s10482-016-0667-8

8. Du P, Hou X, Xie Y, Xu S, Li L, Zhang J, *et al*. Genotyping of *Nocardia farcinica* with multilocus sequence typing. *Eur J Clin Microbiol Infect Dis*. 2016;35: 771–778. doi:10.1007/s10096-016-2596-x

9. Ishikawa J, Yamashita A, Mikami Y, Hoshino Y, Kurita H, Hotta K, *et al*. The complete genomic sequence of *Nocardia farcinica* IFM 10152. *Proc Natl Acad Sci*. 2004;101: 14925–14930. doi:10.1073/pnas.0406410101

10. Valdezate S, Monzón S, Garrido N, Zaballos A, Medina-Pascual MJ, Azcona-Gutiérrez JM, *et al*. First Insight into the Genome Sequences of Two Linezolid-Resistant *Nocardia farcinica* Strains Isolated from Patients with Cystic Fibrosis. *Genome Announc*. 2017;5. doi:10.1128/genomeA.01212-17

11. Rodrigues EM, Vidigal PMP, Pylro VS, Morais DK, Leite LR, Roesch LFW, *et al*. Draft genome of *Nocardia farcinica* TRH1, a linear and polycyclic aromatic hydrocarbon-degrading bacterium isolated from the coast of Trindade Island, Brazil. *Brazilian Journal of Microbiology*. 2017;48: 391–392. doi:10.1016/j.bjm.2016.09.014

12. Chakrabortti A, Li J, Liang Z-X. Draft Genome Sequence of *Nocardia jinanensis*, an Opportunistic Bacterial Pathogen That Causes Cellulitis. *Genome Announc*. 2016;4. doi:10.1128/genomeA.00593-16

13. Hu J-Y, Li L, Peng G, Li Y-Q, Xu L-H, Guan H-L, *et al*. *Nocardia panacis* sp. nov., a novel actinomycete with antiphytopathogen activity isolated from the rhizosphere of Panax notoginseng. *Antonie van Leeuwenhoek*. 2020;113: 165–174. doi:10.1007/s10482-019-01326-z

14. Han H, Kwak M, Ha S, Yang S, Kim JD, Cho K, *et al.* Genomic characterization of *Nocardia seriolae* strains isolated from diseased fish. *MicrobiologyOpen*. 2019;8: e00656. doi:10.1002/mbo3.656

15. Fellag M, Levasseur A, Delerce J, Bittar F, Marié J-L, Davoust B, *et al.* Draft Genome Sequence of " *Nocardia suismassiliense* " Strain S-137 (CSUR P4007). *Genome Announc*. 2018;6: e00212-18. doi:10.1128/genomeA.00212-18

16. Lasker BA, Bell M, Klenk H-P, Spröer C, Schumann P, Brown JM. *Nocardia vulneris* sp. nov., isolated from wounds of human patients in North America. *Antonie van Leeuwenhoek*. 2014;106: 543–553. doi:10.1007/s10482-014-0226-0

17. Zhang J-X, Ming H, Zhao Z-L, Ji W-L, Chang X-L, Zhang L-Y, *et al*. *Nocardia yunnanensis* sp. nov., an actinomycete isolated from a soil sample. *Int J Syst Evol Microbiol*. 2019;69: 3116–3120. doi:10.1099/ijsem.0.003600

18. Dhakal D, Rayamajhi V, Nguyen HT, Poudel PB, Sohng JK. Complete Genome Sequence of *Nocardia* sp. Strain CS682, a Producer of Antibacterial Compound Nargenicin A1. *Microbiol Resour Announc*. 2019;8. doi:10.1128/MRA.01098-19

19. Ju K-S, Gao J, Doroghazi JR, Wang K-KA, Thibodeaux CJ, Li S, et al. Discovery of phosphonic acid natural products by mining the genomes of 10,000 actinomycetes. *Proc Natl Acad Sci*. 2015;112: 12175–12180. doi:10.1073/pnas.1500873112

20. Ghodhbane-Gtari F, Beauchemin N, Gueddou A, Hezbri K, Ktari A, Louati M, *et al*. Permanent Draft Genome Sequence of *Nocardia* sp. BMG111209, an Actinobacterium Isolated from Nodules of Casuarina glauca. *Genome Announc*. 2016;4. doi:10.1128/genomeA.00770-16.
