# Supplementary material for: Whole genome sequencing reveals the genomic diversity, taxonomic classification, and evolutionary relationships of the genus Nocardia
Source: PLoS Negl Trop Dis. 2021 Aug 26;15(8):e0009665. doi: 10.1371/journal.pntd.0009665 (PMC8437295; doi:10.1371/journal.pntd.0009665)
Supplement: S2 Table — (PDF) [file pntd.0009665.s002.pdf]

S2 Table. List of 107 predicted horizontally transferred genes in core genome

Protein accession number in *N.abscessus* NBRC 100374	Gene name	Genome type
WP_043688224.1	*atpD*	core
WP_011206964.1	*carD*	core
WP_043686876.1	*citA*	core
WP_043688668.1	*clpX*	core
WP_019044571.1	*crp*	core
WP_011206895.1	*cspA_2*	core
WP_086006611.1	*cysD_2*	core
WP_083899709.1	*dnaB*	core
WP_043689317.1	*ftsZ*	core
WP_028479292.1	*groS*	core
WP_083899637.1	NA	core
WP_040783886.1	NA	core
WP_043693618.1	NA	core
WP_085994820.1	NA	core
WP_174377715.1	*ilvC*	core
WP_039799659.1	*ilvH*	core
WP_011207082.1	*mbtH_2*	core
WP_043695871.1	*nrdF2*	core
WP_040777220.1	*pdtaR*	core
WP_043694364.1	*pdxS*	core
WP_043686699.1	*radD*	core
WP_043685451.1	*recR*	core
WP_043688208.1	*rho*	core
WP_043687358.1	*rplE*	core
WP_039795505.1	*rplN*	core
WP_039797323.1	*rplT*	core
WP_043688634.1	*rpmA*	core
WP_011211653.1	*rpmG2_2*	core
WP_043689602.1	*rpsA*	core
WP_043687383.1	*rpsE*	core
WP_039795549.1	*rpsH*	core
WP_174377687.1	*uvrB*	core
WP_011211115.1	*whiB1*	core
WP_029901515.1	*whiB2*	core
WP_043688075.1	*accD5_2*	core
WP_043699134.1	*aceA*	core
WP_039796380.1	*clpS*	core
WP_043699871.1	*dcd*	core
WP_011207048.1	*espR_2*	core
WP_043698829.1	*fusA*	core

WP_039799550.1	*glnB*	core
WP_040708791.1	NA	core
WP_039795739.1	NA	core
WP_011210678.1	NA	core
WP_011211259.1	NA	core
WP_040864988.1	*icd*	core
WP_003418601.1	*infA*	core
WP_083887487.1	*infC*	core
WP_043697976.1	*mprA_1*	core
WP_174315456.1	*mtrA*	core
WP_043699045.1	*nasF*	core
WP_043687244.1	*rplB*	core
WP_043698934.1	*rplK*	core
WP_040864842.1	*rplP*	core
WP_011210681.1	*rplS*	core
WP_011207266.1	*rplW*	core
WP_043698893.1	*rpoC*	core
WP_039798918.1	*rpoZ*	core
WP_043695197.1	*rpsB*	core
WP_030525248.1	*rpsG*	core
WP_003938093.1	*rpsJ*	core
WP_014981768.1	*rpsK*	core
WP_040870256.1	*rpsL*	core
WP_014981705.1	*rpsZ*	core
WP_043694466.1	*sigA*	core
WP_043694545.1	*sigB*	core
WP_043700355.1	*ssb_1*	core
WP_040782761.1	*sucD*	core
WP_043686740.1	*walR*	core
WP_043693711.1	*whiA*	core
WP_043699579.1	*dnaK_1*	core
WP_043699706.1	*groL1*	core
WP_043694830.1	NA	core
WP_040696222.1	NA	core
WP_043689200.1	NA	core
WP_039799153.1	NA	core
WP_043700314.1	*ino1*	core
WP_174186519.1	*pafA*	core
WP_043694706.1	*recA*	core
WP_043699059.1	*regX3_2*	core
WP_043687917.1	*ricR*	core
WP_011210730.1	*rpmB_2*	core
WP_039796334.1	*rpmE*	core
WP_039796155.1	*rpoA*	core

WP_043687550.1	*rpsM*	core
WP_011211513.1	*rpsR1*	core
WP_043687250.1	*rpsS*	core
WP_043698828.1	*tuf*	core
WP_043699980.1	*aspC_1*	core
WP_043686633.1	*pstB1*	core
WP_043688437.1	*rph*	core
WP_040864865.1	*rplX*	core
WP_019049259.1	*rpmG2_1*	core
WP_043687258.1	*rpsC*	core
WP_028476708.1	NA	core
WP_043698986.1	*menB*	core
WP_043695887.1	*nrdE1*	core
WP_043693088.1	*arc*	core
WP_043689484.1	*bioB*	core
WP_043688091.1	*purE*	core
WP_043693149.1	*rsbRA*	core
WP_043698018.1	*sucC*	core
WP_043687553.1	*rpsD*	core
WP_043699502.1	*purA*	core
NA	*rpoB*	core
WP_043696209.1	*smpB*	core
WP_043697694.1	*greA*	core
