# Supplementary material for: Whole genome sequencing reveals the genomic diversity, taxonomic classification, and evolutionary relationships of the genus Nocardia
Source: PLoS Negl Trop Dis. 2021 Aug 26;15(8):e0009665. doi: 10.1371/journal.pntd.0009665 (PMC8437295; doi:10.1371/journal.pntd.0009665)
Supplement: S3 Table — Robinson-Foulds distance and branch congruence measure were used to provide the differences and coincidences between single gene trees and phylogenomic tree. The single copy genes were ranked according to their topology similarity with the phylogenomic tree, and the nucleotide diversity was also taken into account. (PDF) [file pntd.0009665.s003.pdf]

S3 Table. Complete list of the 384 single copy genes.

Protein accession number in *N. abscessus* NBRC 100374	Gene name	RFD	Branch congruence measure (%)	π	Description
WP_083899681.1	NA	34	0.76	0.309	prolyl oligopeptidase family serine peptidase
WP_043696244.1	*mmgC*	34	0.76	0.276	Acyl-CoA dehydrogenase
WP_043695513.1	*ppk*	36	0.77	0.305	Polyphosphate kinase
WP_043685774.1	*topA*	36	0.77	0.295	DNA topoisomerase
WP_043699045.1	*cysG*	36	0.77	0.283	Siroheme synthase
WP_043700493.1	NA	36	0.74	0.33	putative ABC transporter
WP_043699130.1	*metE*	38	0.88	0.313	5-methyltetrahydropteroyltriglutamate--homocysteine methyltransferase
WP_051169005.1	*ndh*	40	0.72	0.32	NADH dehydrogenase
WP_043686035.1	NA	42	0.73	0.338	trehalose-phosphatase
WP_043694756.1	*ftsK*	42	0.73	0.303	DNA translocase
WP_051169366.1	*hemL*	42	0.73	0.303	Glutamate-1-semialdehyde 2,1-aminomutase
WP_043700199.1	*leuS*	42	0.73	0.285	Leucine--tRNA ligase
WP_051168648.1	*folC*	42	0.72	0.303	Dihydrofolate synthase
WP_043685249.1	NA	42	0.71	0.309	Fatty acid ABC transporter
WP_043692156.1	*mshC*	42	0.7	0.316	L-cysteine:1D-myo-inositol 2-amino-2-deoxy-alpha-D-glucopyranoside ligase
WP_157229694.1	*nuoG*	42	0.7	0.313	NADH-quinone oxidoreductase subunit G
WP_043689770.1	*menE*	44	0.86	0.325	2-succinylbenzoate--CoA ligase
WP_043697332.1	*glgC*	44	0.72	0.31	Glucose-1-phosphate adenylyltransferase
WP_043685900.1	*lysS1*	44	0.71	0.312	Lysine--tRNA ligase 1
WP_043685154.1	NA	44	0.71	0.31	cysteine desulfurase-like protein
WP_043699618.1	NA	44	0.71	0.31	HAD-IB family hydrolase
WP_043699464.1	*pta*	44	0.7	0.319	Phosphate acetyltransferase
WP_043695811.1	NA	44	0.7	0.315	Glycogen synthase
WP_043694528.1	NA	44	0.69	0.304	DEAD/DEAH box helicase
WP_174377743.1	NA	46	0.84	0.306	HAMP domain-containing protein
WP_051169086.1	*hflX*	46	0.71	0.302	GTPase HflX
WP_174377687.1	*uvrB*	46	0.71	0.296	UvrABC system protein B
WP_043689634.1	*uvrA*	46	0.71	0.293	UvrABC system protein A
WP_043685381.1	NA	46	0.71	0.288	Putative aminotransferase
WP_043697701.1	*cbs*	46	0.71	0.281	Putative cystathionine beta-synthase
WP_086006697.1	NA	46	0.7	0.311	acyltransferase
WP_043688567.1	*pepN*	46	0.7	0.301	Aminopeptidase N
WP_043687657.1	*glmS*	46	0.7	0.285	Glutamine--fructose-6-phosphate aminotransferase
WP_043687634.1	NA	46	0.69	0.326	ABC transporter
WP_043685622.1	*mtgA*	46	0.69	0.318	Biosynthetic peptidoglycan transglycosylase
WP_043697400.1	*fbiC*	46	0.69	0.313	FO synthase

WP_043697624.1	*fumC*	46	0.68	0.328	Fumarate hydratase class II
WP_043688748.1	NA	46	0.68	0.318	HlyC/CorC family transporter
WP_043692891.1	*cobN*	46	0.68	0.306	Aerobic cobaltochelatase subunit CobN
WP_043689536.1	*ilvA*	46	0.68	0.298	L-threonine dehydratase biosynthetic IlvA
WP_043684896.1	*embB*	46	0.67	0.309	putative arabinosyltransferase B
WP_043688262.1	*glgE*	46	0.67	0.306	Alpha-1,4-glucan:maltose-1-phosphate maltosyltransferase
WP_043686561.1	*dapb1*	48	0.85	0.261	Dipeptidyl aminopeptidase BI
WP_083899718.1	*lon*	48	0.84	0.287	Lon protease
WP_043693099.1	*metH*	48	0.7	0.285	Methionine synthase
WP_043688201.1	*argS*	48	0.69	0.305	Arginine--tRNA ligase
WP_043697784.1	*mfd*	48	0.69	0.3	Transcription-repair-coupling factor
WP_043696400.1	*pgm*	48	0.69	0.299	Phosphoglucomutase
WP_043695237.1	*yhgF*	48	0.69	0.288	Protein YhgF
WP_043685835.1	*dltA*	48	0.68	0.333	D-alanine--D-alanyl carrier protein ligase
WP_043699061.1	*sasA*	48	0.68	0.312	Adaptive-response sensory-kinase
WP_043696736.1	*pdtaS*	48	0.67	0.323	putative sensor histidine kinase
WP_174377666.1	*deoA*	48	0.67	0.321	Thymidine phosphorylase
WP_043688990.1	NA	48	0.67	0.32	helix-turn-helix domain-containing protein
WP_043685247.1	NA	48	0.67	0.318	putative ABC transporter
WP_043688272.1	*dinG*	48	0.67	0.315	putative ATP-dependent helicase
WP_043697950.1	*moeA1*	48	0.67	0.307	Molybdopterin molybdenumtransferase 1
WP_043699575.1	*dnaJ1*	48	0.67	0.297	Chaperone protein DnaJ 1
WP_043688260.1	*glgB*	48	0.66	0.316	1,4-alpha-glucan branching enzyme
WP_043697629.1	NA	48	0.66	0.315	PhoH family protein
WP_043693849.1	*pyrC*	48	0.66	0.303	Dihydroorotase
WP_043688778.1	dnaG	48	0.66	0.3	DNA primase
WP_043700394.1	NA	48	0.66	0.268	ABC transporter substrate-binding protein
WP_043695332.1	*frr*	50	0.83	0.296	Ribosome-recycling factor
WP_043696697.1	*moeZ*	50	0.83	0.278	adenylyltransferase/sulfurtransferase
WP_043696894.1	*algC*	50	0.68	0.316	Phosphomannomutase/phosphoglucomutase
WP_083899274.1	*polA*	50	0.68	0.313	DNA polymerase I
WP_043697326.1	*mrp*	50	0.68	0.292	Iron-sulfur cluster carrier protein
WP_043693088.1	*arc*	50	0.68	0.286	Proteasome-associated ATPase
WP_043686502.1	*purL*	50	0.68	0.269	Phosphoribosylformylglycinamidine synthase
WP_043688202.1	*lysA*	50	0.67	0.294	Diaminopimelate decarboxylase
WP_043684743.1	*serS*	50	0.67	0.29	Serine--tRNA ligase
WP_174377697.1	*dapE*	50	0.67	0.285	Succinyl-diaminopimelate desuccinylase
WP_043686010.1	*radA*	50	0.66	0.32	DNA repair protein RadA
WP_043697651.1	*NA*	50	0.66	0.32	hypothetical protein
WP_043694444.1	*kimA*	50	0.66	0.31	Potassium transporter KimA
WP_043698038.1	*uvrD1*	50	0.66	0.303	ATP-dependent DNA helicase UvrD1
WP_043689746.1	*argJ*	50	0.66	0.294	Arginine biosynthesis bifunctional protein

WP_174377695.1	*nuoD*	50	0.65	0.288	NADH-quinone oxidoreductase subunit D
WP_169334193.1	*gmk*	52	0.82	0.322	Guanylate kinase
WP_043697370.1	*dapD*	52	0.82	0.305	2,3,4,5-tetrahydropyridine-2,6-dicarboxylate N-succinyltransferase
WP_043685134.1	*glfT2*	52	0.67	0.32	Galactofuranosyltransferase GlfT2
WP_043697614.1	*glpX*	52	0.67	0.281	Fructose-1,6-bisphosphatase class 2
WP_043692966.1	*dop*	52	0.67	0.275	Pup deamidase/depupylase
WP_157229688.1	*secA1*	52	0.67	0.26	Protein translocase subunit SecA 1
WP_051168438.1	NA	52	0.66	0.312	FAD-binding oxidoreductase
WP_043699206.1	*ramB*	52	0.66	0.311	HTH-type transcriptional regulator
WP_043693670.1	*tkt*	52	0.66	0.271	Transketolase
WP_043684435.1	*pknB*	52	0.65	0.35	Serine/threonine-protein kinase
WP_043687599.1	*eccC*	52	0.65	0.322	ESX secretion system protein
WP_043689046.1	*glnE*	52	0.65	0.307	Bifunctional glutamine synthetase adenylyltransferase
WP_051169285.1	NA	52	0.64	0.324	EAL domain-containing protein
WP_043697695.1	*metB*	52	0.64	0.313	Cystathionine gamma-synthase
WP_043695755.1	*ligA*	52	0.64	0.301	DNA ligase A
WP_043699448.1	*btuD*	52	0.63	0.312	Vitamin B12 import ATP-binding protein
WP_040865365.1	NA	52	0.63	0.304	TetR family transcriptional regulator
WP_043685860.1	*gltC*	52	0.63	0.299	HTH-type transcriptional regulator
WP_051168722.1	*pheS*	52	0.63	0.286	Phenylalanine--tRNA ligase alpha subunit
WP_086006622.1	*pyk*	54	0.65	0.304	Pyruvate kinase
WP_043686458.1	*purB*	54	0.65	0.288	Adenylosuccinate lyase
WP_043699502.1	*purA*	54	0.65	0.282	Adenylosuccinate synthetase
WP_083899251.1	*ctaC*	54	0.65	0.279	Cytochrome c oxidase subunit 2
WP_043699749.1	NA	54	0.65	0.274	Acyl-CoA dehydrogenase fadE12
WP_043690243.1	NA	54	0.64	0.321	HlyC/CorC family transporter
WP_043689852.1	*der*	54	0.64	0.314	GTPase Der
WP_043694562.1	NA	54	0.64	0.31	DEAD/DEAH box helicase
WP_051168778.1	*gcvP*	54	0.64	0.309	putative glycine dehydrogenase
WP_043697796.1	*glmU*	54	0.64	0.308	Bifunctional protein
WP_043690683.1	NA	54	0.64	0.294	SDR family oxidoreductase
WP_043693348.1	NA	54	0.64	0.292	coenzyme F420-dependent oxidoreductase
WP_043687596.1	*eccD5*	54	0.63	0.306	ESX-5 secretion system protein
WP_051168435.1	*tgt*	54	0.61	0.292	Queuine tRNA-ribosyltransferase
WP_043684840.1	NA	56	0.81	0.295	Putative peptide transport permease protein
WP_043685876.1	*ftsH*	56	0.65	0.257	ATP-dependent zinc metalloprotease
WP_043698046.1	*pgi*	56	0.64	0.3	Glucose-6-phosphate isomerase
WP_051168485.1	*otsA*	56	0.64	0.297	Trehalose-6-phosphate synthase
WP_043687877.1	NA	56	0.64	0.291	hypothetical protein
WP_043689564.1	*trpC*	56	0.64	0.29	Indole-3-glycerol phosphate synthase
WP_043688667.1	*tig*	56	0.64	0.272	Trigger factor
WP_043688724.1	*fprB*	56	0.63	0.329	putative ferredoxin

WP_169334154.1	*fadD3*	56	0.63	0.325	CoA ligase
WP_043689786.1	*tyrS*	56	0.63	0.324	Tyrosine--tRNA ligase
WP_043700485.1	*sdhA*	56	0.63	0.315	Succinate dehydrogenase flavoprotein
WP_043686516.1	*purF*	56	0.63	0.312	Amidophosphoribosyltransferase
WP_043700203.1	NA	56	0.63	0.312	hypothetical protein
WP_043686050.1	NA	56	0.63	0.309	lipid-transfer protein
WP_043689562.1	*trpE*	56	0.63	0.303	Anthranilate synthase component 1
WP_043688232.1	*murA*	56	0.62	0.336	UDP-N-acetylglucosamine 1-carboxyvinyltransferase
WP_043684354.1	*yidC*	56	0.62	0.31	Membrane protein insertase
WP_043689172.1	*pepA*	56	0.62	0.307	putative cytosol aminopeptidase
WP_043686047.1	NA	56	0.62	0.284	Acyl-CoA dehydrogenase FadE29
WP_083899478.1	*aftC*	56	0.61	0.322	Alpha-(1->3)-arabinofuranosyltransferase
WP_043685625.1	NA	56	0.61	0.316	ArsA family ATPase
WP_043688017.1	*scmP*	56	0.61	0.312	N-acetylcysteine deacetylase
WP_043694282.1	NA	56	0.61	0.306	Phosphatidylinositol phosphate synthase
WP_086006729.1	NA	56	0.61	0.286	hypothetical protein
WP_043689398.1	NA	56	0.6	0.319	putative RNA pseudouridine synthase
WP_043686215.1	NA	58	0.81	0.316	Putative acyl-CoA dehydrogenase FadE17
WP_043699247.1	*lpdC*	58	0.81	0.256	Dihydrolipoyl dehydrogenase
WP_051168500.1	NA	58	0.8	0.339	DUF3071 domain-containing protein
WP_043684511.1	*gltD*	58	0.63	0.259	Glutamate synthase [NADPH] small chain
WP_043694934.1	*pnp*	58	0.63	0.253	Polyribonucleotide nucleotidyltransferase
WP_043691023.1	*nuoL*	58	0.62	0.323	NADH-quinone oxidoreductase subunit L
WP_086006780.1	*nuoM*	58	0.62	0.313	NADH-quinone oxidoreductase subunit M
WP_174377710.1	*rnj*	58	0.62	0.294	Ribonuclease J
WP_039798216.1	NA	58	0.62	0.288	DUF3090 domain-containing protein
WP_043693674.1	*zwf2*	58	0.62	0.288	Glucose-6-phosphate 1-dehydrogenase 2
WP_043693840.1	*carB*	58	0.62	0.279	Carbamoyl-phosphate synthase large chain
WP_043688075.1	*accD5*	58	0.62	0.249	putative propionyl-CoA carboxylase beta chain 5
WP_043694034.1	*aroC*	58	0.61	0.316	Chorismate synthase
WP_043695388.1	NA	58	0.61	0.314	chromosome segregation protein SMC
WP_043689835.1	*pyrG*	58	0.61	0.309	CTP synthase
WP_043688210.1	*prfA*	58	0.61	0.298	Peptide chain release factor 1
WP_086006598.1	*purK*	58	0.6	0.317	N5-carboxyaminoimidazole ribonucleotide synthase
WP_043690194.1	NA	58	0.6	0.314	acyl-CoA dehydrogenase family protein
WP_086006663.1	NA	58	0.6	0.314	putative AAA domain-containing protein
WP_043688744.1	*dnaJ2*	58	0.6	0.3	Chaperone protein
WP_043689528.1	*hisF*	58	0.6	0.296	Imidazole glycerol phosphate synthase subunit
WP_051169357.1	*hepT*	58	0.6	0.285	Heptaprenyl diphosphate synthase component

WP_043699333.1	*nuoL*	58	0.59	0.344	NADH-quinone oxidoreductase subunit L
WP_043694274.1	*tesB*	58	0.59	0.311	Acyl-CoA thioesterase 2
WP_174377696.1	*nuoC*	58	0.59	0.308	NADH-quinone oxidoreductase subunit C
WP_043688530.1	*orn*	58	0.59	0.297	Oligoribonuclease
WP_043689177.1	*ilvE*	58	0.59	0.296	Branched-chain-amino-acid aminotransferase
WP_043686360.1	NA	58	0.59	0.291	3-oxocholest-4-en-26-oate--CoA ligase
WP_043693706.1	*pgk*	58	0.59	0.281	Phosphoglycerate kinase
WP_043685144.1	*tagH*	60	0.81	0.315	Teichoic acids export ATP-binding protein
WP_043697127.1	*deaD*	60	0.62	0.304	ATP-dependent RNA helicase
WP_043692933.1	*helY*	60	0.62	0.303	putative helicase
WP_043694117.1	*alaS*	60	0.62	0.282	Alanine--tRNA ligase
WP_043689044.1	*glnA2*	60	0.62	0.281	Glutamine synthetase
WP_086006710.1	*metG*	60	0.62	0.274	Methionine--tRNA ligase
WP_043685491.1	*leuA*	60	0.62	0.254	2-isopropylmalate synthase
WP_086006727.1	*tldD*	60	0.61	0.308	Metalloprotease
WP_043695061.1	*mqo*	60	0.61	0.302	putative malate:quinone oxidoreductase
WP_083899255.1	*qcrC*	60	0.61	0.294	Cytochrome bc1 complex cytochrome c subunit
WP_043685439.1	NA	60	0.61	0.29	putative fatty acid methyltransferase
WP_043695674.1	*gatA*	60	0.61	0.287	Glutamyl-tRNA(Gln) amidotransferase subunit A
WP_083899151.1	*guaB*	60	0.61	0.284	Inosine-5'-monophosphate dehydrogenase
WP_043695018.1	*nusA*	60	0.61	0.281	Transcription termination/ antitermination protein
WP_043696645.1	*nasD*	60	0.61	0.26	Nitrite reductase
WP_043695541.1	*leuB*	60	0.6	0.269	3-isopropylmalate dehydrogenase
WP_043687896.1	*lpdA*	60	0.58	0.313	NAD(P)H dehydrogenase
WP_043686520.1	*purM*	60	0.58	0.305	Phosphoribosylformylglycinamidine cyclo-ligase
WP_043691073.1	*nuoB*	60	0.58	0.304	NADH-quinone oxidoreductase subunit B
WP_043686699.1	NA	62	0.8	0.32	DEAD/DEAH box helicase
WP_043700026.1	*hcaB*	62	0.8	0.276	3-phenylpropionate-dihydrodiol/cinnamic acid-dihydrodiol dehydrogenase
WP_043688750.1	*era*	62	0.79	0.279	GTPase Era
WP_086006615.1	*aceE*	62	0.61	0.28	Pyruvate dehydrogenase E1 component
WP_043688253.1	NA	62	0.6	0.313	hypothetical protein
WP_043698004.1	*purH*	62	0.6	0.309	Bifunctional purine biosynthesis protein
WP_043684864.1	*glfT2*	62	0.6	0.286	Galactofuranosyltransferase
WP_043684752.1	NA	62	0.6	0.278	Putative Rieske 2Fe-2S iron-sulfur protein
WP_043698893.1	*rpoC*	62	0.6	0.24	DNA-directed RNA polymerase subunit beta
WP_043688692.1	*proA*	62	0.59	0.303	Gamma-glutamyl phosphate reductase
WP_043685537.1	*asd*	62	0.59	0.287	Aspartate-semialdehyde dehydrogenase
WP_043693703.1	*tpiA*	62	0.59	0.272	Triosephosphate isomerase

WP_051169146.1	*ndh*	62	0.58	0.292	NADH dehydrogenase
WP_043695076.1	*map*	62	0.58	0.281	Methionine aminopeptidase 2
WP_043686644.1	*dus*	62	0.57	0.318	putative tRNA-dihydrouridine synthase
WP_043694623.1	*lexA*	62	0.56	0.331	LexA repressor
WP_043686743.1	*phoU2*	62	0.56	0.299	Phosphate-specific transport system accessory protein
WP_043699540.1	*fabH*	62	0.56	0.298	3-oxoacyl-[acyl-carrier-protein] synthase 3
WP_051168471.1	NA	64	0.79	0.29	Putative enoyl-CoA hydratase EchA13
WP_083899527.1	*gatB*	64	0.59	0.275	Aspartyl/glutamyl-tRNA(Asn/Gln) amidotransferase subunit B
WP_043684862.1	*glf*	64	0.59	0.27	UDP-galactopyranose mutase
WP_043695803.1	*etfA*	64	0.59	0.257	Electron transfer flavoprotein subunit alpha
WP_043690371.1	*mutB*	64	0.59	0.246	putative methylmalonyl-CoA mutase large subunit
WP_043685147.1	NA	64	0.58	0.327	ABC transporter permease
WP_043686876.1	*citA*	64	0.58	0.306	Putative citrate synthase 2
WP_083899464.1	*ctaB*	64	0.58	0.297	Protoheme IX farnesyltransferase
WP_043689123.1	*lipA*	64	0.58	0.273	Lipoyl synthase
WP_043689651.1	*pheT*	64	0.58	0.238	Phenylalanine--tRNA ligase beta subunit
WP_043694554.1	*ideR*	64	0.57	0.311	Iron-dependent repressor
WP_043690337.1	*guaB1*	64	0.57	0.31	putative oxidoreductase
WP_043694157.1	NA	64	0.57	0.307	hypothetical protein
WP_043688534.1	*fadH*	64	0.57	0.306	2,4-dienoyl-CoA reductase
WP_043689521.1	*hisC*	64	0.57	0.302	Histidinol-phosphate aminotransferase
WP_043698178.1	*acdA*	64	0.57	0.266	Acyl-CoA dehydrogenase
WP_043688949.1	*ahpE*	64	0.57	0.25	Alkyl hydroperoxide reductase E
WP_043699071.1	*gpmA*	64	0.56	0.281	2,3-bisphosphoglycerate-dependent phosphoglycerate mutase
WP_043690208.1	NA	64	0.55	0.302	bifunctional nuclease family protein
WP_051168991.1	*fumA*	66	0.58	0.278	Fumarate hydratase class I, aerobic
WP_043697798.1	*prs*	66	0.58	0.275	Ribose-phosphate pyrophosphokinase
WP_039803160.1	NA	66	0.58	0.271	helix-turn-helix transcriptional regulator
WP_043694364.1	*pdxS*	66	0.58	0.268	Pyridoxal 5'-phosphate synthase subunit
WP_043684377.1	*gyrA*	66	0.58	0.265	DNA gyrase subunit A
WP_083899118.1	*gltA2*	66	0.58	0.242	Citrate synthase 1
WP_083899588.1	*kgd*	66	0.58	0.24	Multifunctional 2-oxoglutarate metabolism enzyme
WP_043686827.1	*serC*	66	0.57	0.293	Phosphoserine aminotransferase
WP_043689279.1	*aroG*	66	0.57	0.291	Phospho-2-dehydro-3-deoxyheptonate aldolase
WP_043689340.1	*qcrA*	66	0.57	0.283	Cytochrome bc1 complex Rieske iron-sulfur subunit
WP_043694071.1	*aspS*	66	0.57	0.269	Aspartate—tRNA ligase
WP_043694706.1	*recA*	66	0.57	0.261	recombinase RecA

WP_040864988.1	*icd*	66	0.57	0.237	Isocitrate dehydrogenase
WP_157229656.1	NA	66	0.55	0.336	putative 1,4-alpha-glucan branching enzyme
WP_043689789.1	*argC*	66	0.55	0.301	N-acetyl-gamma-glutamyl-phosphate reductase
WP_169334158.1	*dnaE2*	66	0.54	0.316	Error-prone DNA polymerase
WP_043693886.1	*ypdF*	66	0.54	0.302	Aminopeptidase
WP_043693056.1	*trmI*	68	0.76	0.316	tRNA (adenine(58)-N(1))-methyltransferase
WP_043695779.1	*mnmA*	68	0.56	0.329	tRNA-specific 2-thiouridylase MnmA
WP_043697819.1	*prfC*	68	0.56	0.308	Peptide chain release factor 3
WP_043697729.1	*eno*	68	0.56	0.275	Enolase
WP_043696447.1	*prfB*	68	0.56	0.273	Peptide chain release factor 2
WP_043699520.1	*fba*	68	0.56	0.259	Fructose-bisphosphate aldolase
WP_083899705.1	*sugA*	68	0.55	0.299	Trehalose transport system permease protein
WP_043695032.1	*proS*	68	0.55	0.273	Proline--tRNA ligase
WP_043687229.1	*crtL*	68	0.54	0.33	Lycopene beta cyclase
WP_043686342.1	NA	68	0.54	0.326	thiolase domain-containing protein
WP_043695259.1	*ffh*	68	0.54	0.272	Signal recognition particle protein
WP_043689134.1	*dlaT*	68	0.53	0.249	Dihydrolipoyllysine-residue acetyltransferase component of pyruvate dehydrogenase complex
WP_043699047.1	*hemA*	68	0.52	0.362	Glutamyl-tRNA reductase
WP_043689775.1	*wecE*	68	0.52	0.315	dTDP-4-amino-4,6-dideoxygalactose transaminase
WP_043685128.1	NA	68	0.52	0.29	glycosyltransferase
WP_043694150.1	NA	70	0.55	0.307	membrane protein
WP_043700371.1	NA	70	0.55	0.29	Putative monooxygenase
WP_043689499.1	*nadA*	70	0.55	0.279	Quinolinate synthase A
WP_043689484.1	*bioB*	70	0.55	0.266	Biotin synthase
WP_043686604.1	NA	70	0.55	0.233	Putative thiosulfate sulfurtransferase
WP_043695829.1	*COQ5*	70	0.52	0.307	2-methoxy-6-polyprenyl-1,4-benzoquinol methylase, mitochondrial
WP_043688733.1	*cysNC*	70	0.51	0.303	Bifunctional enzyme CysN/CysC
WP_043699605.1	*hemB*	70	0.5	0.289	Delta-aminolevulinic acid dehydratase
WP_043688721.1	*sbpA*	70	0.49	0.276	Sulfate-binding protein
WP_043688668.1	*clpX*	72	0.54	0.282	ATP-dependent Clp protease subunit
WP_043694271.1	NA	72	0.54	0.252	putative transcriptional regulatory protein
WP_043688958.1	*accD6*	72	0.53	0.3	putative propionyl-CoA carboxylase beta chain 6
WP_043694066.1	NA	72	0.53	0.295	kinase
WP_043693440.1	NA	72	0.53	0.282	SPFH/Band 7/PHB domain protein
WP_039796155.1	*rpoA*	72	0.53	0.251	RNA polymerase subunit alpha
WP_040780648.1	*pyrH*	72	0.52	0.279	Uridylate kinase
WP_043693844.1	*carA*	72	0.51	0.299	Carbamoyl-phosphate synthase small chain
WP_043689630.1	NA	72	0.51	0.293	Universal stress protein

WP_051169214.1	NA	72	0.49	0.315	sigma-70 family RNA polymerase sigma factor
WP_043688208.1	*rho*	74	0.53	0.277	Transcription termination factor Rho
WP_043694380.1	*thrS*	74	0.53	0.272	Threonine--tRNA ligase
WP_083899664.1	*nusG*	74	0.53	0.269	Transcription termination/antitermination protein
WP_043687244.1	*rplB*	74	0.53	0.264	50S ribosomal protein L2
WP_043700314.1	*ino1*	74	0.53	0.263	Inositol-3-phosphate synthase
WP_043688551.1	*ettA*	74	0.53	0.244	Energy-dependent translational throttle protein
WP_043695806.1	*etfB*	74	0.53	0.232	Electron transfer flavoprotein subunit beta
WP_157229882.1	*thiC*	74	0.52	0.287	Phosphomethylpyrimidine synthase
WP_051168563.1	*sdhA*	74	0.52	0.236	Succinate dehydrogenase flavoprotein subunit
WP_043685854.1	*hpt*	74	0.51	0.272	Hypoxanthine-guanine phosphoribosyltransferase
WP_043689518.1	*hisD*	74	0.5	0.289	Histidinol dehydrogenase
WP_043691079.1	*nuoA*	74	0.49	0.322	NADH-quinone oxidoreductase subunit A
WP_086006612.1	NA	74	0.49	0.289	Trans,polycis-polyprenyl diphosphate synthase ((2Z,6E)-farnesyl diphosphate specific)
WP_043697488.1	*ychF*	76	0.76	0.271	Ribosome-binding ATPase YchF
WP_043692960.1	*prcA1*	76	0.74	0.321	Proteasome subunit alpha 1
WP_043693560.1	NA	76	0.51	0.269	MoxR family ATPase
WP_043690233.1	*glcB*	76	0.51	0.243	Malate synthase G
WP_043688221.1	*atpA*	76	0.51	0.233	ATP synthase subunit alpha
WP_043687724.1	*guaA*	76	0.5	0.291	GMP synthase
WP_043695194.1	*tsf*	76	0.5	0.269	Elongation factor Ts
WP_043700023.1	*fadI*	76	0.5	0.248	3-ketoacyl-CoA thiolase FadI
WP_043695817.1	NA	76	0.49	0.309	Putative acetyltransferase
WP_043689748.1	*argB*	76	0.49	0.291	Acetylglutamate kinase
WP_043699440.1	*pccB*	78	0.51	0.283	Propionyl-CoA carboxylase beta chain
WP_174186519.1	*pafA*	78	0.5	0.278	Pup--protein ligase
WP_043688629.1	*valS*	78	0.49	0.285	Valine--tRNA ligase
WP_043697816.1	*rplY*	78	0.47	0.275	50S ribosomal protein L25
WP_040865481.1	*clpP2*	80	0.75	0.255	ATP-dependent Clp protease proteolytic subunit 2
WP_043694738.1	NA	80	0.74	0.274	35 kDa protein
WP_039799659.1	*ilvH*	80	0.73	0.32	Acetolactate synthase small subunit
WP_051168666.1	*sir*	80	0.49	0.262	Sulfite reductase
WP_043685903.1	*lsr2*	80	0.47	0.364	Nucleoid-associated protein Lsr2
WP_043695890.1	*nrdI*	80	0.47	0.28	Protein NrdI
WP_011206964.1	*carD*	82	0.47	0.263	RNA polymerase-binding transcription factor

WP_063709467.1	*infB*	82	0.47	0.257	Translation initiation factor IF-2
WP_051169014.1	*sufC*	82	0.47	0.245	Vegetative protein
WP_083887487.1	*infC*	82	0.46	0.323	Translation initiation factor IF-3
WP_043688864.1	NA	82	0.46	0.276	tRNA-Gly
WP_083899173.1	*atpB*	84	0.73	0.302	ATP synthase subunit a
WP_043688713.1	*lepA*	84	0.47	0.284	Elongation factor 4
WP_043698931.1	*rplJ*	84	0.47	0.28	50S ribosomal protein L10
WP_043689752.1	*argG*	84	0.46	0.24	Argininosuccinate synthase
WP_043693609.1	*iscU*	84	0.45	0.298	Iron-sulfur cluster assembly scaffold protein
WP_043689275.1	*ctaE*	84	0.45	0.282	putative cytochrome c oxidase subunit 3
WP_039798939.1	*efp*	84	0.45	0.258	Elongation factor P
WP_043688095.1	NA	84	0.45	0.252	putative acyl-CoA dehydrogenase fadE25
WP_043690281.1	*uvrA*	84	0.44	0.309	UvrABC system protein A
WP_174377723.1	*narK*	84	0.44	0.291	Nitrate/nitrite transporter NarK
WP_014981573.1	NA	84	0.42	0.299	DUF742 domain-containing protein
WP_043693618.1	NA	86	0.46	0.244	Fe-S cluster assembly protein SufB
WP_043697415.1	*typA*	86	0.44	0.269	GTP-binding protein TypA/BipA
WP_086006591.1	*rplD*	86	0.44	0.257	50S ribosomal protein L4
WP_019044571.1	*crp*	86	0.43	0.305	CRP-like cAMP-activated global transcriptional regulator
WP_043693613.1	*csd*	86	0.43	0.292	putative cysteine desulfurase
WP_043687868.1	NA	86	0.43	0.252	succinate dehydrogenase hydrophobic membrane anchor subunit
WP_174315456.1	*mtrA*	88	0.44	0.307	DNA-binding response regulator
WP_043693708.1	*gapA*	88	0.44	0.23	Glyceraldehyde-3-phosphate dehydrogenase
WP_043695197.1	*rpsB*	88	0.44	0.224	30S ribosomal protein S2
WP_043698933.1	*rplA*	88	0.44	0.22	50S ribosomal protein L1
WP_043691230.1	*rbpA*	88	0.41	0.275	RNA polymerase-binding protein
WP_011210678.1	NA	88	0.38	0.282	DUF2469 domain-containing protein
WP_043687710.1	*sigD*	90	0.72	0.279	ECF RNA polymerase sigma factor
WP_174377676.1	*clpP1*	90	0.7	0.254	ATP-dependent Clp protease proteolytic subunit 1
WP_043689602.1	*rpsA*	90	0.43	0.234	30S ribosomal protein S1
WP_040864934.1	*rplM*	90	0.42	0.266	50S ribosomal protein L13
WP_043687243.1	*rplC*	90	0.42	0.244	50S ribosomal protein L3
WP_043684967.1	*sodA*	90	0.42	0.238	Superoxide dismutase
WP_067803910.1	NA	92	0.41	0.254	30S ribosomal protein S9
WP_043698018.1	*sucC*	92	0.41	0.248	Succinate--CoA ligase subunit beta
WP_043687553.1	*rpsD*	92	0.41	0.245	30S ribosomal protein S4
WP_011210681.1	*rplS*	92	0.39	0.246	50S ribosomal protein L19
WP_043687255.1	*rplV*	94	0.68	0.261	50S ribosomal protein L22
WP_043688633.1	*rplU*	94	0.41	0.249	50S ribosomal protein L21
WP_043687383.1	NA	94	0.41	0.246	30S ribosomal protein S5
WP_043687358.1	*rplE*	94	0.41	0.244	50S ribosomal protein L5

WP_043699134.1	*aceA*	94	0.4	0.232	Isocitrate lyase
WP_043687258.1	*rpsC*	94	0.38	0.243	30S ribosomal protein S3
WP_043693545.1	*inhA*	94	0.37	0.286	Enoyl-[acyl-carrier-protein] reductase
WP_043687556.1	NA	96	0.38	0.229	50S ribosomal protein L17
WP_011211115.1	*whiB1*	96	0.36	0.285	Transcriptional regulator WhiB1
WP_043693920.1	*ybiT*	98	0.36	0.288	ABC transporter ATP-binding protein
WP_039798918.1	*rpoZ*	98	0.36	0.258	RNA polymerase subunit omega
WP_043695253.1	*rpsP*	98	0.36	0.226	30S ribosomal protein S16
WP_043693616.1	NA	100	0.68	0.281	Fe-S cluster assembly protein
WP_043686525.1	NA	100	0.68	0.256	DUF3073 domain-containing protein
WP_174377715.1	*ilvC*	100	0.36	0.229	Ketol-acid reductoisomerase (NADP(+))
WP_043687550.1	*rpsM*	100	0.35	0.265	30S ribosomal protein S13
WP_043689200.1	NA	102	0.67	0.28	iron-sulfur cluster assembly accessory protein
WP_039795549.1	*rpsH*	102	0.35	0.271	30S ribosomal protein S8
WP_040782761.1	*sucD*	102	0.34	0.217	Succinate--CoA ligase subunit alpha
WP_043687250.1	*rpsS*	102	0.33	0.221	30S ribosomal protein S19
WP_003418601.1	*infA*	102	0.27	0.254	Translation initiation factor IF-1
WP_0436887380.1	*rplF*	104	0.34	0.254	50S ribosomal protein L6
WP_043695887.1	*nrdE2*	104	0.34	0.244	Ribonucleoside-diphosphate reductase subunit alpha 2
WP_043694830.1	NA	104	0.32	0.242	Alkyl hydroperoxide reductase C
WP_043688224.1	*atpD*	106	0.33	0.225	ATP synthase subunit beta
WP_011211259.1	NA	106	0.31	0.289	DUF3117 domain-containing protein
WP_014981768.1	*rpsK*	106	0.31	0.23	30S ribosomal protein S11
WP_040864865.1	*rplX*	108	0.32	0.272	50S ribosomal protein L24
WP_011210730.1	*rpmB*	108	0.29	0.209	50S ribosomal protein L28
WP_039795505.1	*rplN*	110	0.29	0.271	50S ribosomal protein L14
WP_030525248.1	*rpsG*	110	0.29	0.218	30S ribosomal protein S7
WP_043698934.1	*rplK*	112	0.64	0.24	50S ribosomal protein L11
WP_003938093.1	*rpsJ*	112	0.28	0.227	30S ribosomal protein S10
WP_040870256.1	*rpsL*	114	0.27	0.175	30S ribosomal protein S12
WP_039797323.1	*rplT*	114	0.26	0.215	50S ribosomal protein L20
WP_040864842.1	*rplP*	118	0.24	0.218	50S ribosomal protein L16
WP_011207266.1	*rplW*	118	0.23	0.181	50S ribosomal protein L23
WP_028479292.1	*groS*	120	0.24	0.282	co-chaperone GroES
WP_011211653.1	NA	122	0.61	0.206	50S ribosomal protein L33
WP_169334227.1	NA	122	0.57	0.248	glycosyltransferase

RFD: Robinson-Fould Distance; π: Nucleotide diversity
