# Supplementary material for: Whole genome sequencing reveals the genomic diversity, taxonomic classification, and evolutionary relationships of the genus Nocardia
Source: PLoS Negl Trop Dis. 2021 Aug 26;15(8):e0009665. doi: 10.1371/journal.pntd.0009665 (PMC8437295; doi:10.1371/journal.pntd.0009665)
Supplement: S4 Table — (PDF) [file pntd.0009665.s004.pdf]

S4 Table. Average Nucleotide Identity Values between type species

	N. abscessus	N. acidivorans	N. africana	N. alba	N. altamirensis	N. amamiensis	N. amikacinitolerans	N. anaemiae	N. aobensis	N. araoensis	N. arizonensis	N. arthritidis	N. asiatica	N. asteroides	N. beijingensis	N. bhagyanarayanae	N. blacklockiae	N. brasiliensis	N. brevicatena	N. caishijiensis	N. carnea	N. cerradoensis	N. concava	N. coubleae	N. crassostreae	N. cyriacigeorgica	N. donostiensis	N. elegans	N. exalbida
N. abscessus	100.00	75.06	75.28	75.86	78.83	85.42	79.37	78.10	75.47	87.81	77.76	88.82	90.30	77.29	87.53	79.36	76.59	79.61	76.81	76.57	75.42	75.31	74.91	76.34	75.10	78.08	77.24	75.58	88.69
N. acidivorans	75.06	100.00	75.31	73.80	74.49	75.02	75.34	74.85	75.30	74.99	74.76	75.15	74.94	75.05	75.04	75.58	75.92	75.03	74.03	74.52	73.31	75.13	78.97	74.40	79.72	75.30	74.46	75.35	75.06
N. africana	75.28	75.31	100.00	74.17	74.42	75.50	75.84	75.07	91.68	75.40	75.22	75.73	75.55	75.62	75.55	75.86	78.08	75.38	74.93	75.01	74.10	91.79	75.32	74.86	75.17	76.03	75.32	92.64	75.71
N. alba	75.86	73.80	74.17	100.00	75.27	76.03	76.50	75.31	74.49	75.85	75.63	76.02	75.96	83.16	76.03	76.53	75.14	75.78	74.76	82.02	74.13	74.41	73.93	82.10	73.77	76.48	75.19	74.32	76.26
N. altamirensis	78.83	74.49	74.42	75.27	100.00	79.59	78.48	78.14	74.58	79.17	77.00	79.56	79.24	76.61	79.17	78.53	75.78	82.03	75.63	76.08	74.64	74.71	74.56	75.80	74.72	77.54	76.30	74.66	79.42
N. amamiensis	85.42	75.02	75.50	76.03	79.59	100.00	79.24	78.19	75.16	84.76	77.39	85.09	84.83	77.09	84.79	79.23	76.31	79.64	76.69	76.46	75.02	75.28	74.89	76.23	75.04	78.04	77.29	75.40	85.04
N. amikacinitolerans	79.37	75.34	75.84	76.50	78.48	79.24	100.00	78.63	75.87	79.31	78.77	79.50	79.38	77.86	79.32	92.62	76.68	78.81	76.84	77.07	75.45	75.85	75.57	77.02	75.58	78.54	77.18	75.85	79.40
N. anaemiae	78.10	74.85	75.07	75.31	78.14	78.19	78.63	100.00	75.25	78.42	77.48	78.57	78.34	76.50	78.36	79.12	75.59	78.60	76.04	75.98	74.84	75.32	74.91	75.84	75.14	77.51	76.69	75.27	78.61
N. aobensis	75.47	75.30	91.68	74.49	74.58	75.16	75.87	75.25	100.00	75.44	75.36	75.64	75.49	75.49	75.58	75.90	77.97	75.25	74.87	75.01	74.34	94.93	75.31	74.73	75.20	75.89	75.06	91.39	75.70
N. araoensis	87.81	74.99	75.40	75.85	79.17	84.76	79.31	78.42	75.44	100.00	77.61	88.97	87.40	77.29	91.20	79.40	76.35	79.54	76.91	76.57	75.31	75.53	75.02	76.43	75.05	78.17	77.14	75.67	88.92
N. arizonensis	77.76	74.76	75.22	75.63	77.00	77.39	78.77	77.48	75.36	77.61	100.00	77.74	78.67	77.40	77.67	78.61	75.49	77.23	76.09	76.00	74.80	75.07	74.46	75.91	74.52	77.74	76.57	75.13	77.65
N. arthritidis	88.82	75.15	75.73	76.02	79.56	85.09	79.50	78.57	75.64	88.97	77.74	100.00	88.07	77.14	88.28	79.28	76.47	79.42	76.63	76.55	75.25	75.35	74.94	76.38	74.97	78.12	77.26	75.49	94.92
N. asiatica	90.30	74.94	75.55	75.96	79.24	84.83	79.38	78.34	75.49	87.40	78.67	88.07	100.00	77.24	87.03	79.38	76.50	79.65	76.68	76.51	75.29	75.35	75.00	76.35	75.09	78.24	77.22	75.57	88.06
N. asteroides	77.29	75.05	75.62	83.16	76.61	77.09	77.86	76.50	75.49	77.29	77.40	77.14	77.24	100.00	77.08	77.70	76.05	76.78	75.60	83.09	74.78	74.98	74.88	83.31	74.64	77.61	76.10	75.01	77.15
N. beijingensis	87.53	75.04	75.55	76.03	79.17	84.79	79.32	78.36	75.58	91.20	77.67	88.28	87.03	77.08	100.00	79.74	76.61	79.55	76.89	76.91	75.35	75.55	75.19	76.65	75.08	78.37	77.39	75.72	88.52
N. bhagyanarayanae	79.36	75.58	75.86	76.53	78.53	79.23	92.62	79.12	75.90	79.40	78.61	79.28	79.38	77.70	79.74	100.00	76.72	78.92	76.74	77.57	75.36	75.69	75.61	77.08	75.59	78.61	77.33	75.74	79.46
N. blacklockiae	76.59	75.92	78.08	75.14	75.78	76.31	76.68	75.59	77.97	76.35	75.49	76.47	76.50	76.05	76.61	76.72	100.00	76.41	75.24	75.87	74.70	78.19	76.23	75.51	76.16	76.57	75.75	78.12	76.97
N. brasiliensis	79.61	75.03	75.38	75.78	82.03	79.64	78.81	78.60	75.25	79.54	77.23	79.42	79.65	76.78	79.55	78.92	76.41	100.00	76.35	76.44	75.23	75.27	74.98	76.39	75.40	78.02	77.11	75.32	79.77
N. brevicatena	76.81	74.03	74.93	74.76	75.63	76.69	76.84	76.04	74.87	76.91	76.09	76.63	76.68	75.60	76.89	76.74	75.24	76.35	100.00	75.15	77.24	74.50	73.56	74.97	73.75	77.53	78.76	74.55	76.58
N. caishijiensis	76.57	74.52	75.01	82.02	76.08	76.46	77.07	75.98	75.01	76.57	76.00	76.55	76.51	83.09	76.91	77.57	75.87	76.44	75.15	100.00	74.44	74.52	74.27	90.88	74.17	76.71	75.71	74.48	76.20
N. carnea	75.42	73.31	74.10	74.13	74.64	75.02	75.45	74.84	74.34	75.31	74.80	75.25	75.29	74.78	75.35	75.36	74.70	75.23	77.24	74.44	100.00	73.93	72.90	74.30	72.96	76.39	77.17	74.00	75.12
N. cerradoensis	75.31	75.13	91.79	74.41	74.71	75.28	75.85	75.32	94.93	75.53	75.07	75.35	75.35	74.98	75.55	75.69	78.19	75.27	74.50	74.52	73.93	100.00	75.35	74.70	75.19	75.86	75.05	91.35	75.67
N. concava	74.91	78.97	75.32	73.93	74.56	74.89	75.57	74.91	75.31	75.02	74.46	74.94	75.00	74.88	75.19	75.61	76.23	74.98	73.56	74.27	72.90	75.35	100.00	74.68	79.33	75.61	74.44	75.61	75.42
N. coubleae	76.34	74.40	74.86	82.10	75.80	76.23	77.02	75.84	74.73	76.43	75.91	76.38	76.35	83.31	76.65	77.08	75.51	76.39	74.97	90.88	74.30	74.70	74.68	100.00	74.25	76.88	75.79	74.56	76.27
N. crassostreae	75.10	79.72	75.17	73.77	74.72	75.04	75.58	75.14	75.20	75.05	74.52	74.97	75.09	74.64	75.08	75.59	76.16	75.40	73.75	74.17	72.96	75.19	79.33	74.25	100.00	75.63	74.82	75.48	75.38
N. cyriacigeorgica	78.08	75.30	76.03	76.48	77.54	78.04	78.54	77.51	75.89	78.17	77.74	78.12	78.24	77.61	78.37	78.61	76.57	78.02	77.53	76.71	76.39	75.86	75.61	76.88	75.63	100.00	78.59	75.37	77.80
N. donostiensis	77.24	74.46	75.32	75.19	76.30	77.29	77.18	76.69	75.06	77.14	76.57	77.26	77.22	76.10	77.39	77.33	75.75	77.11	78.76	75.71	77.17	75.05	74.44	75.79	74.82	78.59	100.00	74.52	76.86
N. elegans	75.58	75.35	92.64	74.32	74.66	75.40	75.85	75.27	91.39	75.67	75.13	75.49	75.57	75.01	75.72	75.74	78.12	75.32	74.55	74.48	74.00	91.35	75.61	74.56	75.48	75.37	74.52	100.00	75.82
N. exalbida	88.69	75.06	75.71	76.26	79.42	85.04	79.40	78.61	75.70	88.92	77.65	94.92	88.06	77.15	88.52	79.46	76.97	79.77	76.58	76.20	75.12	75.67	75.42	76.27	75.38	77.80	76.86	75.82	100.00
N. farcinica	78.83	75.61	76.24	76.68	77.82	78.74	80.76	77.91	76.30	78.78	78.43	78.86	78.81	78.03	78.90	80.85	77.39	78.53	77.05	77.15	75.95	76.12	75.89	77.32	75.91	78.38	77.22	76.23	78.95
N. flavorosea	75.23	73.41	74.17	74.02	74.63	75.06	75.31	74.87	74.24	75.32	74.84	75.26	75.22	74.72	75.25	75.19	74.71	75.16	77.27	74.26	92.38	74.25	73.40	74.36	73.48	76.15	76.70	74.12	75.40
N. fluminea	75.97	73.87	74.41	89.66	75.40	75.54	76.42	75.38	74.33	75.77	75.56	75.96	75.87	83.01	76.10	76.65	75.08	75.91	74.48	81.84	73.78	74.22	74.28	82.16	74.17	76.17	74.93	74.34	76.19
N. fusca	75.73	73.48	74.34	74.21	74.62	75.26	75.51	74.98	74.30	75.62	75.06	75.59	75.58	74.95	75.69	75.55	74.94	75.33	77.63	74.44	81.53	74.32	73.49	74.38	73.66	76.45	76.77	74.32	75.81
N. gamkensis	88.74	74.99	75.70	76.18	79.52	84.98	79.50	78.50	75.60	88.93	77.68	94.90	87.98	77.17	88.48	79.45	76.91	79.76	76.51	76.34	75.06	75.56	75.31	76.37	75.35	77.84	76.76	75.71	96.65
N. grenadensis	75.87	73.71	74.68	74.44	74.97	75.87	75.95	75.29	74.69	75.92	75.41	75.81	75.89	75.22	75.93	75.90	75.20	75.59	78.06	74.75	81.29	74.76	73.79	74.78	74.06	76.75	77.07	74.65	76.08
N. harenae	77.50	74.67	75.03	75.75	76.68	77.30	77.96	76.38	74.87	77.59	76.48	77.52	77.58	77.07	77.70	78.04	76.53	77.17	75.81	75.97	75.07	74.98	75.19	76.03	74.99	77.16	76.04	75.14	77.64
N. higoensis	77.31	74.64	74.94	75.76	76.52	77.27	78.93	76.59	75.11	77.42	77.15	77.41	77.32	76.68	77.57	78.86	76.06	77.05	75.96	76.06	74.96	75.10	74.89	76.09	74.86	77.18	76.21	75.13	77.51
N. ignorata	76.22	74.30	74.75	81.89	75.90	76.01	77.01	75.79	74.65	76.23	76.06	76.22	76.24	83.06	76.55	77.51	75.50	76.25	74.92	90.96	74.24	74.61	74.64	96.80	74.51	76.66	75.27	74.66	76.36
N. inohanensis	74.94	79.19	75.14	73.94	74.53	74.76	75.42	74.87	75.23	74.92	74.42	74.90	75.01	74.74	75.10	75.52	76.14	74.99	73.72	74.22	73.04	75.25	79.74	74.23	79.48	74.93	73.89	75.19	75.10
N. jejuensis	75.19	79.44	75.25	74.28	74.76	74.99	75.40	74.88	75.32	75.14	74.74	75.13	75.11	75.04	75.34	75.62	76.34	75.45	73.73	74.23	73.07	75.25	79.11	74.28	79.13	74.77	73.69	75.28	75.38
N. jiangxiensis	74.94	74.94	76.68	73.75	74.51	74.92	75.05	75.02	76.61	75.18	74.47	75.09	74.96	74.28	75.04	75.15	77.64	74.99	74.12	73.92	73.28	76.68	75.02	73.81	75.09	74.98	74.26	76.97	75.16
N. jinanensis	75.52	73.09	74.38	74.08	74.72	75.35	75.42	74.97	74.33	75.60	74.96	75.46	75.40	74.63	75.62	75.54	74.71	75.26	77.74	74.16	81.07	74.34	73.26	74.16	73.41	76.32	76.70	74.35	75.58
N. kruczakiae	75.46	75.27	91.51	74.41	74.70	75.36	75.90	75.09	95.49	75.52	75.01	75.52	75.44	74.95	75.64	75.61	78.49	75.14	74.84	74.45	73.97	94.58	75.48	74.41	75.45	75.41	74.46	91.49	75.73
N. lijiangensis	79.13	75.19	75.60	76.07	78.29	79.17	88.24	78.44	75.48	79.07	78.31	79.09	79.09	77.24	79.33	88.55	76.46	78.60	76.31	76.62	74.87	75.46	75.42	76.54	75.52	77.73	76.32	75.40	79.27
N. mangyaensis	76.55	74.48	74.65	82.95	75.59	76.51	76.95	75.57	74.72	76.34	76.08	76.59	76.49	84.20	76.38	77.12	75.47	76.30	75.07	82.58	74.66	74.64	74.58	82.81	74.35	77.01	75.51	74.63	76.74
N. mexicana	76.27	76.01	77.84	74.56	75.66	75.96	76.29	75.81	77.72	76.06	75.19	76.17	76.08	75.43	76.24	76.36	82.63	76.17	74.59	74.84	74.01	77.74	76.31	74.83	76.42	75.87	75.14	77.82	76.42
N. mikamii	75.26	75.13	91.28	74.25	74.39	75.03	75.63	74.97	93.85	75.28	74.75	75.32	75.22	74.81	75.45	75.47	78.31	75.03	74.37	74.39	73.67	93.70	75.32	74.30	75.34	75.20	74.16	91.15	75.42
N. miyunensis	74.93	74.72	76.75	73.50	74.36	74.97	75.12	74.92	76.69	75.05	74.42	75.01	75.07	74.27	75.02	75.30	77.59	75.03	74.18	73.87	73.32	76.65	74.94	73.81	74.96	74.79	74.07	77.17	75.02
N. neocaledoniensis	76.74	74.63	75.03	82.61	76.39	76.63	77.82	76.22	75.04	76.88	76.45	76.90	76.92	85.79	77.21	78.07	76.00	76.90	75.00	82.75	74.48	75.13	75.07	82.93	75.09	77.11	75.48	75.03	77.03
N. niigatensis	75.51	79.15	75.75	74.29	74.93	75.37	75.90	75.17	75.71	75.63	74.96	75.43	75.44	75.14	75.60	75.94	76.80	75.27	74.24	74.47	73.23	75.76	84.77	74.54	79.43	75.13	74.07	75.81	75.55
N. ninae	79.60	75.03	75.05	75.73	82.85	79.57	78.81	78.83	74.97	79.53	77.07	79.50	79.55	76.73	79.64	78.91	76.13	82.96	75.87	75.94	74.81	75.02	75.07	76.02	75.11	77.44	76.58	74.99	79.68
N. niwae	88.06	75.09	75.65	76.10	79.37	84.84	79.50	78.49	75.61	88.99	77.62	89.29	87.63	77.03	88.53	79.42	76.76	79.78	76.62	76.40	75.14	75.62	75.32	76.27	75.40	77.87	76.87	75.65	89.52
N. nova	75.23	75.08	92.39	74.26	74.49	74.92	75.65	74.99	91.25	75.24	74.69	75.29	75.21	74.86	75.38	75.40	78.04	75.14	74.45	74.34	73.96	91.27	75.35	74.34	75.48	75.25	74.44	96.72	75.43
N. novocastrense	76.73	74.52	74.75	78.40	75.69	76.85	77.55	75.45	74.89	76.72	77.09	76.72	76.92	81.01	76.79	77.44	75.85	76.38	75.58	79.43	74.49	74.83	74.83	79.05	74.53	77.14	75.87	74.80	76.89
N. otitidiscaviarum	76.07	78.42	76.23	74.79	75.49	75.87	76.60	75.81	76.23	76.14	75.75	76.14	76.15	75.76	76.15	76.69	77.21	76.07	74.93	75.15	74.07	76.28	78.52	75.23	79.20	76.15	75.03	76.20	76.30
N. panacis	76.84	74.79	74.54	74.69	75.93	76.79	77.02	76.36	74.56	76.63	76.50	76.84	76.62	75.90	76.85	77.09	75.17	76.76	75.46	75.34	74.48	74.48	74.35	75.14	74.46	76.59	75.70	74.52	76.85
N. paucivorans	76.09	73.92	74.53	74.50	75.20	76.26	76.17	75.68	74.41	76.18	75.53	76.02	76.05	75.07	76.29	76.17	74.99	75.84	87.08	74.84	77.07	74.61	73.86	74.70	74.23	77.04	77.92	74.56	76.07
N. pneumoniae	85.86	75.39	75.80	76.23	79.84	91.23	79.68	78.89	75.53	85.65	77.74	85.53	85.84	77.37	85.42	79.89	76.96	80.24	76.85	76.53	75.21	75.76	75.67	76.54	75.89	78.01	77.15	75.75	85.60
N. pseudobrasiliensis	75.76	76.06	77.67	74.34	75.23	75.73	76.12	75.66	77.67	75.68	75.19	75.71	75.71	75.11	75.74	76.08	79.20	75.98	74.82	74.54	73.92	77.78	76.19	74.55	76.12	75.80	74.78	77.70	75.79
N. pseudovaccinii	77.58	74.46	74.53	75.05	77.80	77.79	78.37	90.05	74.51	77.65	76.57	77.64	77.58	75.60	77.74	78.25	75.19	77.99	75.18	74.97	73.88	74.51	74.65	75.05	74.80	76.56	75.57	74.66	77.69
N. puris	78.57	75.32	75.90	76.52	77.70	78.21	81.27	77.69	75.84	78.37	79.75	78.55	79.22	77.90	78.59	81.30	76.89	78.38	76.29	76.89	75.23	75.82	75.80	76.85	75.90	78.44	76.70	75.89	78.80
N. rhamnosiphila	75.57	73.42	74.52	74.38	74.60	75.36	75.53	74.90	74.46	75.58	75.15	75.59	75.57	75.08	75.56	75.66	74.82	75.30	77.66	74.45	81.23	74.50	73.63	74.58	73.57	76.45	76.72	74.41	75.75
N. salmonicida	75.83	73.80	74.23	88.82	75.34	75.61	76.25	75.36	74.12	75.80	75.34	75.78	75.75	82.98	75.87	76.33	74.89	75.68	74.22	81.83	73.68	74.04	74.15	82.16	74.05	75.89	74.63	74.17	75.98
N. seriolae	75.73	79.66	75.84	74.52	75.25	75.58	76.14	75.26	75.78	75.79	75.04	75.66	75.66	75.59	75.83	76.06	77.10	75.76	74.13	74.70	73.42	75.90	86.75	74.83	79.86	75.56	74.44	75.85	75.85
N. shimofusensis	77.61	74.82	75.28	75.96	76.78	77.24	79.11	76.72	75.33	77.63	77.27	77.51	77.54	76.95	77.76	78.98	76.46	77.00	76.08	76.28	75.38	75.37	75.21	76.22	75.06	77.41	76.22	75.42	77.89
N. sienata	75.89	73.72	74.81	74.58	74.85	75.72	75.88	75.16	74.75	75.87	75.30	75.77	75.89	75.23	75.85	75.92	75.13	75.46	77.92	74.66	81.31	74.71	73.71	74.81	73.82	76.61	77.45	74.74	75.99
N. speluncae	74.86	72.99	73.89	73.76	74.35	74.68	74.96	74.44	74.01	74.96	75.56	74.90	75.69	74.34	75.01	74.98	74.25	74.85	76.81	74.23	90.00	73.99	72.90	74.14	73.17	76.00	76.53	73.90	75.00
N. stercoris	74.65	74.43	75.57	74.00	73.76	74.84	75.03	73.58	75.37	74.78	74.31	74.84	74.74	75.16	74.85	75.27	76.05	74.30	74.18	74.91	73.24	75.46	74.95	74.40	74.17	75.01	73.96	75.53	74.79
N. suismassiliense	79.79	75.33	75.30	75.99	82.99	79.83	78.92	79.06	75.18	79.69	77.17	79.67	79.55	76.96	79.72	78.91	76.31	83.02	75.75	76.14	74.84	75.19	75.30	76.19	75.33	77.66	76.70	75.24	79.78
N. takedensis	77.44	74.43	74.88	75.69	76.67	77.19	78.34	76.70	74.97	77.46	81.61	77.51	77.45	76.61	77.64	78.38	76.01	77.11	75.59	75.92	74.69	74.97	74.70	75.83	74.73	77.03	75.93	75.00	77.72
N. tenerifensis	79.73	75.15	75.38	75.98	82.44	79.67	78.96	78.58	75.07	79.60	77.45	79.71	79.74	76.88	79.88	79.06	76.51	82.77	75.91	76.29	74.66	75.13	75.34	76.34	75.23	77.45	76.39	75.22	79.91
N. terpenica	76.23	76.07	77.91	74.81	75.74	76.13	76.55	75.69	77.77	76.26	75.42	76.31	76.25	75.64	76.42	76.67	79.74	76.33	74.93	75.14	74.17	77.84	76.40	74.98	76.49	76.15	75.18	77.87	76.58
N. testacea	75.69	73.53	74.80	74.45	74.85	75.65	75.85	75.03	74.61	75.73	75.48	75.70	75.70	75.22	75.82	75.97	75.11	75.55	77.80	74.52	81.45	74.72	73.76	74.70	73.81	76.69	76.96	74.71	75.95
N. thailandica	77.08	74.87	75.22	78.93	76.27	76.89	77.74	75.96	75.18	77.07	76.82	77.01	77.14	80.72	77.21	77.68	76.42	76.86	75.57	79.27	74.48	75.28	75.49	79.16	75.17	77.16	75.81	75.33	77.13
N. transvalensis	76.20	75.75	77.75	74.51	75.51	75.72	76.24	75.58	77.68	76.03	75.10	75.94	75.89	75.50	76.16	76.30	83.57	76.15	74.71	74.73	73.91	77.72	76.07	74.77	76.16	75.74	74.74	77.75	76.30
N. uniformis	74.68	77.65	74.92	73.69	74.40	74.53	75.05	74.98	74.84	74.70	74.28	74.72	74.61	74.31	74.83	75.10	75.45	74.86	73.68	73.87	72.92	74.93	77.34	74.03	78.29	74.77	73.77	74.92	74.77
N. vaccinii	75.15	74.98	76.90	73.96	74.59	75.10	75.29	74.96	76.63	75.17	74.70	75.45	75.13	74.44	75.24	75.28	77.74	75.21	74.21	74.15	73.49	76.61	75.19	74.07	75.24	75.04	74.45	76.96	75.41
N. vermiculata	74.62	74.54	80.90	73.72	74.10	74.49	74.82	74.38	81.07	74.73	74.30	74.77	74.68	74.23	74.76	74.83	76.95	74.61	74.15	73.89	74.26	81.05	74.59	73.96	74.67	74.89	74.48	80.98	74.84
N. veterana	75.51	75.42	84.71	74.42	74.80	75.45	75.76	75.34	84.27	75.53	75.11	75.47	75.55	75.23	75.71	75.84	78.23	75.65	74.47	74.78	74.19	84.33	75.64	74.69	75.68	75.64	74.72	84.25	75.64
N. vinacea	77.65	74.50	74.68	75.12	77.80	78.04	78.41	90.07	74.72	77.71	76.50	77.69	77.65	75.50	77.72	78.28	75.22	78.04	75.20	75.04	73.92	74.78	74.64	74.99	74.74	76.33	75.45	74.69	77.69
N. violaceofusca	75.28	75.22	91.72	74.31	74.55	75.38	75.66	75.23	98.95	75.37	74.89	75.28	75.21	74.85	75.48	75.58	78.17	75.17	74.37	74.48	73.76	94.78	75.50	74.32	75.40	75.29	74.45	91.39	75.41
N. vulneris	79.34	74.88	75.27	75.70	82.02	79.35	78.60	78.34	75.11	79.29	77.07	79.22	79.34	76.73	79.42	78.71	76.24	95.64	75.75	76.01	74.74	75.17	74.97	76.08	75.14	77.42	76.46	75.20	79.51
N. wallacei	[illegible]	[illegible]	[illegible]	[illegible]	[illegible]	[illegible]	[illegible]	[illegible]	[illegible]	[illegible]	[illegible]	[illegible]	[illegible]	[illegible]	[illegible]	[illegible]	[illegible]	[illegible]	[illegible]	[illegible]	[illegible]	[illegible]	[illegible]	[illegible]	[illegible]	[illegible]	[illegible]	[illegible]	[illegible]
N. xishanensis	[illegible]	[illegible]	[illegible]	[illegible]	[illegible]	[illegible]	[illegible]	[illegible]	[illegible]	[illegible]	[illegible]	[illegible]	[illegible]	[illegible]	[illegible]	[illegible]	[illegible]	[illegible]	[illegible]	[illegible]	[illegible]	[illegible]	[illegible]	[illegible]	[illegible]	[illegible]	[illegible]	[illegible]	[illegible]
N. yamanashiensis	[illegible]	[illegible]	[illegible]	[illegible]	[illegible]	[illegible]	[illegible]	[illegible]	[illegible]	[illegible]	[illegible]	[illegible]	[illegible]	[illegible]	[illegible]	[illegible]	[illegible]	[illegible]	[illegible]	[illegible]	[illegible]	[illegible]	[illegible]	[illegible]	[illegible]	[illegible]	[illegible]	[illegible]	[illegible]
N. yunnanensis	[illegible]	[illegible]	[illegible]	[illegible]	[illegible]	[illegible]	[illegible]	[illegible]	[illegible]	[illegible]	[illegible]	[illegible]	[illegible]	[illegible]	[illegible]	[illegible]	[illegible]	[illegible]	[illegible]	[illegible]	[illegible]	[illegible]	[illegible]	[illegible]	[illegible]	[illegible]	[illegible]	[illegible]	[illegible]

	N. farcinica	N. flavorosea	N. fluminea	N. fusca	N. gamkensis	N. grenadensis	N. harenae	N. higoensis	N. ignorata	N. inohanensis	N. jejuensis	N. jiangxiensis	N. jinanensis	N. kruczakiae	N. lijiangensis	N. mangyaensis	N. mexicana	N. mikamii	N. miyunensis	N. neocaledoniensis	N. niigatensis	N. ninae	N. niwae	N. nova	N. novocastrense	N. otitidiscaviarum	N. panacis	N. paucivorans	N. pneumoniae	N. pseudobrasiliensis
N. abscessus	78.83	75.23	75.97	75.73	88.74	75.87	77.50	77.31	76.22	74.94	75.19	74.94	75.52	75.46	79.13	76.55	76.27	75.26	74.93	76.74	75.51	79.60	88.06	75.23	76.73	76.07	76.84	76.09	85.86	75.76
N. acidivorans	75.61	73.41	73.87	73.48	74.99	73.71	74.67	74.64	74.30	79.19	79.44	74.94	73.09	75.27	75.19	74.48	76.01	75.13	74.72	74.63	79.15	75.03	75.09	75.08	74.52	78.42	74.79	73.92	75.39	76.06
N. africana	76.24	74.17	74.41	74.34	75.70	74.68	75.03	74.94	74.75	75.14	75.25	76.68	74.38	91.51	75.60	74.65	77.84	91.28	76.75	75.03	75.75	75.05	75.65	92.39	74.75	76.23	74.54	74.53	75.80	77.67
N. alba	76.68	74.02	89.66	74.21	76.18	74.44	75.75	75.76	81.89	73.94	74.28	73.75	74.08	74.41	76.07	82.95	74.56	74.25	73.50	82.61	74.29	75.73	76.10	74.26	78.40	74.79	74.69	74.50	76.23	74.34
N. altamirensis	77.82	74.63	75.40	74.62	79.52	74.97	76.68	76.52	75.90	74.53	74.76	74.51	74.72	74.70	78.29	75.59	75.66	74.39	74.36	76.39	74.93	82.85	79.37	74.49	75.69	75.49	75.93	75.20	79.84	75.23
N. amamiensis	78.74	75.06	75.54	75.26	84.98	75.87	77.30	77.27	76.01	74.76	74.99	74.92	75.35	75.36	79.17	76.51	75.96	75.03	74.97	76.63	75.37	79.57	84.84	74.92	76.85	75.87	76.79	76.26	91.23	75.73
N. amikacinitolerans	80.76	75.31	76.42	75.51	79.50	75.95	77.96	78.93	77.01	75.42	75.40	75.05	75.42	75.90	88.24	76.95	76.29	75.63	75.12	77.82	75.90	78.81	79.50	75.65	77.55	76.60	77.02	76.17	79.68	76.12
N. anaemiae	77.91	74.87	75.38	74.98	78.50	75.29	76.38	76.59	75.79	74.87	74.88	75.02	74.97	75.09	78.44	75.57	75.81	74.97	74.92	76.22	75.17	78.83	78.49	74.99	75.45	75.81	76.36	75.68	78.89	75.66
N. aobensis	76.30	74.24	74.33	74.30	75.60	74.69	74.87	75.11	74.65	75.23	75.32	76.61	74.33	95.49	75.48	74.72	77.72	93.85	76.69	75.04	75.71	74.97	75.61	91.25	74.89	76.23	74.56	74.41	75.53	77.67
N. araoensis	78.78	75.32	75.77	75.62	88.93	75.92	77.59	77.42	76.23	74.92	75.14	75.18	75.60	75.52	79.07	76.34	76.06	75.28	75.05	76.88	75.63	79.53	88.99	75.24	76.72	76.14	76.63	76.18	85.65	75.68
N. arizonensis	78.43	74.84	75.56	75.06	77.68	75.41	76.48	77.15	76.06	74.42	74.74	74.47	74.96	75.01	78.31	76.08	75.19	74.75	74.42	76.45	74.96	77.07	77.62	74.69	77.09	75.75	76.50	75.53	77.74	75.19
N. arthritidis	78.86	75.26	75.96	75.59	94.90	75.81	77.52	77.41	76.22	74.90	75.13	75.09	75.46	75.52	79.09	76.59	76.17	75.32	75.01	76.90	75.43	79.50	89.29	75.29	76.72	76.14	76.84	76.02	85.53	75.71
N. asiatica	78.81	75.22	75.87	75.58	87.98	75.89	77.58	77.32	76.24	75.01	75.11	74.96	75.40	75.44	79.09	76.49	76.08	75.22	75.07	76.92	75.44	79.55	87.63	75.21	76.92	76.15	76.62	76.05	85.84	75.71
N. asteroides	78.03	74.72	83.01	74.95	77.17	75.22	77.07	76.68	83.06	74.74	75.04	74.28	74.63	74.95	77.24	84.20	75.43	74.81	74.27	85.79	75.14	76.73	77.03	74.86	81.01	75.76	75.90	75.07	77.37	75.11
N. beijingensis	78.90	75.25	76.10	75.69	88.48	75.93	77.70	77.57	76.55	75.10	75.34	75.04	75.62	75.64	79.33	76.38	76.24	75.45	75.02	77.21	75.60	79.64	88.53	75.38	76.79	76.15	76.85	76.29	85.42	75.74
N. bhagyanarayanae	80.85	75.19	76.65	75.55	79.45	75.90	78.04	78.86	77.51	75.52	75.62	75.15	75.54	75.61	88.55	77.12	76.36	75.47	75.30	78.07	75.94	78.91	79.42	75.40	77.44	76.69	77.09	76.17	79.89	76.08
N. blacklockiae	77.39	74.71	75.08	74.94	76.91	75.20	76.53	76.06	75.50	76.14	76.34	77.64	74.71	78.49	76.46	75.47	82.63	78.31	77.59	76.00	76.80	76.13	76.76	78.04	75.85	77.21	75.17	74.99	76.96	79.20
N. brasiliensis	78.53	75.16	75.91	75.33	79.76	75.59	77.17	77.05	76.25	74.99	75.45	74.99	75.26	75.14	78.60	76.30	76.17	75.03	75.03	76.90	75.27	82.96	79.78	75.14	76.38	76.07	76.76	75.84	80.24	75.98
N. brevicatena	77.05	77.27	74.48	77.63	76.51	78.06	75.81	75.96	74.92	73.72	73.73	74.12	77.74	74.84	76.31	75.07	74.59	74.37	74.18	75.00	74.24	75.87	76.62	74.45	75.58	74.93	75.46	87.08	76.85	74.82
N. caishijiensis	77.15	74.26	81.84	74.44	76.34	74.75	75.97	76.06	90.96	74.22	74.23	73.92	74.16	74.45	76.62	82.58	74.84	74.39	73.87	82.75	74.47	75.94	76.40	74.34	79.43	75.15	75.34	74.84	76.53	74.54
N. carnea	75.95	92.38	73.78	81.53	75.06	81.29	75.07	74.96	74.24	73.04	73.07	73.28	81.07	73.97	74.87	74.66	74.01	73.67	73.32	74.48	73.23	74.81	75.14	73.96	74.49	74.07	74.48	77.07	75.21	73.92
N. cerradoensis	76.12	74.25	74.22	74.32	75.56	74.76	74.98	75.10	74.61	75.25	75.25	76.68	74.34	94.58	75.46	74.64	77.74	93.70	76.65	75.13	75.76	75.02	75.62	91.27	74.83	76.28	74.48	74.61	75.76	77.78
N. concava	75.89	73.40	74.28	73.49	75.31	73.79	75.19	74.89	74.64	79.74	79.11	75.02	73.26	75.48	75.42	74.58	76.31	75.32	74.94	75.07	84.77	75.07	75.32	75.35	74.83	78.52	74.35	73.86	75.67	76.19
N. coubleae	77.32	74.36	82.16	74.38	76.37	74.78	76.03	76.09	96.80	74.23	74.28	73.81	74.16	74.41	76.54	82.81	74.83	74.30	73.81	82.93	74.54	76.02	76.27	74.34	79.05	75.23	75.14	74.70	76.54	74.55
N. crassostreae	75.91	73.48	74.17	73.66	75.35	74.06	74.99	74.86	74.51	79.48	79.13	75.09	73.41	75.45	75.52	74.35	76.42	75.34	74.96	75.09	79.43	75.11	75.40	75.48	74.53	79.20	74.46	74.23	75.89	76.12
N. cyriacigeorgica	78.38	76.15	76.17	76.45	77.84	76.75	77.16	77.18	76.66	74.93	74.77	74.98	76.32	75.41	77.73	77.01	75.87	75.20	74.79	77.11	75.13	77.44	77.87	75.25	77.14	76.15	76.59	77.04	78.01	75.80
N. donostiensis	77.22	76.70	74.93	76.77	76.76	77.07	76.04	76.21	75.27	73.89	73.69	74.26	76.70	74.46	76.32	75.51	75.14	74.16	74.07	75.48	74.07	76.58	76.87	74.44	75.87	75.03	75.70	77.92	77.15	74.78
N. elegans	76.23	74.12	74.34	74.32	75.71	74.65	75.14	75.13	74.66	75.19	75.28	76.97	74.35	91.49	75.40	74.63	77.82	91.15	77.17	75.03	75.81	74.99	75.65	96.72	74.80	76.20	74.52	74.56	75.75	77.70
N. exalbida	78.95	75.40	76.19	75.81	96.65	76.08	77.64	77.51	76.36	75.10	75.38	75.16	75.58	75.73	79.27	76.74	76.42	75.42	75.02	77.03	75.55	79.68	89.52	75.43	76.89	76.30	76.85	76.07	85.60	75.79
N. farcinica	100.00	75.57	76.32	75.96	78.43	76.05	78.09	81.58	76.89	75.04	75.06	74.93	75.48	75.62	79.50	77.28	75.96	75.30	74.90	77.36	75.64	77.54	78.33	75.39	77.88	76.15	76.94	76.38	78.54	75.78
N. flavorosea	75.57	100.00	73.76	81.13	74.93	81.20	75.09	74.87	74.13	72.99	73.04	73.26	80.96	73.88	74.75	74.47	73.90	73.72	73.35	74.37	73.19	74.74	75.02	73.83	74.33	74.09	74.29	76.96	75.22	73.90
N. fluminea	76.32	73.76	100.00	74.25	76.10	74.49	75.77	75.80	82.68	74.09	74.27	73.60	74.16	74.44	76.20	83.63	74.69	74.23	73.45	82.86	74.41	75.82	76.05	74.11	78.39	74.78	74.71	74.45	76.23	74.42
N. fusca	75.96	81.13	74.25	100.00	75.57	91.56	75.60	75.41	74.32	73.35	73.35	73.50	86.74	74.37	75.26	74.58	74.15	74.05	73.56	74.60	73.66	74.86	75.51	73.97	74.72	74.42	74.51	77.36	75.47	74.19
N. gamkensis	78.43	74.93	76.10	75.57	100.00	75.88	77.55	77.42	76.31	75.04	75.27	74.91	75.48	75.64	79.08	76.65	76.39	75.41	74.92	76.91	75.51	79.60	89.33	75.37	76.84	76.19	76.83	76.06	85.49	75.73
N. grenadensis	76.05	81.20	74.49	91.56	75.88	100.00	75.38	75.19	74.22	73.33	73.25	73.47	86.20	74.17	75.32	74.92	74.17	74.07	73.57	74.58	73.54	74.84	75.53	74.01	74.99	74.35	74.99	77.26	75.65	74.04
N. harenae	78.09	75.09	75.77	75.60	77.55	75.38	100.00	76.93	75.87	74.27	74.61	74.07	75.16	74.64	77.37	76.14	75.27	74.49	74.03	76.67	74.72	76.56	77.32	74.57	76.94	75.53	75.87	75.40	77.42	74.70
N. higoensis	81.58	74.87	75.80	75.41	77.42	75.19	76.93	100.00	76.11	74.56	74.91	74.31	75.41	75.20	78.51	76.09	75.29	74.97	74.43	76.47	75.00	76.60	77.44	74.79	76.59	75.67	76.00	75.51	77.44	75.32
N. ignorata	76.89	74.13	82.68	74.32	76.31	74.22	75.87	76.11	100.00	74.14	74.23	73.62	74.07	74.32	76.35	82.63	74.71	74.24	73.75	82.98	74.43	75.97	76.12	74.18	79.09	75.09	74.99	74.59	76.37	74.44
N. inohanensis	75.04	72.99	74.09	73.35	75.04	73.33	74.27	74.56	74.14	100.00	79.06	74.80	73.28	75.38	75.31	74.43	76.24	75.14	74.76	75.03	79.77	74.96	75.28	75.37	74.56	78.64	74.34	73.96	75.70	76.03
N. jejuensis	75.06	73.04	74.27	73.35	75.27	73.25	74.61	74.91	74.23	79.06	100.00	75.15	73.55	75.56	75.63	74.59	76.35	75.37	75.12	75.17	79.55	75.39	75.51	75.42	74.82	78.37	74.39	73.95	75.80	76.26
N. jiangxiensis	74.93	73.26	73.60	73.50	74.91	73.47	74.07	74.31	73.62	74.80	75.15	100.00	74.25	77.02	75.39	74.15	77.91	77.03	89.00	74.60	75.66	75.01	75.60	77.33	74.04	76.05	74.35	74.54	75.72	77.78
N. jinanensis	75.48	80.96	74.16	86.74	75.48	86.20	75.16	75.41	74.07	73.28	73.55	74.25	100.00	73.67	74.59	74.43	73.66	73.40	72.91	73.69	73.08	74.38	74.85	73.35	74.42	73.60	74.44	76.80	74.69	73.45
N. kruczakiae	75.62	73.88	74.44	74.37	75.64	74.17	74.64	75.20	74.32	75.38	75.56	77.02	73.67	100.00	75.67	74.58	77.83	94.22	76.58	75.03	75.68	74.95	75.67	91.26	74.78	76.27	74.54	74.53	75.77	77.70
N. lijiangensis	79.50	74.75	76.20	75.26	79.08	75.32	77.37	78.51	76.35	75.31	75.63	75.39	74.59	75.67	100.00	76.66	76.44	75.71	75.26	77.74	76.09	78.98	79.52	75.57	77.14	76.69	76.81	76.18	79.86	75.98
N. mangyaensis	77.28	74.47	83.63	74.58	76.65	74.92	76.14	76.09	82.63	74.43	74.59	74.15	74.43	74.58	76.66	100.00	74.97	74.36	73.90	83.41	74.52	76.23	76.58	74.51	79.46	75.57	75.60	74.59	76.68	74.75
N. mexicana	75.96	73.90	74.69	74.15	76.39	74.17	75.27	75.29	74.71	76.24	76.35	77.91	73.66	77.83	76.44	74.97	100.00	77.59	77.48	75.50	76.51	75.94	76.36	77.77	75.31	77.12	75.15	74.81	76.62	78.97
N. mikamii	75.30	73.72	74.23	74.05	75.41	74.07	74.49	74.97	74.24	75.14	75.37	77.03	73.40	94.22	75.71	74.36	77.59	100.00	76.83	74.93	75.62	74.82	75.48	91.19	74.69	76.05	74.42	74.46	75.61	77.60
N. miyunensis	74.90	73.35	73.45	73.56	74.92	73.57	74.03	74.43	73.75	74.76	75.12	89.00	72.91	76.58	75.26	73.90	77.48	76.83	100.00	74.79	75.84	75.04	75.74	77.45	74.21	76.12	74.24	74.60	75.64	77.79
N. neocaledoniensis	77.36	74.37	82.86	74.60	76.91	74.58	76.67	76.47	82.98	75.03	75.17	74.60	73.69	75.03	77.74	83.41	75.50	74.93	74.79	100.00	75.09	76.47	77.07	74.87	79.89	75.72	75.37	74.97	77.21	75.15
N. niigatensis	75.64	73.19	74.41	73.66	75.51	73.54	74.72	75.00	74.43	79.77	79.55	75.66	73.08	75.68	76.09	74.52	76.51	75.62	75.84	75.09	100.00	75.10	75.84	75.73	75.15	78.66	74.71	74.37	75.93	76.26
N. ninae	77.54	74.74	75.82	74.86	79.60	74.84	76.56	76.60	75.97	74.96	75.39	75.01	74.38	74.95	78.98	76.23	75.94	74.82	75.04	76.47	75.10	100.00	79.84	74.90	76.29	75.98	76.34	75.64	80.51	75.74
N. niwae	78.33	75.02	76.05	75.51	89.33	75.53	77.32	77.44	76.12	75.28	75.51	75.60	74.85	75.67	79.52	76.58	76.36	75.48	75.74	77.07	75.84	79.84	100.00	75.27	76.81	76.12	76.79	76.13	85.48	75.72
N. nova	75.39	73.83	74.11	73.97	75.37	74.01	74.57	74.79	74.18	75.37	75.42	77.33	73.35	91.26	75.57	74.51	77.77	91.19	77.45	74.87	75.73	74.90	75.27	100.00	74.69	76.20	74.38	74.62	75.65	77.84
N. novocastrense	77.88	74.33	78.39	74.72	76.84	74.99	76.94	76.59	79.09	74.56	74.82	74.04	74.42	74.78	77.14	79.46	75.31	74.69	74.21	79.89	75.15	76.29	76.81	74.69	100.00	75.62	75.87	74.98	77.08	74.89
N. otitidiscaviarum	76.15	74.09	74.78	74.42	76.19	74.35	75.53	75.67	75.09	78.64	78.37	76.05	73.60	76.27	76.69	75.57	77.12	76.05	76.12	75.72	78.66	75.98	76.12	76.20	75.62	100.00	75.47	74.85	76.28	76.76
N. panacis	76.94	74.29	74.71	74.51	76.83	74.99	75.87	76.00	74.99	74.34	74.39	74.35	74.44	74.54	76.81	75.60	75.15	74.42	74.24	75.37	74.71	76.34	76.79	74.38	75.87	75.47	100.00	75.22	77.18	75.37
N. paucivorans	76.38	76.96	74.45	77.36	76.06	77.26	75.40	75.51	74.59	73.96	73.95	74.54	76.80	74.53	76.18	74.59	74.81	74.46	74.60	74.97	74.37	75.64	76.13	74.62	74.98	74.85	75.22	100.00	75.94	74.54
N. pneumoniae	78.54	75.22	76.23	75.47	85.49	75.65	77.42	77.44	76.37	75.70	75.80	75.72	74.69	75.77	79.86	76.68	76.62	75.61	75.64	77.21	75.93	80.51	85.48	75.65	77.08	76.28	77.18	75.94	100.00	75.79
N. pseudobrasiliensis	75.78	73.90	74.42	74.19	75.73	74.04	74.70	75.32	74.44	76.03	76.26	77.78	73.45	77.70	75.98	74.75	78.97	77.60	77.79	75.15	76.26	75.74	75.72	77.84	74.89	76.76	75.37	74.54	75.79	100.00
N. pseudovaccinii	76.55	73.87	74.70	74.13	77.60	74.02	75.22	75.71	74.89	74.51	74.69	75.04	73.56	74.50	78.30	75.17	75.42	74.40	75.00	75.66	74.74	78.24	77.63	74.62	75.04	75.05	75.98	74.77	77.90	74.79
N. puris	79.84	75.14	76.73	75.63	78.61	75.50	77.66	78.54	76.96	75.58	75.91	75.68	74.79	75.88	81.15	77.07	76.45	75.66	75.69	77.80	76.04	78.07	78.59	75.82	77.75	76.43	76.95	75.85	78.46	75.71
N. rhamnosiphila	75.86	81.14	74.43	93.37	75.70	91.48	75.33	75.48	74.36	73.53	73.70	74.32	85.78	74.56	75.69	74.60	74.49	74.45	74.31	74.94	74.01	75.12	75.57	74.39	74.89	74.22	74.36	77.07	75.58	73.91
N. salmonicida	76.14	73.67	90.28	73.97	75.79	73.86	75.13	75.35	82.26	74.02	74.34	74.02	73.31	74.19	76.14	83.90	74.49	74.06	74.05	82.71	74.35	75.70	75.74	74.04	78.32	74.40	74.63	73.92	75.88	73.81
N. seriolae	75.87	73.41	74.67	73.74	75.77	73.61	75.21	75.22	74.79	80.26	79.81	75.82	72.88	75.77	76.08	75.06	76.72	75.73	75.95	75.49	85.19	75.54	75.69	75.83	75.46	78.87	75.08	73.92	75.83	76.29
N. shimofusensis	82.01	75.11	75.99	75.52	77.66	75.32	77.21	88.40	76.10	75.00	75.29	75.14	74.56	75.52	79.03	76.54	75.81	75.20	75.21	76.94	75.53	76.89	77.62	75.37	77.01	75.76	76.16	75.37	77.60	75.28
N. sienata	76.16	81.18	74.49	91.75	75.93	90.75	75.56	75.78	74.66	73.70	73.89	74.34	86.08	74.82	76.06	74.90	74.72	74.73	74.41	75.13	74.18	75.29	75.78	74.73	75.08	74.57	74.72	77.20	75.83	74.34
N. speluncae	75.37	89.25	73.84	81.14	74.91	80.82	74.79	74.90	74.11	73.07	73.10	73.56	80.39	73.98	74.97	74.14	74.00	73.96	73.65	74.22	73.33	74.54	74.84	73.97	74.16	73.80	74.05	77.28	74.87	73.63
N. stercoris	75.59	73.01	74.17	73.56	74.82	73.85	74.70	74.62	74.51	74.59	74.55	74.84	73.38	75.61	74.87	74.60	76.00	75.41	74.78	74.88	75.46	74.12	74.80	75.42	75.37	75.26	74.15	73.79	74.96	75.55
N. suismassiliense	77.72	74.82	76.05	74.99	79.74	74.93	76.76	76.81	76.14	75.19	75.66	75.22	74.51	75.23	79.06	76.28	76.16	75.14	75.26	76.75	75.44	94.69	79.66	75.18	76.44	75.91	76.63	75.40	80.15	75.47
N. takedensis	78.21	74.66	75.67	74.92	77.58	74.90	76.74	77.37	75.76	74.45	74.94	74.89	74.21	75.05	78.27	75.94	75.35	74.87	75.00	76.47	75.04	76.85	77.49	74.92	76.51	75.56	76.23	75.05	77.37	74.73
N. tenerifensis	77.98	74.65	76.15	74.89	79.84	74.87	77.03	77.09	76.36	75.19	75.60	75.01	74.11	75.25	78.99	76.28	76.25	75.03	75.15	77.00	75.44	85.00	79.76	75.15	76.76	75.88	76.61	75.25	80.04	75.72
N. terpenica	76.35	74.05	74.59	74.18	76.37	74.30	75.67	75.61	74.83	76.25	76.45	77.91	73.46	77.74	76.42	75.19	79.57	77.72	78.03	75.59	76.62	75.94	76.22	77.92	75.50	77.18	75.38	74.56	76.50	79.23
N. testacea	76.24	81.23	74.54	90.78	75.79	90.15	75.63	75.74	74.56	73.72	73.88	74.52	85.72	74.70	75.87	74.82	74.72	74.63	74.53	75.16	74.20	75.27	75.84	74.63	75.07	74.45	74.65	77.26	75.76	74.15
N. thailandica	77.60	74.43	78.96	74.95	77.02	74.86	76.98	76.98	79.24	75.10	75.50	74.94	74.02	75.17	77.59	79.57	75.67	75.05	75.08	80.33	75.71	76.72	77.07	75.23	98.51	75.92	75.69	74.86	77.09	75.03
N. transvalensis	76.03	73.93	74.67	74.37	76.19	74.19	75.43	75.33	74.68	76.00	76.30	78.05	73.66	77.71	76.25	74.97	82.78	77.64	77.98	75.49	76.38	75.81	76.04	77.72	75.34	76.76	75.02	74.13	76.02	78.47
N. uniformis	74.48	72.75	73.67	73.14	74.71	73.12	73.97	74.18	73.82	77.57	77.41	74.79	72.34	74.92	75.22	74.15	75.77	74.74	74.73	74.28	77.34	74.95	74.67	74.95	74.09	80.82	73.99	73.53	74.86	75.27
N. vaccinii	75.04	73.44	73.78	73.76	75.26	73.66	74.51	74.47	73.97	74.90	75.31	88.40	73.30	76.78	75.40	74.22	77.57	76.64	87.61	74.32	75.65	74.88	75.31	76.70	74.24	75.71	74.42	74.06	75.22	77.34
N. vermiculata	74.80	74.15	73.81	74.07	74.86	73.96	74.08	74.51	73.96	74.53	74.69	76.14	73.26	81.02	74.80	74.08	76.70	80.74	76.20	74.23	74.89	74.50	74.77	81.03	74.19	75.27	73.94	73.89	74.70	76.37
N. veterana	75.63	74.16	74.43	74.15	75.62	74.37	74.94	75.26	74.61	75.47	75.63	77.05	73.70	84.36	75.81	74.61	77.92	84.50	77.21	75.05	75.77	75.20	75.42	84.32	75.22	76.18	74.83	74.19	75.54	77.39
N. vinacea	76.27	73.97	74.81	74.18	77.65	74.22	75.14	75.44	74.92	74.46	74.63	74.93	73.53	74.68	78.39	75.22	75.20	74.61	75.02	75.61	74.83	78.28	77.61	74.67	75.08	75.03	75.97	74.62	77.96	74.71
N. violaceofusca	75.38	73.72	74.22	74.05	75.33	73.96	74.52	74.85	74.27	75.42	75.36	76.89	73.40	95.21	75.53	74.49	77.88	93.90	77.11	74.97	75.57	74.91	75.26	91.30	74.61	75.96	74.44	73.99	75.30	77.22
N. vulneris	77.59	74.80	75.78	75.03	79.30	74.92	76.74	76.78	75.89	74.95	75.54	75.25	74.17	75.17	78.60	76.19	76.01	75.02	75.33	76.75	75.26	82.69	79.34	75.19	76.28	75.86	76.62	75.33	79.60	75.47
N. wallacei	[illegible]	[illegible]	[illegible]	[illegible]	[illegible]	[illegible]	[illegible]	[illegible]	[illegible]	[illegible]	[illegible]	[illegible]	[illegible]	[illegible]	[illegible]	[illegible]	[illegible]	[illegible]	[illegible]	[illegible]	[illegible]	[illegible]	[illegible]	[illegible]	[illegible]	[illegible]	[illegible]	[illegible]	[illegible]	[illegible]
N. xishanensis	[illegible]	[illegible]	[illegible]	[illegible]	[illegible]	[illegible]	[illegible]	[illegible]	[illegible]	[illegible]	[illegible]	[illegible]	[illegible]	[illegible]	[illegible]	[illegible]	[illegible]	[illegible]	[illegible]	[illegible]	[illegible]	[illegible]	[illegible]	[illegible]	[illegible]	[illegible]	[illegible]	[illegible]	[illegible]	[illegible]
N. yamanashiensis	[illegible]	[illegible]	[illegible]	[illegible]	[illegible]	[illegible]	[illegible]	[illegible]	[illegible]	[illegible]	[illegible]	[illegible]	[illegible]	[illegible]	[illegible]	[illegible]	[illegible]	[illegible]	[illegible]	[illegible]	[illegible]	[illegible]	[illegible]	[illegible]	[illegible]	[illegible]	[illegible]	[illegible]	[illegible]	[illegible]
N. yunnanensis	[illegible]	[illegible]	[illegible]	[illegible]	[illegible]	[illegible]	[illegible]	[illegible]	[illegible]	[illegible]	[illegible]	[illegible]	[illegible]	[illegible]	[illegible]	[illegible]	[illegible]	[illegible]	[illegible]	[illegible]	[illegible]	[illegible]	[illegible]	[illegible]	[illegible]	[illegible]	[illegible]	[illegible]	[illegible]	[illegible]

	N. pseudovaccinii	N. puris	N. rhamnosiphila	N. salmonicida	N. seriolae	N. shimofusensis	N. sienata	N. speluncae	N. stercoris	N. suismassiliense	N. takedensis	N. tenerifensis	N. terpenica	N. testacea	N. thailandica	N. transvalensis	N. uniformis	N. vaccinii	N. vermiculata	N. veterana	N. vinacea	N. violaceofusca	N. vulneris	N. wallacei	N. xishanensis	N. yamanashiensis	N. yunnanensis
N. abscessus	77.58	78.57	75.57	75.83	75.73	77.61	75.89	74.86	74.65	79.79	77.44	79.73	76.23	75.69	77.08	76.20	74.68	75.15	74.62	75.51	77.65	75.28	79.34	76.18	79.20	74.94	75.44
N. acidivorans	74.46	75.32	73.42	73.80	79.66	74.82	73.72	72.99	74.43	75.33	74.43	75.15	76.07	73.53	74.87	75.75	77.65	74.98	74.54	75.42	74.50	75.22	74.88	75.79	75.21	78.95	79.27
N. africana	74.53	75.90	74.52	74.23	75.84	75.28	74.81	73.89	75.57	75.30	74.88	75.38	77.91	74.80	75.22	77.75	74.92	76.90	80.90	84.71	74.68	91.72	75.27	77.79	75.56	75.07	75.66
N. alba	75.05	76.52	74.38	88.82	74.52	75.96	74.58	73.76	74.00	75.99	75.69	75.98	74.81	74.45	78.93	74.51	73.69	73.96	73.72	74.42	75.12	74.31	75.70	74.69	76.35	73.78	74.10
N. altamirensis	77.80	77.70	74.60	75.34	75.25	76.78	74.85	74.35	73.76	82.99	76.67	82.44	75.74	74.85	76.27	75.51	74.40	74.59	74.10	74.80	77.80	74.55	82.02	75.47	78.52	74.47	74.67
N. amamiensis	77.79	78.21	75.36	75.61	75.58	77.24	75.72	74.68	74.84	79.83	77.19	79.67	76.13	75.65	76.89	75.72	74.53	75.10	74.49	75.45	78.04	75.38	79.35	75.91	79.13	74.63	75.60
N. amikacinitolerans	78.37	81.27	75.53	76.25	76.14	79.11	75.88	74.96	75.03	78.92	78.34	78.96	76.55	75.85	77.74	76.24	75.05	75.29	74.82	75.76	78.41	75.66	78.60	76.24	88.13	75.33	75.84
N. anaemiae	90.05	77.69	74.90	75.36	75.26	76.72	75.16	74.44	73.58	79.06	76.70	78.58	75.69	75.03	75.96	75.58	74.98	74.96	74.38	75.34	90.07	75.23	78.34	75.46	78.75	74.67	74.70
N. aobensis	74.51	75.84	74.46	74.12	75.78	75.33	74.75	74.01	75.37	75.18	74.97	75.07	77.77	74.61	75.18	77.68	74.84	76.63	81.07	84.27	74.72	98.95	75.11	77.71	75.62	74.99	75.74
N. araoensis	77.65	78.37	75.58	75.80	75.79	77.63	75.87	74.96	74.78	79.69	77.46	79.60	76.26	75.73	77.07	76.03	74.70	75.17	74.73	75.53	77.71	75.37	79.29	76.00	79.19	74.83	75.47
N. arizonensis	76.57	79.75	75.15	75.34	75.04	77.27	75.30	75.56	74.31	77.17	81.61	77.45	75.42	75.48	76.82	75.10	74.28	74.70	74.30	75.11	76.50	74.89	77.07	75.70	78.46	74.37	75.00
N. arthritidis	77.64	78.55	75.59	75.78	75.66	77.51	75.77	74.90	74.84	79.67	77.51	79.71	76.31	75.70	77.01	75.94	74.72	75.45	74.77	75.47	77.69	75.28	79.22	76.06	79.17	74.87	75.67
N. asiatica	77.58	79.22	75.57	75.75	75.66	77.54	75.89	75.69	74.74	79.55	77.45	79.74	76.25	75.70	77.14	75.89	74.61	75.13	74.68	75.55	77.65	75.21	79.34	76.42	79.19	74.95	75.49
N. asteroides	75.60	77.90	75.08	82.98	75.59	76.95	75.23	74.34	75.16	76.96	76.61	76.88	75.64	75.22	80.72	75.50	74.31	74.44	74.23	75.23	75.50	74.85	76.73	75.53	77.50	74.78	75.65
N. beijingensis	77.74	78.59	75.56	75.87	75.83	77.76	75.85	75.01	74.85	79.72	77.64	79.88	76.42	75.82	77.21	76.16	74.83	75.24	74.76	75.71	77.72	75.48	79.42	76.18	79.44	75.04	75.54
N. bhagyanarayanae	78.25	81.30	75.66	76.33	76.06	78.98	75.92	74.98	75.27	78.91	78.38	79.06	76.67	75.97	77.68	76.30	75.10	75.28	74.83	75.84	78.28	75.58	78.71	76.33	88.33	75.44	76.14
N. blacklockiae	75.19	76.89	74.82	74.89	77.10	76.46	75.13	74.25	76.05	76.31	76.01	76.51	79.74	75.11	76.42	83.57	75.45	77.74	76.95	78.23	75.22	78.17	76.24	85.74	76.61	76.06	76.54
N. brasiliensis	77.99	78.38	75.30	75.68	75.76	77.00	75.46	74.85	74.30	83.02	77.11	82.77	76.33	75.55	76.86	76.15	74.86	75.21	74.61	75.65	78.04	75.17	95.64	76.23	78.84	74.92	75.28
N. brevicatena	75.18	76.29	77.66	74.22	74.13	76.08	77.92	76.81	74.18	75.75	75.59	75.91	74.93	77.80	75.57	74.71	73.68	74.21	74.15	74.47	75.20	74.37	75.75	74.59	76.35	73.62	74.46
N. caishijiensis	74.97	76.89	74.45	81.83	74.70	76.28	74.66	74.23	74.91	76.14	75.92	76.29	75.14	74.52	79.27	74.73	73.87	74.15	73.89	74.78	75.04	74.48	76.01	74.85	76.54	74.10	74.95
N. carnea	73.88	75.23	81.23	73.68	73.42	75.38	81.31	90.00	73.24	74.84	74.69	74.66	74.17	81.45	74.48	73.91	72.92	73.49	74.26	74.19	73.92	73.76	74.74	74.23	75.14	72.86	73.59
N. cerradoensis	74.51	75.82	74.50	74.04	75.90	75.37	74.71	73.99	75.46	75.19	74.97	75.13	77.84	74.72	75.28	77.72	74.93	76.61	81.05	84.33	74.78	94.78	75.17	77.69	75.58	75.03	75.63
N. concava	74.65	75.80	73.63	74.15	86.75	75.21	73.71	72.90	74.95	75.30	74.70	75.34	76.40	73.76	75.49	76.07	77.34	75.19	74.59	75.64	74.64	75.50	74.97	76.11	75.65	79.76	84.82
N. coubleae	75.05	76.85	74.58	82.16	74.83	76.22	74.81	74.14	74.40	76.19	75.83	76.34	74.98	74.70	79.16	74.77	74.03	74.07	73.96	74.69	74.99	74.32	76.08	74.93	76.56	74.21	74.82
N. crassostreae	74.80	75.90	73.57	74.05	79.86	75.06	73.82	73.17	74.17	75.33	74.73	75.23	76.49	73.81	75.17	76.16	78.29	75.24	74.67	75.68	74.74	75.40	75.14	76.18	75.70	79.17	79.11
N. cyriacigeorgica	76.56	78.44	76.45	75.89	75.56	77.41	76.61	76.00	75.01	77.66	77.03	77.45	76.15	76.69	77.16	75.74	74.77	75.04	74.89	75.64	76.33	75.29	77.42	75.83	78.01	74.83	75.81
N. donostiensis	75.57	76.70	76.72	74.63	74.44	76.22	77.45	76.53	73.96	76.70	75.93	76.39	75.18	76.96	75.81	74.74	73.77	74.45	74.48	74.72	75.45	74.45	76.46	74.85	76.75	73.81	74.93
N. elegans	74.66	75.89	74.41	74.17	75.85	75.42	74.74	73.90	75.53	75.24	75.00	75.22	77.87	74.71	75.33	77.75	74.92	76.96	80.98	84.25	74.69	91.39	75.20	77.68	75.65	75.05	75.71
N. exalbida	77.69	78.80	75.75	75.98	75.85	77.89	75.99	75.00	74.79	79.78	77.72	79.91	76.58	75.95	77.13	76.30	74.77	75.41	74.84	75.64	77.69	75.41	79.51	76.38	79.32	74.99	75.40
N. farcinica	76.55	79.84	75.86	76.14	75.87	82.01	76.16	75.37	75.59	77.72	78.21	77.98	76.35	76.24	77.60	76.03	74.48	75.04	74.80	75.63	76.27	75.38	77.59	76.23	79.78	75.01	76.08
N. flavorosea	73.87	75.14	81.14	73.67	73.41	75.11	81.18	89.25	73.01	74.82	74.66	74.65	74.05	81.23	74.43	73.93	72.75	73.44	74.15	74.16	73.97	73.72	74.80	74.22	75.00	72.91	73.53
N. fluminea	74.70	76.73	74.43	90.28	74.67	75.99	74.49	73.84	74.17	76.05	75.67	76.15	74.59	74.54	78.96	74.67	73.67	73.78	73.81	74.43	74.81	74.22	75.78	74.76	76.45	73.93	74.28
N. fusca	74.13	75.63	93.37	73.97	73.74	75.52	91.75	81.14	73.56	74.99	74.92	74.89	74.18	90.78	74.95	74.37	73.14	73.76	74.07	74.15	74.18	74.05	75.03	74.42	75.48	73.17	73.63
N. gamkensis	77.60	78.61	75.70	75.79	75.77	77.66	75.93	74.91	74.82	79.74	77.58	79.84	76.37	75.79	77.02	76.19	74.71	75.26	74.86	75.62	77.65	75.33	79.30	76.34	79.18	74.93	75.41
N. grenadensis	74.02	75.50	91.48	73.86	73.61	75.32	90.75	80.82	73.85	74.93	74.90	74.87	74.30	90.15	74.86	74.19	73.12	73.66	73.96	74.37	74.22	73.96	74.92	74.34	75.37	73.13	74.05
N. harenae	75.22	77.66	75.33	75.13	75.21	77.21	75.56	74.79	74.70	76.76	76.74	77.03	75.67	75.63	76.98	75.43	73.97	74.51	74.08	74.94	75.14	74.52	76.74	75.77	77.49	74.30	75.00
N. higoensis	75.71	78.54	75.48	75.35	75.22	88.40	75.78	74.90	74.62	76.81	77.37	77.09	75.61	75.74	76.98	75.33	74.18	74.47	74.51	75.26	75.44	74.85	76.78	75.57	78.66	74.57	75.14
N. ignorata	74.89	76.96	74.36	82.26	74.79	76.10	74.66	74.11	74.51	76.14	75.76	76.36	74.83	74.56	79.24	74.68	73.82	73.97	73.96	74.61	74.92	74.27	75.89	74.65	76.41	74.14	74.84
N. inohanensis	74.51	75.58	73.53	74.02	80.26	75.00	73.70	73.07	74.59	75.19	74.45	75.19	76.25	73.72	75.10	76.00	77.57	74.90	74.53	75.47	74.46	75.42	74.95	76.13	75.56	87.13	79.46
N. jejuensis	74.69	75.91	73.70	74.34	79.81	75.29	73.89	73.10	74.55	75.66	74.94	75.60	76.45	73.88	75.50	76.30	77.41	75.31	74.69	75.63	74.63	75.36	75.54	76.31	75.72	79.01	79.14
N. jiangxiensis	75.04	75.68	74.32	74.02	75.82	75.14	74.34	73.56	74.84	75.22	74.89	75.01	77.91	74.52	74.94	78.05	74.79	88.40	76.14	77.05	74.93	76.89	75.25	77.78	75.64	74.80	75.13
N. jinanensis	73.56	74.79	85.78	73.31	72.88	74.56	86.08	80.39	73.38	74.51	74.21	74.11	73.46	85.72	74.02	73.66	72.34	73.30	73.26	73.70	73.53	73.40	74.17	73.67	74.68	72.33	73.48
N. kruczakiae	74.50	75.88	74.56	74.19	75.77	75.52	74.82	73.98	75.61	75.23	75.05	75.25	77.74	74.70	75.17	77.71	74.92	76.78	81.02	84.36	74.68	95.21	75.17	77.74	75.64	75.18	75.49
N. lijiangensis	78.30	81.15	75.69	76.14	76.08	79.03	76.06	74.97	74.87	79.06	78.27	78.99	76.42	75.87	77.59	76.25	75.22	75.40	74.80	75.81	78.39	75.53	78.60	76.28	89.88	75.36	75.71
N. mangyaensis	75.17	77.07	74.60	83.90	75.06	76.54	74.90	74.14	74.60	76.28	75.94	76.28	75.19	74.82	79.57	74.97	74.15	74.22	74.08	74.61	75.22	74.49	76.19	75.04	76.77	74.14	74.82
N. mexicana	75.42	76.45	74.49	74.49	76.72	75.81	74.72	74.00	76.00	76.16	75.35	76.25	79.57	74.72	75.67	82.78	75.77	77.57	76.70	77.92	75.20	77.88	76.01	82.24	76.33	75.89	76.57
N. mikamii	74.40	75.66	74.45	74.06	75.73	75.20	74.73	73.96	75.41	75.14	74.87	75.03	77.72	74.63	75.05	77.64	74.74	76.64	80.74	84.50	74.61	93.90	75.02	77.55	75.64	74.89	75.45
N. miyunensis	75.00	75.69	74.31	74.05	75.95	75.21	74.41	73.65	74.78	75.26	75.00	75.15	78.03	74.53	75.08	77.98	74.73	87.61	76.20	77.21	75.02	77.11	75.33	77.77	75.84	74.93	75.27
N. neocaledoniensis	75.66	77.80	74.94	82.71	75.49	76.94	75.13	74.22	74.88	76.75	76.47	77.00	75.59	75.16	80.33	75.49	74.28	74.32	74.23	75.05	75.61	74.97	76.75	75.46	77.59	74.65	75.26
N. niigatensis	74.74	76.04	74.01	74.35	85.19	75.53	74.18	73.33	75.46	75.44	75.04	75.44	76.62	74.20	75.71	76.38	77.34	75.65	74.89	75.77	74.83	75.57	75.26	76.34	76.00	79.50	84.22
N. ninae	78.24	78.07	75.12	75.70	75.54	76.89	75.29	74.54	74.12	94.69	76.85	85.00	75.94	75.27	76.72	75.81	74.95	74.88	74.50	75.20	78.28	74.91	82.69	75.97	78.97	74.75	75.04
N. niwae	77.63	78.59	75.57	75.74	75.69	77.62	75.78	74.84	74.80	79.66	77.49	79.76	76.22	75.84	77.07	76.04	74.67	75.31	74.77	75.42	77.61	75.26	79.34	76.13	79.01	74.91	75.59
N. nova	74.62	75.82	74.39	74.04	75.83	75.37	74.73	73.97	75.42	75.18	74.92	75.15	77.92	74.63	75.23	77.72	74.95	76.70	81.03	84.32	74.67	91.30	75.19	77.68	75.60	74.96	75.49
N. novocastrense	75.04	77.75	74.89	78.32	75.46	77.01	75.08	74.16	75.37	76.44	76.51	76.76	75.50	75.07	98.51	75.34	74.09	74.24	74.19	75.22	75.08	74.61	76.28	75.48	77.14	74.79	75.63
N. otitidiscaviarum	75.05	76.43	74.22	74.40	78.87	75.76	74.57	73.80	75.26	75.91	75.56	75.88	77.18	74.45	75.92	76.76	80.82	75.71	75.27	76.18	75.03	75.96	75.86	76.90	76.30	78.23	78.54
N. panacis	75.98	76.95	74.36	74.63	75.08	76.16	74.72	74.05	74.15	76.63	76.23	76.61	75.38	74.65	75.69	75.02	73.99	74.42	73.94	74.83	75.97	74.44	76.62	75.05	77.01	74.12	75.20
N. paucivorans	74.77	75.85	77.07	73.92	73.92	75.37	77.20	77.28	73.79	75.40	75.05	75.25	74.56	77.26	74.86	74.13	73.53	74.06	73.89	74.19	74.62	73.99	75.33	74.42	75.64	73.37	74.17
N. pneumoniae	77.90	78.46	75.58	75.88	75.83	77.60	75.83	74.87	74.96	80.15	77.37	80.04	76.50	75.76	77.09	76.02	74.86	75.22	74.70	75.54	77.96	75.30	79.60	76.10	79.43	75.07	75.81
N. pseudobrasiliensis	74.79	75.71	73.91	73.81	76.29	75.28	74.34	73.63	75.55	75.47	74.73	75.72	79.23	74.15	75.03	78.47	75.27	77.34	76.37	77.39	74.71	77.22	75.47	78.43	75.52	75.49	76.32
N. pseudovaccinii	100.00	77.44	74.77	75.25	75.38	76.41	75.04	74.35	73.58	78.85	76.56	78.44	75.46	74.87	75.84	75.35	74.86	75.04	74.25	75.05	92.65	74.79	78.29	75.50	78.95	74.53	74.50
N. puris	77.44	100.00	75.48	76.31	75.94	78.60	75.74	75.87	75.04	77.94	77.99	78.12	76.40	75.82	77.79	76.03	74.69	75.15	74.78	75.73	76.55	75.44	77.76	76.65	80.86	75.17	76.01
N. rhamnosiphila	74.77	75.48	100.00	74.04	73.70	75.51	91.92	81.03	73.52	75.08	74.99	74.90	74.33	90.98	75.11	74.44	73.02	73.82	74.04	74.22	74.05	74.19	75.04	74.38	75.41	73.06	73.46
N. salmonicida	75.25	76.31	74.04	100.00	74.49	75.90	74.41	73.60	74.01	75.87	75.39	75.92	74.54	74.33	78.77	74.48	73.55	73.72	73.66	74.16	74.74	74.14	75.57	74.59	76.22	73.64	74.21
N. seriolae	75.38	75.94	73.70	74.49	100.00	75.29	73.90	73.14	75.34	75.41	75.02	75.59	76.81	73.92	75.64	76.29	77.41	75.42	74.78	75.93	74.45	75.54	75.22	76.51	75.75	79.81	85.39
N. shimofusensis	76.41	78.60	75.51	75.90	75.29	100.00	75.62	74.83	74.56	76.80	77.43	76.99	75.60	75.88	76.95	75.35	74.15	74.47	74.53	75.03	75.53	74.83	76.68	75.61	78.45	74.50	75.36
N. sienata	75.04	75.74	91.92	74.41	73.90	75.62	100.00	80.73	73.79	75.11	74.88	74.99	74.12	90.40	74.92	74.22	73.05	73.74	73.98	74.23	74.12	73.98	74.93	74.23	75.50	73.16	73.92
N. speluncae	74.35	75.87	81.03	73.60	73.14	74.83	80.73	100.00	72.90	74.52	74.31	74.50	73.77	81.05	74.26	73.77	72.58	73.12	74.34	73.79	73.69	73.53	74.48	74.24	74.86	72.53	73.19
N. stercoris	73.58	75.04	73.52	74.01	75.34	74.56	73.79	72.90	100.00	74.34	74.29	74.40	76.22	73.79	75.37	75.69	74.03	74.90	74.58	75.44	73.55	75.35	74.15	75.83	74.83	74.47	75.05
N. suismassiliense	78.85	77.94	75.08	75.87	75.41	76.80	75.11	74.52	74.34	100.00	76.98	85.01	76.03	75.25	76.72	75.79	74.79	74.82	74.42	75.15	78.34	74.93	82.88	75.95	78.99	74.69	75.31
N. takedensis	76.56	77.99	74.99	75.39	75.02	77.43	74.88	74.31	74.29	76.98	100.00	77.26	75.45	75.40	76.97	75.47	74.00	74.58	74.37	74.96	76.10	74.89	77.00	75.58	78.32	74.38	74.96
N. tenerifensis	78.44	78.12	74.90	75.92	75.59	76.99	74.99	74.50	74.40	85.01	77.26	100.00	76.45	75.25	77.31	76.22	74.72	75.14	74.50	75.38	78.04	75.20	82.67	76.37	79.15	75.21	75.42
N. terpenica	75.46	76.40	74.33	74.54	76.81	75.60	74.12	73.77	76.22	76.03	75.45	76.45	100.00	74.72	75.91	79.48	75.56	77.82	76.89	77.96	75.10	77.72	76.03	79.41	76.46	75.94	76.59
N. testacea	74.87	75.82	90.98	74.33	73.92	75.88	90.40	81.05	73.79	75.25	75.40	75.25	74.72	100.00	75.34	74.40	73.15	73.83	74.30	74.34	74.23	74.15	75.09	74.51	75.49	73.22	73.77
N. thailandica	75.84	77.79	75.11	78.77	75.64	76.95	74.92	74.26	75.37	76.72	76.97	77.31	75.91	75.34	100.00	75.32	74.07	74.22	74.15	75.16	75.06	74.63	76.25	75.41	77.04	74.78	75.56
N. transvalensis	75.35	76.03	74.44	74.48	76.29	75.35	74.22	73.77	75.69	75.79	75.47	76.22	79.48	74.40	75.32	100.00	75.35	77.45	76.57	77.81	74.98	77.60	75.90	82.99	76.21	75.71	76.31
N. uniformis	74.86	74.69	73.02	73.55	77.41	74.15	73.05	72.58	74.03	74.79	74.00	74.72	75.56	73.15	74.07	75.35	100.00	74.77	74.62	75.24	74.66	75.06	75.11	75.87	75.33	77.37	77.31
N. vaccinii	75.04	75.15	73.82	73.72	75.42	74.47	73.74	73.12	74.90	74.82	74.58	75.14	77.82	73.83	74.22	77.45	74.77	100.00	75.79	76.74	74.73	76.65	74.92	77.46	75.33	74.59	75.33
N. vermiculata	74.25	74.78	74.04	73.66	74.78	74.53	73.98	74.34	74.58	74.42	74.37	74.50	76.89	74.30	74.15	76.57	74.62	75.79	100.00	80.55	73.70	80.70	74.39	76.53	74.55	74.11	74.73
N. veterana	75.05	75.73	74.22	74.16	75.93	75.03	74.23	73.79	75.44	75.15	74.96	75.38	77.96	74.34	75.16	77.81	75.24	76.74	80.55	100.00	74.29	83.82	75.09	77.35	75.41	74.72	75.99
N. vinacea	92.65	76.55	74.05	74.74	74.45	75.53	74.12	73.69	73.55	78.34	76.10	78.04	75.10	74.23	75.06	74.98	74.66	74.73	73.70	74.29	100.00	75.41	78.21	75.38	78.86	74.59	74.39
N. violaceofusca	74.79	75.44	74.19	74.14	75.54	74.83	73.98	73.53	75.35	74.93	74.89	75.20	77.72	74.15	74.63	77.60	75.06	76.65	80.70	83.82	75.41	100.00	75.13	77.59	75.63	75.24	75.53
N. vulneris	78.29	77.76	75.04	75.57	75.22	76.68	74.93	74.48	74.15	82.88	77.00	82.67	76.03	75.09	76.25	75.90	75.11	74.92	74.39	75.09	78.21	75.13	100.00	76.14	78.68	74.86	75.16
N. wallacei	[illegible]	[illegible]	[illegible]	[illegible]	[illegible]	[illegible]	[illegible]	[illegible]	[illegible]	[illegible]	[illegible]	[illegible]	[illegible]	[illegible]	[illegible]	[illegible]	[illegible]	[illegible]	[illegible]	[illegible]	[illegible]	[illegible]	[illegible]	[illegible]	[illegible]	[illegible]	[illegible]
N. xishanensis	[illegible]	[illegible]	[illegible]	[illegible]	[illegible]	[illegible]	[illegible]	[illegible]	[illegible]	[illegible]	[illegible]	[illegible]	[illegible]	[illegible]	[illegible]	[illegible]	[illegible]	[illegible]	[illegible]	[illegible]	[illegible]	[illegible]	[illegible]	[illegible]	[illegible]	[illegible]	[illegible]
N. yamanashiensis	[illegible]	[illegible]	[illegible]	[illegible]	[illegible]	[illegible]	[illegible]	[illegible]	[illegible]	[illegible]	[illegible]	[illegible]	[illegible]	[illegible]	[illegible]	[illegible]	[illegible]	[illegible]	[illegible]	[illegible]	[illegible]	[illegible]	[illegible]	[illegible]	[illegible]	[illegible]	[illegible]
N. yunnanensis	[illegible]	[illegible]	[illegible]	[illegible]	[illegible]	[illegible]	[illegible]	[illegible]	[illegible]	[illegible]	[illegible]	[illegible]	[illegible]	[illegible]	[illegible]	[illegible]	[illegible]	[illegible]	[illegible]	[illegible]	[illegible]	[illegible]	[illegible]	[illegible]	[illegible]	[illegible]	[illegible]
