# Supplementary material for: Whole genome sequencing reveals the genomic diversity, taxonomic classification, and evolutionary relationships of the genus Nocardia
Source: PLoS Negl Trop Dis. 2021 Aug 26;15(8):e0009665. doi: 10.1371/journal.pntd.0009665 (PMC8437295; doi:10.1371/journal.pntd.0009665)
Supplement: S5 Table — (PDF) [file pntd.0009665.s005.pdf]

S5 Table. *in silico* DNA-DNA hybridization values between type strains

	N. abscessus	N. acidivorans	N. africana	N. alba	N. altamirensis	N. amamiensis	N. amikacinitolerans	N. anaemiae	N. aobensis	N. araoensis	N. arizonensis	N. arthritidis	N. asiatica	N. asteroides	N. beijingensis	N. bhagyanarayanae	N. blacklockiae	N. brasiliensis	N. brevicatena	N. caishijiensis	N. carnea	N. cerradoensis	N. concava	N. coubleae	N. crassostreae	N. cyriacigeorgica	N. donostiensis	N. elegans	N. exalbida	N. farcinica	N. flavorosea	N. fluminea	N. fusca	N. gamkensis	N. grenadensis	N. harenae	N. higoensis	N. ignorata	N. inohanensis	N. jejuensis	N. jiangxiensis	N. jinanensis	N. kruczakiae	N. lijiangensis	N. mangyaensis	N. mexicana	N. mikamii	N. miyunensis	N. neocaledoniensis	N. niigatensis	N. ninae	N. niwae	N. nova	N. novocastrense	N. otitidiscaviarum	N. panacis	N. paucivorans	N. pneumoniae	N. pseudobrasiliensis	N. pseudovaccinii	N. puris	N. rhamnosiphila	N. salmonicida	N. seriolae	N. shimofusensis	N. sienata	N. speluncae	N. stercoris	N. suismassiliense	N. takedensis	N. tenerifensis	N. terpenica	N. testacea	N. thailandica	N. transvalensis	N. uniformis	N. vaccinii	N. vermiculata	N. veterana	N. vinacea	N. violaceofusca	N. vulneris	N. wallacei	N. xishanensis	N. yamanashiensis	N. yunnanensis
N. abscessus	100	21.2	21.1	21.2	23.9	31.9	23.9	23.1	21.2	36.1	22.4	37.7	42.5	22.2	35.3	23.9	22.1	24.6	22.3	21.7	21.3	21.4	21.2	21.7	21	22.8	22.5	21.4	37.5	23.2	21.4	21.4	21.8	37.5	21.7	22.3	22.5	21.6	21	21.3	21.6	21.5	21.1	24.1	22	21.9	21.1	21.8	22.1	21.4	24.9	36.2	21.3	21.9	21.7	21.9	21.5	32.1	21.3	23.1	23.2	21.7	21.2	21.2	22.3	21.9	21.3	21.1	24.8	22.3	24.8	22.1	21.8	22	21.8	21.3	21.4	20.6	21.4	23.1	21.1	24.4	22.1	24	21.2	21.1
N. acidivorans	21.2	100	21.1	20.4	20.6	21.2	21.3	20.8	21.1	20.9	20.3	21	20.9	20.7	20.8	21.4	21.2	21.1	20.4	20.6	20.3	21	23.2	20.6	23.9	20.8	20.2	21.1	20.7	20.8	20.6	20.4	20.4	21.1	20.5	20.3	20.8	20.5	23.4	24	21.1	20.3	21.1	21.1	20.9	21.4	20.9	21.2	20.6	23.3	21.1	20.9	21.2	20.5	22.7	20.5	20.1	21.2	21.1	20.8	21.1	20.4	20.3	23.4	20.6	20.5	20.2	20.9	21	20.2	20.9	21.4	20.5	20.6	21.2	22.2	21.2	20.5	20.9	20.9	21	21.1	21.2	21.3	23.4	23.1
N. africana	21.1	21.1	100	20.4	20.5	21.7	21.3	21	47.4	21.4	20.8	21.2	21.1	21	21.1	21.5	22.9	21.1	21.1	20.8	20.4	47.2	21.3	20.7	21.3	21.3	20.9	51.3	21.2	21.2	20.6	20.8	20.7	21.2	20.9	20.7	21.1	20.9	21.1	21.5	22.4	20.4	46.5	21.4	21	22.9	45.9	22.8	20.8	21.3	21.2	21.1	51	20.7	21.9	20.8	20.8	21.4	22.3	20.9	21.4	21	20.6	21.3	20.9	20.8	20.3	21.3	21.3	20.6	21.4	23.2	20.7	20.7	22.9	21.2	22.2	24.7	29.4	21	47.2	21	22.7	21.3	21.1	21.1
N. alba	21.2	20.4	20.4	100	21.1	21.4	21.7	21.1	20.3	21.2	20.7	21.2	21.4	27.2	21.1	21.6	20.7	21.3	20.5	25.7	20.3	20.5	20.5	25.9	20.4	21.4	20.6	20.3	21.3	21	20.5	40.8	20.5	21.3	20.5	20.8	21.1	25.9	20.4	20.8	20.3	20.4	20.3	21.5	27.8	20.6	20.2	20.6	26.8	20.6	21.6	21	20.4	22.6	20.7	20.6	20.3	21.6	20.3	21.1	21.5	20.5	38.5	20.7	21.1	20.6	20	20.6	21.4	20.9	21.6	20.6	20.8	22.7	20.6	20.7	20.6	20.1	20.2	21	20.2	21.4	20.5	21.6	20.5	20.6
N. altamirensis	23.9	20.6	20.5	21.1	100	24.3	23.2	22.9	20.9	23.9	21.8	23.8	23.9	21.7	23.7	23.1	21.2	26.7	21.8	21.5	21	20.8	21	21.2	20.5	22.2	21.6	20.8	23.8	22.3	21.2	21.2	21.3	23.7	21.3	21.6	22	21.1	20.8	20.8	21.1	21.1	20.7	23.2	21.6	21.4	20.7	21.2	21.7	20.8	27.7	23.6	20.8	21.6	21	21.6	21.3	24.3	20.8	23	22.4	21.4	21.1	21	21.9	21.6	20.8	20.7	27.8	21.7	27.4	21.6	21.3	21.6	21.1	20.7	21.1	20.2	20.7	23.1	20.7	26.6	21.1	23.2	20.9	20.6
N. amamiensis	31.9	21.2	21.7	21.4	24.3	100	24.5	23.6	21.6	31.2	22.3	31.3	31.2	22.3	30.9	24.6	22	24.8	22.8	21.9	21.5	21.8	21.6	21.8	21.4	23.3	22.6	21.5	31.3	23.3	21.7	21.7	22.1	31.3	21.8	22.6	22.9	21.9	21.1	21.3	21.8	21.5	21.6	24.7	22.2	22.2	21.5	22	22.3	21.6	25.1	31	21.5	22	21.8	22.2	21.7	46.1	21.5	23.8	23.6	22	21.3	21.6	22.7	21.8	21.4	21.2	24.9	22.6	25.2	22.5	22.1	22	21.9	21.6	21.8	20.7	21.7	24.1	21.9	24.7	22	24.6	21.4	21.5
N. amikacinitolerans	23.9	21.3	21.3	21.7	23.2	24.5	100	23.6	21.5	23.9	23	23.7	24	22.7	23.9	50.6	22	23.7	22.3	21.9	21.5	21.5	21.6	21.9	21.4	23.1	22.3	21.2	24.1	24.9	21.6	21.8	21.5	24	21.5	22.7	23.9	22.1	21.2	21.4	21.5	21.4	21.4	38.1	22.3	22	21.3	21.5	22.6	21.6	23.7	23.7	21.4	22.4	21.9	22.2	21.6	24.4	21.3	23.9	25.7	21.7	21.6	21.5	23.8	21.8	21.3	21	23.6	23	24.1	22.2	21.9	22.4	21.7	21.3	21.5	20.6	21.1	23.8	21.4	23.6	21.9	36.1	21.5	21.5
N. anaemiae	23.1	20.8	21	21.1	22.9	23.6	23.6	100	21	23.1	21.9	23.1	23.1	21.3	23	23.5	21.3	23.2	21.6	21.5	21	21	20.9	21.5	20.9	22.3	21.8	21	23.3	22.2	21.1	21.1	21.4	23.1	21.4	21.3	21.9	21.4	20.8	21.2	21.1	21.2	21	23.5	21.6	21.6	21.1	21.6	21.4	21.2	23.6	22.9	20.8	21.3	21.3	21.5	21.3	23.5	21.2	43.6	22.5	21.5	21.1	21	22	21.8	20.9	20.6	23.4	21.9	23.4	21.6	21.4	21.4	21.3	21	21.2	20.5	20.8	43.6	21	23.3	21.4	23.3	20.8	21.1
N. aobensis	21.2	21.1	47.4	20.3	20.9	21.6	21.5	21	100	21.5	20.8	21.5	21.5	21	21.1	21.4	22.8	21.1	21.4	20.7	20.6	60.9	21.3	20.9	21.1	21.4	20.9	46.4	21.4	21.3	20.8	20.7	20.9	21.3	20.9	20.9	21	20.9	21	21.1	22.2	20.6	65.3	21.1	21.1	22.8	57.3	22.6	21	21.5	21.3	21.5	46.1	20.8	21.9	20.8	20.6	21.6	22.5	21	21.3	20.8	20.5	21.5	20.8	21.1	20.7	21.2	21.4	20.8	21.2	23	20.8	20.9	22.7	21.3	22.2	24.9	29.1	21	93.8	21.2	22.8	21.3	21.1	21.5
N. araoensis	36.1	20.9	21.4	21.2	23.9	31.2	23.9	23.1	21.5	100	22.3	37.9	35.3	22	44.8	24	22	24.3	22.5	21.8	21.4	21.5	21.3	21.7	21.1	22.9	22.2	21.4	38.1	23.3	21.5	21.4	21.5	38.1	21.7	22.5	22.7	21.6	21.1	21.3	21.6	21.6	21.5	24.2	21.9	21.9	21.2	22	21.9	21.4	24.8	38	21.3	21.9	21.7	22	21.6	31.7	21.4	23.2	23.3	21.6	21.4	21.4	22.7	21.9	21.2	21.2	24.6	22.4	24.8	22.3	21.8	22	21.8	20.9	21.2	20.7	21.5	23.2	21.4	24.3	21.9	24	21.4	21.3
N. arizonensis	22.4	20.3	20.8	20.7	21.8	22.3	23	21.9	20.8	22.3	100	22.2	23.8	22.2	22.1	23.1	21.1	22.2	21.2	21.2	20.7	21	20.5	21.2	20.3	22.1	21.1	20.5	22.2	22.5	20.8	21	21.1	22.2	20.9	21.3	21.9	21.3	20.4	20.7	20.8	20.9	20.5	23.1	21.3	21.1	20.6	20.8	21.5	20.8	22.2	22	20.6	22.1	21.1	21.5	21	22.6	20.9	22.1	24.8	21.1	20.8	20.5	21.8	21.1	22.5	20	22	26	22.3	21.2	21	21.5	20.9	20.4	20.9	20.1	20.5	22.1	20.8	22.3	21.6	23	20.5	20.5
N. arthritidis	37.7	21	21.2	21.2	23.8	31.3	23.7	23.1	21.5	37.9	22.2	100	36.2	21.9	36.8	23.7	21.8	24.1	22.1	21.7	21.4	21.5	21.2	21.7	20.8	22.8	22.2	21.2	62.2	23.3	21.2	21.3	22	62.4	21.5	22.3	22.6	21.6	20.9	21.2	21.4	21.8	21.2	23.9	21.9	21.9	20.9	21.8	22.1	21.4	24.5	38.6	21.2	21.9	21.7	22	21.4	31.6	21.3	23.1	23.2	21.8	21.2	21.2	22.4	22	21.2	20.8	24.5	22.3	24.7	22.1	21.8	21.9	21.8	20.6	21.8	20.5	21.3	23.1	21.3	24	21.8	24	21.1	21.1
N. asiatica	42.5	20.9	21.1	21.4	23.9	31.2	24	23.2	21.5	35.3	23.2	36.1	100	22.2	34.7	24	22.1	24.4	22.2	21.7	21.6	21.6	21.4	21.7	21	23	22.3	21.5	36.1	23.3	21.5	21.4	21.6	36.1	21.5	22.5	22.7	21.6	20.9	21.1	21.7	21.4	21.3	24.1	22	22	21	21.7	22.1	21.4	24.7	35	21.5	22	21.8	22.1	21.5	31.8	21.4	23.2	24.1	21.7	21.3	21.4	22.6	21.7	22.7	21.3	24.7	22.4	24.8	22.2	21.8	22.2	21.9	21.1	21.3	20.5	21.5	23.4	21.3	24.5	22.4	23.9	21.3	21.2
N. asteroides	22.2	20.7	21	27.2	21.7	22.3	22.7	21.3	21	22	22.2	21.9	22.2	100	22.1	22.4	21.5	22.1	21.6	27.2	20.8	20.9	20.9	27.5	20.8	22.2	21.3	21	22	22.2	20.6	27.9	20.7	22	21.2	21.6	21.9	27.4	20.8	21	21	20.7	20.9	22.5	29.6	21.2	20.7	20.9	31.7	21.2	22.3	22	20.9	24.6	21.4	21.1	20.8	22.3	20.9	21.5	22.5	21	27.6	21.1	21.9	21	20.6	21	22.2	21.6	22.2	21.4	21	24.3	21.2	20.9	20.8	20.5	21	21.7	20.8	22	21.3	22.4	20.9	20.9
N. beijingensis	35.3	20.8	21.1	21.1	23.7	30.9	23.9	23	21.1	44.8	22.1	36.8	34.7	22.1	100	24	22	24.2	22.3	21.8	21	21.3	21.2	21.6	21	22.9	22.3	21.1	37.2	23.2	21	21.2	21.6	36.9	21.4	22.1	22.4	21.7	21.1	21.3	21.3	21.4	21.2	24	21.9	21.6	20.9	21.6	22	21.2	24.5	36.9	21.1	21.8	21.7	21.9	21.4	31.1	21.5	23.1	23.2	21.7	21.1	21.2	22.5	21.8	20.9	21.1	24.4	22.4	24.6	22.3	21.6	21.9	21.7	21.2	21.4	20.6	21.2	23	21.1	24.1	21.9	24.1	21.3	21.2
N. bhagyanarayanae	23.9	21.4	21.5	21.6	23.1	24.6	50.6	23.5	21.4	24	23.1	23.7	24	22.4	24	100	22	23.6	22.5	22.4	21.5	21.5	21.4	22	21.4	23	22.3	21.4	23.8	24.8	21.6	21.9	21.6	23.8	21.8	22.6	23.8	22.5	21.2	21.5	21.5	21.6	21.3	38.6	22.2	21.9	21.2	21.5	22.8	21.7	23.8	23.6	21.3	22.4	22	22.1	21.5	24.5	21.4	23.6	25.7	21.9	21.5	21.7	23.6	22	21.4	21.2	23.7	23.2	24	22.3	22	22.4	21.9	21.5	21.5	20.9	21.3	23.6	21.3	23.6	21.8	36.5	21.4	21.5
N. blacklockiae	22.1	21.2	22.9	20.7	21.2	22	22	21.3	22.8	22	21.1	21.8	22.1	21.5	22	22	100	21.7	21.6	21	20.8	22.9	21.8	20.9	21.5	21.7	21.2	22.8	22	22	20.9	20.6	21.2	21.9	21.1	21.4	21.6	20.8	21.5	21.7	22.9	20.6	23.3	22	21.1	27.6	23.2	23.2	21.3	21.9	21.7	21.8	22.9	21.3	22.3	21.1	21.3	22.2	23.3	21.4	21.8	21.3	20.6	21.9	21.5	21.1	20.7	21.8	21.7	21.2	22	24.9	21.1	21.3	28.5	21.4	22.7	21.7	22.9	21.2	22.9	21.6	31.8	22.1	21.7	21.8
N. brasiliensis	24.6	21.1	21.1	21.3	26.7	24.8	23.7	23.2	21.1	24.3	22.2	24.1	24.4	22.1	24.2	23.6	21.7	100	22.2	21.7	21.3	21.4	21	21.9	20.9	22.7	22	21.2	24.2	22.8	21.4	21.6	21.6	24.1	21.5	22.1	22.3	21.8	20.9	21.3	21.4	21.2	21.2	23.5	22	21.6	21.2	21.5	22.1	21.4	27.6	24.2	21.3	21.8	21.4	21.7	21.3	24.9	21.4	23.1	22.8	21.5	21.2	21.3	22.4	21.6	21.3	21	27.6	22.2	27.4	21.8	21.8	21.8	21.6	21.1	21.4	20.6	21.2	23.3	21	65.6	21.4	23.4	21	21.1
N. brevicatena	22.3	20.4	21.1	20.5	21.8	22.8	22.3	21.6	21.4	22.5	21.2	22.1	22.2	21.6	22.3	22.5	21.6	22.2	100	21.2	22.4	21.3	20.8	21.2	20.5	22.8	23.5	21.2	22.5	22.2	22.5	20.8	22.8	22.3	22.7	21.6	22.1	21.2	20.4	20.4	21.2	22.6	21.4	22.5	21.4	21.5	21	21.4	21.4	21	22.2	22.6	21.6	21.3	21.5	21.1	34.1	22.7	21.2	21.9	22.1	23	20.6	20.6	21.8	23	22.3	20.9	21.9	21.3	22.1	21.8	23	21.2	21.6	21.2	21.3	20.7	21	22	21	22.2	21.4	22.2	20.7	20.7
N. caishijiensis	21.7	20.6	20.8	25.7	21.5	21.9	21.9	21.5	20.7	21.8	21.2	21.7	21.7	27.2	21.8	22.4	21	21.7	21.2	100	21	21	20.7	43.8	20.7	21.8	21	20.9	21.7	21.6	20.9	26.2	20.7	21.6	20.7	21.1	21.7	44	20.7	20.8	20.8	20.2	20.8	21.9	27.1	20.9	20.7	21	27.3	21	21.9	21.6	21	23.1	21	20.7	20.6	22	20.4	21.6	21.9	20.8	26.1	21	21.6	21	20.7	20.9	21.9	21.1	22	21.3	20.9	23.2	20.8	20.7	20.7	20.4	20.8	21.6	20.7	21.8	20.8	21.9	20.9	20.7
N. carnea	21.3	20.3	20.4	20.3	21	21.5	21.5	21	20.6	21.4	20.7	21.4	21.6	20.8	21	21.5	20.8	21.3	22.4	21	100	20.5	20.1	20.9	20.3	21.7	22	20.7	21.4	21.3	49.6	20.5	26	21.2	25.5	21.1	21.1	21	20.1	20.3	20.6	25.5	20.6	21.4	21	20.8	20.3	20.8	20.7	20.4	21.3	21.4	20.8	20.5	20.7	20.7	22.2	21.5	20.4	20.9	21.4	25.8	20.2	20.4	21.4	25.6	41.7	20.4	21.1	21	21.2	20.9	25.8	20.5	20.6	20.5	20.4	20.7	20.9	21	20.4	21.1	20.8	21.3	20.5	20.3
N. cerradoensis	21.4	21	47.2	20.5	20.8	21.8	21.5	21	60.9	21.5	21	21.5	21.6	20.9	21.3	21.5	22.9	21.4	21.3	21	20.5	100	21.4	20.9	21.2	21.4	20.8	46.2	21.3	21.3	20.8	20.6	20.8	21.4	21.2	20.9	21	21	21.1	21.2	22.6	20.3	59.8	21.3	21	23	56.6	22.8	20.8	21.5	21.4	21.4	46.1	21	21.9	20.8	20.8	21.7	22.7	20.9	21.2	21	20.5	21.4	21	21.2	20.8	21.3	21.5	21.1	21.3	23.2	20.8	21	22.8	21.3	22.4	25	29.2	21.2	61.3	21.3	22.9	21.3	21.2	21.5
N. concava	21.2	23.2	21.3	20.5	21	21.6	21.6	20.9	21.3	21.3	20.5	21.2	21.4	20.9	21.2	21.4	21.8	21	20.8	20.7	20.1	21.4	100	20.8	23.7	21.1	20.4	21.2	21.2	21.1	20.3	20.5	20.8	21.2	20.9	20.8	21.1	20.8	23.8	23.7	21.3	20.4	21.3	21.3	21	21.8	21.3	21.5	20.8	30.2	21.1	21.1	21.2	21	22.8	20.5	20.3	21.4	21.2	20.8	21.3	20.7	20.4	32.7	21	21	19.9	21.4	20.9	20.7	21.2	21.8	21.1	21	21.7	22.3	21.2	20.7	21.3	21.1	21.3	21	21.6	21.4	24.3	30.3
N. coubleae	21.7	20.6	20.7	25.9	21.2	21.8	21.9	21.5	20.9	21.7	21.2	21.7	21.7	27.5	21.6	22	20.9	21.9	21.2	43.8	20.9	20.9	20.8	100	20.5	21.8	21.1	20.9	21.7	21.5	20.9	26.7	20.8	21.6	20.9	20.9	21.7	74.8	20.5	20.7	20.6	20.2	20.8	21.7	27.6	20.9	20.6	20.7	27.4	20.8	21.8	21.4	20.9	23	20.9	20.6	20.5	22	20.4	21.5	21.9	20.9	26.6	20.7	21.5	20.8	20.7	20.9	21.6	21.1	21.9	21.1	20.8	23.1	20.8	20.8	20.6	20.3	20.9	21.6	20.8	21.8	20.8	21.8	20.7	20.8
N. crassostreae	21	23.9	21.3	20.4	20.5	21.4	21.4	20.9	21.1	21.1	20.3	20.8	21	20.8	21	21.4	21.5	20.9	20.5	20.7	20.3	21.2	23.7	20.5	100	20.9	20.3	21.3	21	20.8	20.3	20.4	20.5	21	20.5	20.4	20.8	20.4	23.8	23.9	21.2	20.2	21.1	21.4	20.8	21.5	21	21.3	20.8	23.9	21	21	21.2	20.9	23.3	20.5	20.3	21.3	21.3	20.8	21.3	20.7	20.1	23.8	20.8	20.5	20.4	20.9	20.8	20.6	21.1	21.7	20.8	20.9	21.5	22.7	21.1	20.5	20.9	20.8	21.1	21	21.4	21.3	24	23.5
N. cyriacigeorgica	22.8	20.8	21.3	21.4	22.2	23.3	23.1	22.3	21.4	22.9	22.1	22.8	23	22.2	22.9	23	21.7	22.7	22.8	21.8	21.7	21.4	21.1	21.8	20.9	100	23	21.4	22.8	22.7	21.7	21.5	21.9	22.9	22	21.8	22.5	21.8	20.8	21.2	21.3	21.6	21.3	23	22.1	21.8	21	21.7	22.2	21.3	22.8	22.7	21.4	21.9	21.6	21.7	22	23.1	21.1	22.4	23.1	21.9	21.5	21.1	22.3	22.2	21.2	21	22.8	22.1	22.7	22	22.2	22	21.6	21	21.3	20.7	21.1	22.4	21.3	22.7	21.6	23.1	21.1	21.1
N. donostiensis	22.5	20.2	20.9	20.6	21.6	22.6	22.3	21.8	20.9	22.2	21.1	22.2	22.3	21.3	22.3	22.3	21.2	22	23.5	21	22	20.8	20.4	21.1	20.3	23	100	20.8	22.2	22	21.9	20.9	21.9	22.1	21.9	21.1	21.5	21.1	20.3	20.3	21	21.9	20.7	22.3	21.1	21.4	20.6	20.8	21.3	20.6	22.1	22.3	20.9	21	21	21	22.4	22.6	20.9	21.7	21.8	22.1	20.5	20.3	21.3	22.6	21.7	20.2	22.1	21.4	22.1	21.3	22.1	21	21	20.5	20.8	20.4	20.6	21.8	20.8	21.9	20.8	22.2	20.3	20.3
N. elegans	21.4	21.1	51.3	20.3	20.8	21.5	21.2	21	46.4	21.4	20.5	21.2	21.5	21	21.1	21.4	22.8	21.2	21.2	20.9	20.7	46.2	21.2	20.9	21.3	21.4	20.8	100	21.3	21.4	20.7	20.4	20.9	21.2	21.1	20.8	21	20.9	20.8	21.3	22.6	20.2	46	21.3	21	22.9	45.6	23.1	21	21.4	21.5	21.2	**75.1**	20.9	21.7	20.9	20.7	21.4	22.4	20.9	21.4	21	20.3	21.3	20.8	20.9	20.5	21.2	21.3	20.7	21.4	23.2	20.8	21	22.8	21	22.4	24.9	28.8	20.9	46.1	21.3	22.8	21	21.1	21.2
N. exalbida	37.5	20.7	21.2	21.3	23.8	31.3	24.1	23.3	21.4	38.1	22.2	62.2	36.1	22	37.2	23.8	22	24.2	22.5	21.7	21.4	21.3	21.2	21.7	21	22.8	22.2	21.3	100	23.2	21.3	21.5	21.7	73.7	21.7	22.2	22.6	21.8	21	21.3	21.6	21.5	21.2	23.9	21.9	22	20.9	21.7	22.2	21.3	24.7	39.1	21.4	21.8	21.7	22	21.4	31.7	21.4	23.1	23.3	21.9	21.2	21.3	22.4	21.9	21.1	20.7	24.7	22.4	24.7	22.1	21.8	21.9	22	20.9	21.6	20.6	21.4	23.2	21.3	24.3	22	24	21.1	21.1
N. farcinica	23.2	20.8	21.2	21	22.3	23.3	24.9	22.2	21.3	23.3	22.5	23.3	23.3	22.2	23.2	24.8	22	22.8	22.2	21.6	21.3	21.3	21.1	21.5	20.8	22.7	22	21.4	23.2	100	21.5	21.4	21.5	23.1	21.2	22.2	26.1	21.6	21.1	21.2	21	20.9	21.2	25	21.7	21.7	21.1	21.4	22.1	21.4	22.8	22.9	21.3	22	21.5	21.6	21.6	23.3	21.3	22.3	24.4	21.5	21	21.2	25.9	21.8	21	20.8	22.8	22.8	23	21.9	21.6	22.2	21.5	20.8	21.1	20.4	21.4	22.2	21.2	22.7	21.6	24.5	21.2	21.2
N. flavorosea	21.4	20.6	20.6	20.5	21.2	21.7	21.6	21.1	20.8	21.5	20.8	21.2	21.5	20.6	21	21.6	20.9	21.4	22.5	20.9	49.6	20.8	20.3	20.9	20.3	21.7	21.9	20.7	21.3	21.5	100	20.6	25.7	21.2	25.6	21.3	21.4	20.9	20.2	20.6	20.6	25.3	21.1	21.5	21.1	20.5	20.6	20.5	21	20.3	21.4	21.3	20.5	20.9	20.7	21	22.1	21.6	20.4	21	21.5	25.8	20.2	20.5	21.2	25.5	39.9	20.5	21.1	21	21.2	21	25.8	20.9	20.8	20.7	20.6	20.9	20.7	21.2	20.7	21.2	20.9	21.6	20.5	20.3
N. fluminea	21.4	20.4	20.8	40.8	21.2	21.7	21.8	21.1	20.7	21.4	21	21.3	21.4	27.9	21.2	21.9	20.6	21.6	20.8	26.2	20.5	20.6	20.5	26.7	20.4	21.5	20.9	20.4	21.5	21.4	20.6	100	20.7	21.3	20.7	21	21.2	26.8	20.7	20.8	20.7	20.5	20.5	21.7	28.9	20.7	20.2	20.8	27.3	20.6	21.6	21.2	20.4	22.8	20.8	20.7	20.3	21.5	20.6	21.1	21.7	21	41.8	20.7	21.2	20.9	20.3	20.5	21.6	21.1	21.8	21	20.9	22.9	20.8	20.8	20.5	20.4	20.7	21.4	20.5	21.4	20.6	21.8	20.6	20.7
N. fusca	21.8	20.4	20.7	20.5	21.3	22.1	21.5	21.4	20.9	21.5	21.1	22	21.6	20.7	21.6	21.6	21.2	21.6	22.8	20.7	26	20.8	20.8	20.8	20.5	21.9	21.9	20.9	21.7	21.5	25.7	20.7	100	21.7	47	21.3	21.2	20.7	20.3	20.3	20.5	33.4	20.9	21.9	20.7	20.9	20.5	20.5	20.9	20.5	21.8	21.5	20.8	20.9	21	20.7	22	21.7	20.7	21.4	21.8	55.5	20.3	20.5	21.4	47.6	25.5	20.6	21.3	20.9	21.4	21.4	43.6	21	21	20.8	20.3	20.6	20.6	21.6	20.7	21.5	21	21.7	20.4	20.4
N. gamkensis	37.5	21.1	21.2	21.3	23.7	31.3	24	23.1	21.3	38.1	22.2	62.4	36.1	22	36.9	23.8	21.9	24.1	22.3	21.6	21.2	21.4	21.2	21.6	21	22.9	22.1	21.2	73.7	23.1	21.2	21.3	21.7	100	21.6	22.2	22.6	21.6	21	21.2	21.5	21.4	21.2	23.9	21.9	21.9	20.9	21.8	22.1	21.6	24.6	39	21.1	21.8	21.8	21.9	21.5	31.6	21.3	23.2	23.2	21.6	21	21.2	22.4	21.8	21	20.7	24.6	22.4	24.7	22	21.7	21.9	21.8	21	21.4	20.5	21.3	23	21.3	24.1	21.7	24	21.1	21.2
N. grenadensis	21.7	20.5	20.9	20.5	21.3	21.8	21.5	21.4	20.9	21.7	20.9	21.5	21.5	21.2	21.4	21.8	21.1	21.5	22.7	20.7	25.5	21.2	20.9	20.9	20.5	22	21.9	21.1	21.7	21.2	25.6	20.7	47	21.6	100	21.6	21.6	20.9	20.2	20.6	20.7	32.3	21.1	21.9	20.9	21.2	20.7	20.7	21.1	20.4	21.4	21.8	21.1	21	20.9	20.6	22.1	21.7	20.6	21.4	21.6	47.4	20.3	20.7	21.5	44	25.3	20.6	21.2	20.9	21.4	21.3	42.1	21.4	21.2	20.9	20.7	20.9	21	21.4	20.7	21.4	20.9	22	20.2	20.4
N. harenae	22.3	20.3	20.7	20.8	21.6	22.6	22.7	21.3	20.9	22.5	21.3	22.3	22.5	21.6	22.1	22.6	21.4	22.1	21.6	21.1	21.1	20.9	20.8	20.9	20.4	21.8	21.1	20.8	22.2	22.2	21.3	21	21.3	22.2	21.6	100	21.9	20.9	20.2	20.6	20.9	21	20.9	22.6	21.3	21.2	20.8	21	21.6	20.8	22.1	22.2	20.9	21.7	20.9	21	21.4	22.6	20.5	21.5	22.5	21.4	20.5	20.6	21.9	21.3	20.9	20.6	22	21.5	22.2	21.4	21.5	21.7	21.1	20.4	21	20.2	20.9	21.6	20.9	22.2	21.3	22.5	20.7	20.5
N. higoensis	22.5	20.8	21.1	21.1	22	22.9	23.9	21.9	21	22.7	21.9	22.6	22.7	21.9	22.4	23.8	21.6	22.3	22.1	21.7	21.1	21	21.1	21.7	20.8	22.5	21.5	21	22.6	26.1	21.4	21.2	21.2	22.6	21.6	21.9	100	21.6	21	20.9	21.2	21	21.2	23.8	21.7	21.6	20.8	21.1	21.8	21.2	22.6	22.5	21.1	21.7	21.6	21.6	21.3	23	21.3	21.9	23.4	21.4	21.1	21.1	36.2	21.5	21	20.7	22.6	22.2	22.7	21.7	21.5	21.8	21.4	21.1	21.2	20.3	21.1	21.8	20.9	22.3	21.4	23.7	21.1	21.1
N. ignorata	21.6	20.5	20.9	25.9	21.1	21.9	22.1	21.4	20.9	21.6	21.3	21.6	21.6	27.4	21.7	22.5	20.8	21.8	21.2	44	21	21	20.8	**74.8**	20.4	21.8	21.1	20.9	21.8	21.6	20.9	26.8	20.7	21.6	20.9	20.9	21.6	100	20.6	20.6	20.6	20.4	20.9	21.8	27.5	20.8	20.7	20.8	27.5	20.8	21.9	21.4	21	23.1	20.9	20.7	20.6	21.9	20.4	21.4	22.1	21.1	26.8	20.7	21.5	21	20.5	20.9	21.6	21.1	21.8	21.1	20.9	23.1	20.8	20.7	20.6	20.5	21	21.6	20.9	21.7	20.7	21.7	20.7	20.9
N. inohanensis	21	23.4	21.1	20.4	20.8	21.1	21.2	20.8	21	21.1	20.4	20.9	20.9	20.8	21.1	21.2	21.5	20.9	20.4	20.7	20.1	21.1	23.8	20.5	23.8	20.8	20.3	20.8	21	21.1	20.2	20.7	20.3	21	20.2	20.2	21	20.6	100	23.8	21.2	20.4	21	21.2	20.8	21.6	20.7	21.1	20.8	24.1	21	20.9	20.9	20.8	22.9	20.6	20.3	21.1	21.2	20.8	21.1	20.4	20.4	24.1	20.9	20.3	20	20.9	21	20.4	21.1	21.6	20.3	20.8	21.5	22.3	21.1	20.6	21	20.8	20.9	21	21.4	21.1	34.7	23.7
N. jejuensis	21.3	24	21.5	20.8	20.8	21.3	21.4	21.2	21.1	21.3	20.7	21.2	21.1	21	21.3	21.5	21.7	21.3	20.4	20.8	20.3	21.2	23.7	20.7	23.9	21.2	20.3	21.3	21.3	21.2	20.6	20.8	20.3	21.2	20.6	20.6	20.9	20.6	23.8	100	21.6	20.3	21.3	21.5	21	21.5	21.1	21.5	21	24	21.4	21.2	21.4	20.9	23.1	20.7	20.3	21.4	21.5	20.9	21.3	20.4	20.6	23.8	21	20.5	20.5	21	21.3	20.9	21.5	22	20.8	21	21.6	22.5	21.4	20.7	21.1	21.1	21	21.2	21.3	21.4	23.8	23.7
N. jiangxiensis	21.6	21.1	22.4	20.3	21.1	21.8	21.5	21.1	22.2	21.6	20.8	21.4	21.7	21	21.3	21.5	22.9	21.4	21.2	20.8	20.6	22.6	21.3	20.6	21.2	21.3	21	22.6	21.6	21	20.6	20.7	20.5	21.5	20.7	20.9	21.2	20.6	21.2	21.6	100	20	22.3	21.6	20.8	22.9	22.3	40.6	20.8	21.5	21.4	21.5	22.5	20.9	21.7	21	20.8	21.5	22.5	21.4	21.3	20.6	20.2	21.3	21.1	20.8	20.4	21	21.2	20.8	21.4	23.1	20.4	20.9	22.9	21.3	38.2	21.3	22.1	21.4	22.2	21.3	22.7	21.7	21.3	21.3
N. jinanensis	21.5	20.3	20.4	20.4	21.1	21.5	21.4	21.2	20.6	21.6	20.9	21.8	21.4	20.7	15.5	15.2	15	15.1	17.4	15.1	24	20.3	13.9	15.2	14.2	16.8	16.9	15.1	15.5	16.3	23.4	14.8	33.4	15.4	33.5	15.6	15.6	15.1	14.2	14	14.5	100	15.2	15.2	20.8	14.5	15.1	14.2	15.3	14.2	14.6	15.4	14.8	15.2	20.5	20.4	21.9	21.5	20.6	21	21.2	33.1	20	20.3	21.3	32.8	25	19.8	21.2	20.5	21	20.9	32.5	20.4	20.7	20.6	20.2	20.1	20.6	21	20.5	21.1	20.6	21.4	20.1	19.9
N. kruczakiae	21.1	21.1	46.5	20.3	20.7	21.6	21.4	21	65.3	21.5	20.5	21.2	21.3	20.9	21.2	21.3	23.3	21.2	21.4	20.8	20.6	59.8	21.3	20.8	21.1	21.3	20.7	46	21.2	21.2	21.1	20.5	20.9	21.2	21.1	20.9	21.2	20.9	21	21.3	22.3	20.1	100	21.2	20.9	22.9	58	22.6	20.8	21.4	21.2	21.2	46.3	20.8	21.8	20.9	20.5	21.5	22.4	21	21.3	20.8	20.3	21.2	20.9	21.2	20.5	21.2	21.3	20.6	21.4	23.2	20.7	21	22.8	21.3	22.2	24.9	28.9	21.1	64.2	21.2	22.6	21.3	21.1	21.3
N. lijiangensis	24.1	21.1	21.4	21.5	23.2	24.7	38.1	23.5	21.1	24.2	23.1	23.9	24.1	22.5	24	38.6	22	23.5	22.5	21.9	21.4	21.3	21.3	21.7	21.4	23	22.3	21.3	23.9	25	21.5	21.7	21.9	23.9	21.9	22.6	23.8	21.8	21.2	21.5	21.6	21.3	21.2	100	22.2	22.1	21	21.6	22.5	21.6	23.8	23.9	21.3	22.3	21.9	22.2	21.8	24.6	21.2	23.5	25.6	21.8	21.4	21.5	23.8	22.1	21.1	21.4	23.9	23	23.9	22.2	22.1	22.4	21.7	21.6	21.6	20.7	21.2	23.7	21.1	23.5	21.9	40.1	21.4	21.5
N. mangyaensis	22	20.9	21	27.8	21.6	22.2	22.3	21.6	21.1	21.9	21.3	21.9	22	29.6	21.9	22.2	21.1	22	21.4	27.1	21	21	21	27.6	20.8	22.1	21.1	21	21.9	21.7	21.1	28.9	20.7	21.9	20.9	21.3	21.7	27.5	20.8	21	20.8	20.8	20.9	22.2	100	21.1	20.8	21.1	28.8	21.1	22	21.6	21.3	23.7	21.5	21	20.6	22.2	20.7	21.4	22	21	29	21.1	21.6	21	20.9	20.9	21.9	21.5	22.1	21.3	21	23.7	21	21.4	20.7	20.6	21	21.7	21	22	21.1	22.2	21.1	21.1
N. mexicana	21.9	21.4	22.9	20.6	21.4	22.2	22	21.6	22.8	21.9	21.1	21.9	22	21.2	21.6	21.9	27.6	21.6	21.5	20.9	20.8	23	21.8	20.9	21.5	21.8	21.4	22.9	22	21.7	20.5	20.7	20.9	21.9	21.2	21.2	21.6	20.8	21.6	21.5	22.9	20.8	22.9	22.1	21.1	100	22.8	23.1	21.1	21.9	21.9	21.7	23	20.9	22.3	21	21	22.1	23.7	21.5	21.7	21.2	20.5	21.8	21.4	21.4	20.7	21.6	21.8	21.1	22	24.9	21.3	21.1	27.7	21.7	22.9	22	22.8	21.5	22.7	21.5	27.3	22	21.6	21.7
N. mikamii	21.1	20.9	45.9	20.2	20.7	21.5	21.3	21.1	57.3	21.2	20.6	20.9	21	20.7	20.9	21.2	23.2	21.2	21	20.7	20.3	56.6	21.3	20.6	21	21	20.6	45.6	20.9	21.1	20.6	20.2	20.5	20.9	20.7	20.8	20.8	20.7	20.7	21.1	22.3	20.1	58	21	20.8	22.8	100	22.6	20.7	21.2	21.1	20.9	45.7	20.6	21.7	20.6	20.5	21.2	22.3	20.9	21.2	20.8	20.2	21.2	20.7	21	20.5	21.2	21.2	20.7	21.2	23	20.5	20.8	22.6	21.2	22.2	24.5	29.2	20.9	56.7	21.1	22.6	21.4	20.8	21.1
N. miyunensis	21.8	21.2	22.8	20.6	21.2	22	21.5	21.6	22.6	22	20.8	21.8	21.7	20.9	21.6	21.5	23.2	21.5	21.4	21	20.8	22.8	21.5	20.7	21.3	21.7	20.8	23.1	21.7	21.4	20.5	20.8	20.5	21.8	20.7	21	21.1	20.8	21.1	21.5	40.6	20.4	22.6	21.6	21.1	23.1	22.6	100	20.8	21.8	21.3	21.5	23.2	20.9	22.1	21	20.8	21.8	22.9	21.6	21.4	20.8	20.6	21.7	20.9	20.8	20.5	21.4	21.2	21.2	21.4	23.4	20.8	21	23.2	21.1	37.1	21.5	22.5	21.7	22.6	21.5	22.9	21.8	21.5	21.5
N. neocaledoniensis	22.1	20.6	20.8	26.8	21.7	22.3	22.6	21.4	21	21.9	21.5	22.1	22.1	31.7	22	22.8	21.3	22.1	21.4	27.3	20.7	20.8	20.8	27.4	20.8	22.2	21.3	21	22.2	22.1	21	27.3	20.9	22.1	21.1	21.6	21.8	27.5	20.8	21	20.8	20.6	20.8	22.5	28.8	21.1	20.7	20.8	100	21.1	22.1	21.9	20.9	23.8	21.5	20.9	20.9	22.4	20.8	21.6	22.4	21.2	27.1	21.2	21.8	21.2	20.4	20.9	22	21.5	22.4	21.4	21.5	23.9	21.1	20.9	20.9	20.3	20.7	21.6	20.9	22.1	21.2	22.5	20.8	21.2
N. niigatensis	21.4	23.3	21.3	20.6	20.8	21.6	21.6	21.2	21.5	21.4	20.8	21.4	21.4	21.2	21.2	21.7	21.9	21.4	21	21	20.4	21.5	30.2	20.8	23.9	21.3	20.6	21.4	21.3	21.4	20.3	20.6	20.5	21.6	20.4	20.8	21.2	20.8	24.1	24	21.5	20.2	21.4	21.6	21.1	21.9	21.2	21.8	21.1	100	21.4	21.3	21.4	21.1	23.3	20.8	20.3	21.6	21.5	21.1	21.4	20.6	20.5	30.4	21.2	20.4	19.9	21.5	21.3	20.9	21.4	22	20.7	21.1	21.8	22.5	21.5	20.7	21.4	21.3	21.3	21.4	21.8	21.8	24.3	29.5
N. ninae	24.9	21.1	21.2	21.6	27.7	25.1	23.7	23.6	21.3	24.8	22.2	24.5	24.7	22.3	24.5	23.8	21.7	27.6	22.2	21.9	21.3	21.4	21.1	21.8	21	22.8	22.1	21.5	24.7	22.8	21.4	21.6	21.8	24.6	21.4	22.1	22.6	21.9	21	21.4	21.4	21.2	21.2	23.8	22	21.9	21.1	21.3	22.1	21.4	100	24.6	21.4	21.8	21.7	21.7	21.4	25.3	20.9	23.7	22.9	21.8	21.4	21.3	22.4	21.7	21	21	59.7	22.2	31	22.1	21.8	21.9	21.6	21.5	21.3	20.6	21.3	23.7	21.2	27.6	21.8	23.8	21.1	20.9
N. niwae	36.2	20.9	21.1	21	23.6	31	23.7	22.9	21.5	38	22	38.6	35	22	36.9	23.6	21.8	24.2	22.6	21.6	21.4	21.4	21.1	21.4	21	22.7	22.3	21.2	39.1	22.9	21.3	21.2	21.5	39	21.8	22.2	22.5	21.4	20.9	21.2	21.5	21.5	21.2	23.9	21.6	21.7	20.9	21.5	21.9	21.3	24.6	100	21.3	21.8	21.5	21.8	21.6	31.2	21.4	23.1	23.1	21.9	21.2	21.2	22.5	21.8	21.2	20.8	24.5	22.3	24.7	22.1	22.1	21.9	21.7	20.9	21.4	20.6	21	23.1	21.2	24.1	21.7	23.8	21.1	21.2
N. nova	21.3	21.2	51	20.4	20.8	21.5	21.4	20.8	46.1	21.3	20.6	21.2	21.5	20.9	21.1	21.3	22.9	21.3	21.6	21	20.8	46.1	21.2	20.9	21.2	21.4	20.9	75.1	21.4	21.3	20.5	20.4	20.8	21.1	21.1	20.9	21.1	21	20.9	21.4	22.5	20.5	46.3	21.3	21.3	23	45.7	23.2	20.9	21.4	21.4	21.3	100	20.9	21.8	20.9	20.7	21.5	22.5	20.9	21.5	21.1	20.1	21.3	20.8	21	20.7	21	21.4	20.8	21.4	23.3	20.6	21	22.8	21.1	22.4	24.9	28.8	20.8	46	21.2	22.8	21.1	21.1	21.3
N. novocastrense	21.9	20.5	20.7	22.6	21.6	22	22.4	21.3	20.8	21.9	22.1	21.9	22	24.6	21.8	22.4	21.3	21.8	21.3	23.1	20.5	21	21	23	20.9	21.9	21	20.9	21.8	22	20.9	22.8	20.9	21.8	21	21.7	21.7	23.1	20.8	20.9	20.9	20.4	20.8	22.3	23.7	20.9	20.6	20.9	23.8	21.1	21.8	21.8	20.9	100	21.2	20.9	20.6	22	20.7	21.2	22.5	21.1	22.5	21.1	21.7	21	20.4	20.9	21.8	21.6	22.1	21.3	20.9	89	21	20.8	20.6	20.3	20.7	21.3	20.6	21.8	21.1	22.4	21.1	20.9
N. otitidiscaviarum	21.7	22.7	21.9	20.7	21	21.8	21.9	21.3	21.9	21.7	21.1	21.7	21.8	21.4	21.7	22	22.3	21.4	21.5	21	20.7	21.9	22.8	20.9	23.3	21.6	21	21.7	21.7	21.5	20.7	20.8	21	21.8	20.9	20.9	21.6	20.9	22.9	23.1	21.7	20.5	21.8	21.9	21.5	22.3	21.7	22.1	21.5	23.3	21.7	21.5	21.8	21.2	100	21.1	21	21.9	21.8	21.3	21.6	20.9	20.6	23.1	21.4	21.3	20.7	21.4	21.4	21.3	21.8	22.6	21.2	21.3	22.1	25.3	21.8	20.9	21.7	21.4	21.8	21.4	22.2	22	23.1	22.9
N. panacis	21.9	20.5	20.8	20.6	21.6	22.2	22.2	21.5	20.8	22	21.5	22	22.1	21.1	21.9	22.1	21.1	21.7	21.1	20.7	20.7	20.8	20.5	20.6	20.5	21.7	21	20.9	22	21.6	21	20.7	20.7	21.9	20.6	21	21.6	20.7	20.6	20.7	21	20.4	20.9	22.2	21	21	20.6	21	20.9	20.8	21.7	21.8	20.9	20.9	21.1	100	21	22.3	21	21.7	22	21.1	20.3	20.7	21.6	21.3	20.3	20.4	21.8	21.6	21.9	21.3	21	21	21.3	20.6	21	20	20.8	21.8	20.8	22	21.1	22.2	20.6	20.5
N. paucivorans	21.5	20.1	20.8	20.3	21.3	21.7	21.6	21.3	20.6	21.6	21	21.4	21.5	20.8	21.4	21.5	21.3	21.3	34.1	20.6	22.2	20.8	20.3	20.5	20.3	22	22.4	20.7	21.4	21.6	22.1	20.3	22	21.5	22.1	21.4	21.3	20.6	20.3	20.3	20.8	21.9	20.5	21.8	20.6	21	20.5	20.8	20.9	20.3	21.4	21.6	20.7	20.6	21	21	100	21.9	21	21.3	21.6	22	20.3	20.3	21.1	22	23.1	20.6	21.5	20.9	21.6	21.5	22.2	20.7	21.1	20.6	21.1	20.5	21.1	21.3	20.5	21.4	21	21.6	20.2	20.2
N. pneumoniae	32.1	21.2	21.4	21.6	24.3	46.1	24.4	23.5	21.6	31.7	22.6	31.6	31.8	22.3	31.1	24.5	22.2	24.9	22.7	22	21.5	21.7	21.4	22	21.3	23.1	22.6	21.4	31.7	23.3	21.6	21.5	21.7	31.6	21.7	22.6	23	21.9	21.1	21.4	21.5	21.5	21.5	24.6	22.2	22.1	21.2	21.8	22.4	21.6	25.3	31.2	21.5	22	21.9	22.3	21.9	100	21.5	23.6	23.5	21.8	21.3	21.5	22.7	21.8	21.4	21	25.2	22.6	25.4	22.3	21.8	22	21.9	21.3	21.5	20.7	21.5	23.5	21.5	24.9	22	24.6	21.2	21.2
N. pseudobrasiliensis	21.3	21.1	22.3	20.3	20.8	21.5	21.3	21.2	22.5	21.4	20.9	21.3	21.4	20.9	21.5	21.4	23.3	21.4	21.2	20.4	20.4	22.7	21.2	20.4	21.3	21.1	20.9	22.4	21.4	21.3	20.4	20.6	20.7	21.3	20.6	20.5	21.3	20.4	21.2	21.5	22.5	20.6	22.4	21.2	20.7	23.7	22.3	22.9	20.8	21.5	20.9	21.4	22.5	20.7	21.8	21	21	21.5	100	21	21.4	20.8	20.2	21.4	21	20.9	20.4	21.2	20.9	20.8	21.5	24	20.9	20.7	23.3	21.5	22.5	21.4	22.1	21.1	22.5	21.3	23.2	21.3	21.3	21.4
N. pseudovaccinii	23.1	20.8	20.9	21.1	23	23.8	23.9	43.6	21	23.2	22.1	23.1	23.2	21.5	23.1	23.6	21.4	23.1	21.9	21.6	20.9	20.9	20.8	21.5	20.8	22.4	21.7	20.9	23.1	22.3	21	21.1	21.4	23.2	21.4	21.5	21.9	21.4	20.8	20.9	21.4	21	21	23.5	21.4	21.5	20.9	21.6	21.6	21.1	23.7	23.1	20.9	21.2	21.3	21.7	21.3	23.6	21	100	22.2	21.5	21.2	21.1	21.8	21.6	20.9	20.5	23.5	21.9	23.5	21.6	21.6	21.3	21.3	21.1	21.3	20.5	21.1	53.1	21	23.2	21.3	23.4	20.8	21
N. puris	23.2	21.1	21.4	21.5	22.4	23.6	25.7	22.5	21.3	23.3	24.8	23.2	24.1	22.5	23.2	25.7	21.8	22.8	22.1	21.9	21.4	21.2	21.3	21.9	21.3	23.1	21.8	21.4	23.3	24.4	21.5	21.7	21.8	23.2	21.6	22.5	23.4	22.1	21.1	21.3	21.3	21.2	21.3	25.6	22	21.7	21.2	21.4	22.4	21.4	22.9	23.1	21.5	22.5	21.6	22	21.6	23.5	21.4	22.2	100	22	21.6	21.5	23.3	22.1	22.8	21	22.8	22.7	23.2	22	21.8	22.5	21.6	21.4	21.3	20.7	21	22.4	21.2	22.7	22.3	25.6	21.2	21.4
N. rhamnosiphila	21.7	20.4	21	20.5	21.4	22	21.7	21.5	20.8	21.6	21.1	21.8	21.7	21	21.7	21.9	21.3	21.5	23	20.8	25.8	21	20.7	20.9	20.7	21.9	22.1	21	21.9	21.5	25.8	21	55.5	21.6	47.4	21.4	21.4	21.1	20.4	20.4	20.6	33.1	20.8	21.8	21	21.2	20.8	20.8	21.2	20.6	21.8	21.9	21.1	21.1	20.9	21.1	22	21.8	20.8	21.5	22	100	20.5	20.7	21.4	47.5	25.3	20.7	21.3	21.2	21.5	21.5	43.6	21.2	21.3	21.1	20.6	20.6	21.1	21.4	20.7	21.5	21	21.9	20.4	20.3
N. salmonicida	21.2	20.3	20.6	38.5	21.1	21.3	21.6	21.1	20.5	21.4	20.8	21.2	21.3	27.6	21.1	21.5	20.6	21.2	20.6	26.1	20.2	20.5	20.4	26.6	20.1	21.5	20.5	20.3	21.2	21	20.2	41.8	20.3	21	20.3	20.5	21.1	26.8	20.4	20.6	20.2	20	20.3	21.4	29	20.5	20.2	20.6	27.1	20.5	21.4	21.2	20.1	22.5	20.6	20.3	20.3	21.3	20.2	21.2	21.6	20.5	100	20.6	21	20.8	20.1	20.6	21.4	20.6	21.5	20.7	20.4	22.4	20.3	20.7	20.3	19.9	20.5	21.1	20.3	21.3	20.3	21.6	20.4	20.5
N. seriolae	21.2	23.4	21.3	20.7	21	21.6	21.5	21	21.5	21.4	20.5	21.2	21.4	21.1	21.2	21.7	21.9	21.3	20.6	21	20.4	21.4	32.7	20.7	23.8	21.1	20.3	21.3	21.3	21.2	20.5	20.7	20.5	21.2	20.7	20.6	21.1	20.7	24.1	23.8	21.3	20.3	21.2	21.5	21.1	21.8	21.2	21.7	21.2	30.4	21.3	21.2	21.3	21.1	23.1	20.7	20.3	21.5	21.4	21.1	21.5	20.7	20.6	100	20.9	20.6	20.4	21.3	21.1	20.8	21.4	22	20.8	21.1	21.7	22.4	21.2	20.4	21.2	21	21.4	21.3	21.6	21.5	24.4	30.5
N. shimofusensis	22.3	20.6	20.9	21.1	21.9	22.7	23.8	22	20.8	22.7	21.8	22.4	22.6	21.9	22.5	23.6	21.5	22.4	21.8	21.6	21.4	21	21	21.5	20.8	22.3	21.3	20.8	22.4	25.9	21.2	21.2	21.4	22.4	21.5	21.9	36.2	21.5	20.9	21	21.1	21.3	20.9	23.8	21.6	21.4	20.7	20.9	21.8	21.2	22.4	22.5	20.8	21.7	21.4	21.6	21.1	22.7	21	21.8	23.3	21.4	21	20.9	100	21.4	21.1	20.7	22.2	22	22.5	21.8	22	21.7	21.3	21.3	20.9	20.3	21.1	21.8	20.7	22.3	21.2	23.7	20.9	21
N. sienata	21.9	20.5	20.8	20.6	21.6	21.8	21.8	21.8	21.1	21.9	21.1	22	21.7	21	21.8	22	21.1	21.6	23	21	25.6	21.2	21	20.8	20.5	22.2	22.6	20.9	21.9	21.8	25.5	20.9	47.6	21.8	44	21.3	21.5	21	20.3	20.5	20.8	32.8	21.2	22.1	21	21.4	21	20.8	21.2	20.4	21.7	21.8	21	21	21.3	21.3	22	21.8	20.9	21.6	22.1	47.5	20.8	20.6	21.4	100	24.9	20.5	21.5	21.2	21.8	21.5	42.7	21.1	21.3	21	20.7	20.7	21.1	21.4	20.9	21.5	21.3	22	20.6	20.6
N. speluncae	21.3	20.2	20.3	20	20.8	21.4	21.3	20.9	20.7	21.2	22.5	21.2	22.7	20.6	20.9	21.4	20.7	21.3	22.3	20.7	41.7	20.8	19.9	20.7	20.4	21.2	21.7	20.5	21.1	21	39.9	20.3	25.5	21	25.3	20.9	21	20.5	20	20.5	20.4	25	20.5	21.1	20.9	20.7	20.5	20.5	20.4	19.9	21	21.2	20.7	20.4	20.7	20.3	23.1	21.4	20.4	20.9	22.8	25.3	20.1	20.4	21.1	24.9	100	20	20.8	20.7	21.1	21.2	25.3	20.5	20.4	20.2	20.3	21.1	20.6	21	20.7	21.2	21.6	21.3	20.5	20
N. stercoris	21.1	20.9	21.3	20.6	20.7	21.2	21	20.6	21.2	21.2	20	20.8	21.3	21	21.1	21.2	21.8	21	20.9	20.9	20.4	21.3	21.4	20.9	20.9	21	20.2	21.2	20.7	20.8	20.5	20.5	20.6	20.7	20.6	20.6	20.7	20.9	20.9	21	21	19.8	21.2	21.4	20.9	21.6	21.2	21.4	20.9	21.5	21	20.8	21	20.9	21.4	20.4	20.6	21	21.2	20.5	21	20.7	20.6	21.3	20.7	20.5	20	100	20.8	20.4	20.9	22	20.7	21	21.5	21.2	21.4	20.8	21.1	20.5	21	20.9	21.5	21.3	21.2	21.2
N. suismassiliense	24.8	21	21.3	21.4	27.8	24.9	23.6	23.4	21.4	24.6	22	24.5	24.7	22.2	24.4	23.7	21.7	27.6	21.9	21.9	21.1	21.5	20.9	21.6	20.8	22.8	22.1	21.3	24.7	22.8	21.1	21.6	21.3	24.6	21.2	22	22.6	21.6	21	21.3	21.2	21.2	21.3	23.9	21.9	21.8	21.2	21.2	22	21.3	59.7	24.5	21.4	21.8	21.4	21.8	21.5	25.2	20.9	23.5	22.8	21.3	21.4	21.1	22.2	21.5	20.8	20.8	100	22.1	31	21.9	21.8	21.8	21.6	21.4	21.3	20.4	21.3	23.6	21.2	27.6	21.5	23.7	20.9	21
N. takedensis	22.3	20.2	20.6	20.9	21.7	22.6	23	21.9	20.8	22.4	26	22.3	22.4	21.6	22.4	23.2	21.2	22.2	21.3	21.1	21	21.1	20.7	21.1	20.6	22.1	21.4	20.7	22.4	22.8	21	21.1	20.9	22.4	20.9	21.5	22.2	21.1	20.4	20.9	20.8	20.5	20.6	23	21.5	21.1	20.7	21.2	21.5	20.9	22.2	22.3	20.8	21.6	21.3	21.6	20.9	22.6	20.8	21.9	22.7	21.2	20.6	20.8	22	21.2	20.7	20.4	22.1	100	22.4	21.4	21.1	21.6	21.1	20.7	20.9	20.1	20.6	22	20.7	22.2	21.3	22.9	20.7	20.8
N. tenerifensis	24.8	20.9	21.4	21.6	27.4	25.2	24.1	23.4	21.2	24.8	22.3	24.7	24.8	22.2	24.6	24	22	27.4	22.1	22	21.2	21.3	21.2	21.9	21.1	22.7	22.1	21.4	24.7	23	21.2	21.8	21.4	24.7	21.4	22.2	22.7	21.8	21.1	21.5	21.4	21	21.4	23.9	22.1	22	21.2	21.4	22.4	21.4	31	24.7	21.4	22.1	21.8	21.9	21.6	25.4	21.5	23.5	23.2	21.5	21.5	21.4	22.5	21.8	21.1	20.9	31	22.4	100	22.1	21.7	22.2	21.6	21.1	21.4	20.6	21.3	23.6	21.2	27.4	21.9	24	21.3	21.2
N. terpenica	22.1	21.4	23.2	20.6	21.6	22.5	22.2	21.6	23	22.3	21.2	22.1	22.2	21.4	22.3	22.3	24.9	21.8	21.8	21.3	20.9	23.2	21.8	21.1	21.7	22	21.3	23.2	22.1	21.9	21	21	21.4	22	21.3	21.4	21.7	21.1	21.6	22	23.1	20.9	23.2	22.2	21.3	24.9	23	23.4	21.4	22	22.1	22.1	23.3	21.3	22.6	21.3	21.5	22.3	24	21.6	22	21.5	20.7	22	21.8	21.5	21.2	22	21.9	21.4	22.1	100	21.4	21.5	24.4	21.9	23.2	22	23	21.8	22.9	21.8	24.7	22.2	21.8	22
N. testacea	21.8	20.5	20.7	20.8	21.3	22.1	21.9	21.4	20.8	21.8	21	21.8	21.8	21	21.6	22	21.1	21.8	23	20.9	25.8	20.8	21.1	20.8	20.8	22.2	22.1	20.8	21.8	21.6	25.8	20.9	43.6	21.7	42.1	21.5	21.5	20.9	20.3	20.8	20.4	32.5	20.7	22.1	21	21.3	20.5	20.8	21.5	20.7	21.8	22.1	20.6	20.9	21.2	21	22.2	21.8	20.9	21.6	21.8	43.6	20.4	20.8	22	42.7	25.3	20.7	21.8	21.1	21.7	21.4	100	21.3	21.4	21.1	20.9	21.1	20.9	21.6	20.6	21.9	21	22.1	20.4	20.7
N. thailandica	22	20.6	20.7	22.7	21.6	22	22.4	21.4	20.9	22	21.5	21.9	22.2	24.3	21.9	22.4	21.3	21.8	21.2	23.2	20.5	21	21	23.1	20.9	22	21	21	21.9	22.2	20.9	22.9	21	21.9	21.4	21.7	21.8	23.1	20.8	21	20.9	20.4	21	22.4	23.7	21.1	20.8	21	23.9	21.1	21.9	21.9	21	89	21.3	21	20.7	22	20.7	21.3	22.5	21.2	22.4	21.1	21.7	21.1	20.5	21	21.8	21.6	22.2	21.5	21.3	100	20.9	20.7	20.7	20.4	21	21.4	20.8	21.9	21.2	22.3	21.2	21
N. transvalensis	21.8	21.2	22.9	20.6	21.1	21.9	21.7	21.3	22.7	21.8	20.9	21.8	21.9	21.2	21.7	21.9	28.5	21.6	21.6	20.8	20.6	22.8	21.7	20.8	21.5	21.6	21	22.8	22	21.5	20.8	20.8	21	21.8	21.2	21.1	21.4	20.8	21.5	21.6	22.9	20.7	22.8	21.7	21	27.7	22.6	23.2	21.1	21.8	21.6	21.7	22.8	21	22.1	21.3	21.1	21.9	23.3	21.3	21.6	21.3	20.3	21.7	21.3	21.3	20.4	21.5	21.6	21.1	21.6	24.4	21.4	20.9	100	21.4	23	21.7	22.6	21.3	22.5	21.5	27.8	21.7	21.6	21.7
N. uniformis	21.3	22.2	21.2	20.7	20.7	21.6	21.3	21	21.3	20.9	20.4	20.6	21.1	20.9	21.2	21.5	21.4	21.1	21.2	20.7	20.5	21.3	22.3	20.8	22.7	21	20.5	21	20.9	20.8	20.7	20.8	20.8	21	20.9	20.4	21.1	20.7	22.3	22.5	21.3	20.6	21.3	21.6	21.4	21.7	21.2	21.1	20.9	22.5	21.5	20.9	21.1	20.8	25.3	20.6	20.6	21.3	21.5	21.1	21.4	21.1	20.7	22.4	21.3	21	20.2	21.2	21.4	20.7	21.1	21.9	21.1	20.7	21.4	100	21.1	20.5	21.3	21.1	21.2	21	21.4	21.5	22.5	22.2
N. vaccinii	21.4	21.2	22.2	20.6	21.1	21.8	21.5	21.2	22.2	21.2	20.9	21.8	21.3	20.8	21.4	21.5	22.7	21.4	21.3	20.7	20.4	22.4	21.2	20.6	21.1	21.3	20.8	22.4	21.6	21.1	20.6	20.5	20.3	21.4	20.7	21	21.2	20.6	21.1	21.4	38.2	20.2	22.2	21.6	20.7	22.9	22.2	37.1	20.9	21.5	21.3	21.4	22.4	20.6	21.8	21	21.1	21.5	22.5	21.3	21.3	20.6	20.3	21.2	20.9	20.7	20.3	21.4	21.3	20.9	21.4	23.2	20.9	20.7	23	21.1	100	21.3	22.2	21.5	22.2	21.3	22.7	21.7	21.3	21.4
N. vermiculata	20.6	20.5	24.7	20.1	20.2	20.7	20.6	20.5	24.9	20.7	20.1	20.5	20.5	20.5	20.6	20.9	21.7	20.6	20.7	20.4	20.7	25	20.7	20.3	20.5	20.7	20.4	24.9	20.6	20.4	20.9	20.4	20.6	20.5	20.9	20.2	20.3	20.5	20.6	20.7	21.3	20.1	24.9	20.7	20.6	22	24.5	21.5	20.3	20.7	20.6	20.6	24.9	20.3	20.9	20	20.5	20.7	21.4	20.5	20.7	20.6	19.9	20.4	20.3	20.7	21.1	20.8	20.4	20.1	20.6	22	21.1	20.4	21.7	20.5	21.3	100	24.5	20.5	24.8	20.5	21.6	20.9	20.5	20.4
N. veterana	21.4	20.9	29.4	20.2	20.7	21.7	21.1	20.8	29.1	21.5	20.5	21.3	21.5	21	21.2	21.3	22.9	21.2	21	20.8	20.9	29.2	21.3	20.9	20.9	21.1	20.6	28.8	21.4	21.4	20.7	20.7	20.6	21.3	21	20.9	21.1	21	21	21.1	22.1	20.6	28.9	21.2	21	22.8	29.2	22.5	20.7	21.4	21.3	21	28.8	20.7	21.7	20.8	21.1	21.5	22.1	21.1	21	21.1	20.5	21.2	21.1	21.1	20.6	21.1	21.3	20.6	21.3	23	20.9	21	22.6	21.3	22.2	24.5	100	21	28.9	21.1	22.6	21.3	21.3	21.4
N. vinacea	23.1	20.9	21	21	23.1	24.1	23.8	43.6	21	23.2	22.1	23.1	23.4	21.7	23	23.6	21.2	23.3	22	21.6	21	21.2	21.1	21.6	20.8	22.4	21.8	20.9	23.2	22.2	21.2	21.4	21.6	23	21.4	21.6	21.8	21.6	20.8	21.1	21.4	21	21.1	23.7	21.7	21.5	20.9	21.7	21.6	21.3	23.7	23.1	20.8	21.3	21.4	21.8	21.3	23.5	21.1	53.1	22.4	21.4	21.1	21	21.8	21.4	21	20.5	23.6	22	23.6	21.8	21.6	21.4	21.3	21.1	21.5	20.5	21	100	21.6	23.3	21.3	23.5	20.8	21.1
N. violaceofusca	21.1	21	47.2	20.2	20.7	21.9	21.4	21	93.8	21.4	20.8	21.3	21.3	20.8	21.1	21.3	22.9	21	21	20.7	20.4	61.3	21.3	20.8	21.1	21.3	20.8	46.1	21.3	21.2	20.7	20.5	20.7	21.3	20.7	20.9	20.9	20.9	20.9	21	22.2	20.5	64.2	21.1	21	22.7	56.7	22.6	20.9	21.3	21.2	21.2	46	20.6	21.8	20.8	20.5	21.5	22.5	21	21.2	20.7	20.3	21.4	20.7	20.9	20.7	21	21.2	20.7	21.2	22.9	20.6	20.8	22.5	21.2	22.2	24.8	28.9	21.6	100	21.1	22.6	21.1	21.3	21.3
N. vulneris	24.4	21.1	21	21.4	26.6	24.7	23.6	23.3	21.2	24.3	22.3	24	24.5	22	24.1	23.6	21.6	65.6	22.2	21.8	21.1	21.3	21	21.8	21	22.7	21.9	21.3	24.3	22.7	21.2	21.4	21.5	24.1	21.4	22.2	22.3	21.7	21	21.2	21.3	21.1	21.2	23.5	22	21.5	21.1	21.5	22.1	21.4	27.6	24.1	21.2	21.8	21.4	22	21.4	24.9	21.3	23.2	22.7	21.5	21.3	21.3	22.3	21.5	21.2	20.9	27.6	22.2	27.4	21.8	21.9	21.9	21.5	21	21.3	20.5	21.1	23.3	21.1	100	21.4	23.6	21	21.1
N. wallacei	22.1	21.2	22.7	20.5	21.1	22	21.9	21.4	22.8	21.9	21.6	21.8	22.4	21.3	21.9	21.8	31.8	21.4	21.4	20.8	20.8	22.9	21.6	20.8	21.4	21.6	20.8	22.8	22	21.6	20.9	20.6	21	21.7	20.9	21.3	21.4	20.7	21.4	21.3	22.7	20.6	22.6	21.9	21.1	27.3	22.6	22.9	21.2	21.8	21.8	21.7	22.8	21.1	22.2	21.1	21	22	23.2	21.3	22.3	21	20.3	21.6	21.2	21.3	21.6	21.5	21.5	21.3	21.9	24.7	21	21.2	27.8	21.4	22.7	21.6	22.6	21.3	22.6	21.4	100	21.8	21.6	21.8
N. xishanensis	24	21.3	21.3	21.6	23.2	24.6	36.1	23.3	21.3	24	23	24	23.9	22.4	24.1	36.5	22.1	23.4	22.2	21.9	21.3	21.3	21.4	21.8	21.3	23.1	22.2	21	24	24.5	21.6	21.8	21.7	24	22	22.5	23.7	21.7	21.1	21.4	21.7	21.4	21.3	40.1	22.2	22	21.4	21.8	22.5	21.8	23.8	23.8	21.1	22.4	22	22.2	21.6	24.6	21.3	23.4	25.6	21.9	21.6	21.5	23.7	22	21.3	21.3	23.7	22.9	24	22.2	22.1	22.3	21.7	21.5	21.7	20.9	21.3	23.5	21.1	23.6	21.8	100	21.2	21.3
N. yamanashiensis	21.2	23.4	21.1	20.5	20.9	21.4	21.5	20.8	21.1	21.4	20.5	21.1	21.3	20.9	21.3	21.4	21.7	21	20.7	20.9	20.5	21.2	24.3	20.7	24	21.1	20.3	21.1	21.1	21.2	20.5	20.6	20.4	21.1	20.2	20.7	21.1	20.7	34.7	23.8	21.3	20.1	21.1	21.4	21.1	21.6	20.8	21.5	20.8	24.3	21.1	21.1	21.1	21.1	23.1	20.6	20.2	21.2	21.3	20.8	21.2	20.4	20.4	24.4	20.9	20.6	20.5	21.2	20.9	20.7	21.3	21.8	20.4	21.2	21.6	22.5	21.3	20.5	21.3	20.8	21.3	21	21.6	21.2	100	24
N. yunnanensis	21.1	23.1	21.1	20.6	20.6	21.5	21.5	21.1	21.5	21.3	20.5	21.1	21.2	20.9	21.2	21.5	21.8	21.1	20.7	20.7	20.3	21.5	30.3	20.8	23.5	21.1	20.3	21.2	21.1	21.2	20.3	20.7	20.4	21.2	20.4	20.5	21.1	20.9	23.7	23.7	21.3	19.9	21.3	21.5	21.1	21.7	21.1	21.5	21.2	29.5	20.9	21.2	21.3	20.9	22.9	20.5	20.2	21.2	21.4	21	21.4	20.3	20.5	30.5	21	20.6	20	21.2	21	20.8	21.2	22	20.7	21	21.7	22.2	21.4	20.4	21.4	21.1	21.3	21.1	21.8	21.3	24	100
