# Supplementary material for: Whole genome sequencing reveals the genomic diversity, taxonomic classification, and evolutionary relationships of the genus Nocardia
Source: PLoS Negl Trop Dis. 2021 Aug 26;15(8):e0009665. doi: 10.1371/journal.pntd.0009665 (PMC8437295; doi:10.1371/journal.pntd.0009665)
Supplement: S6 Table — (PDF) [file pntd.0009665.s006.pdf]

S6 Table. Reclassification of genomes that are currently unclassified *Nocardia* species**.**

Phylogroup	NCBI species	closest related type strain	ANI value (%)	*is* DDH (%)	Species
N. farcinica group	*Nocardia* sp. FDAARGOS_372	*N. farcinica* DSM43665[T]	99.15	92.4	*N. farcinica*
N. farcinica group	*Nocardia* sp. SYSU K10002	*N. takedensis* DSM44801[T]	80.25	23.3	putative new species 1
N. farcinica group	*Nocardia* sp. W9405	*N. arizonensis* DSM45748[T]	99.96	99.8	*N. arizonensis*
N. farcinica group	*Nocardia* sp. CNY236	*N. amamiensis* DSM45066[T]	80.48	24.2	putative new species 2
N. farcinica group	*Nocardia* sp. CICC 11023	*N. tenerifensis* DSM44704[T]	86.45	31.8	putative new species 3
N. farcinica group	*Nocardia* sp. CS682	*N. ninae* DSM44978[T]	99.96	63.3	*N. ninae*
N. asteroides group	*Nocardia* sp. Root136	*N. salmonicida* DSM40472[T]	97.66	79.5	*N. salmonicida*
N. transvalensis group	*Nocardia* sp. BMG111209	*N. stercoris* NEAU LL90[T]	77.02	21.3	putative new species 4
N. transvalensis group	*Nocardia* sp. RB56	*N. stercoris* NEAU LL90[T]	77.11	21.5	putative new species 5
N. transvalensis group	*Nocardia* sp. BMG51109	*N. blacklockiae* DSM 45135[T]	83.56	27.7	putative new species 6
N. transvalensis group	*Nocardia* sp. RB20	*N. vaccinii* DSM 43285[T]	86.53	32.4	putative new species 7
N. transvalensis group	*Nocardia* sp. NRRL WC3656	*N. nova* DSM44481[T]	97.78	81	*N. nova*
N. transvalensis group	*Nocardia sp.* 852002-20019_SCH5090214	*N. nova* DSM44481[T]	99.13	92.3	*N. nova*
N. transvalensis group	*Nocardia sp.* 852002-51101_SCH5132738	*N. nova* DSM44481[T]	99.26	93.9	*N. nova*
N. transvalensis group	*Nocardia sp.* 852002-51244_SCH5132740	*N. nova* DSM44481[T]	99.21	93.5	*N. nova*
N. transvalensis group	*Nocardia* sp. MDA0666	*N. aobensis* DSM44805[T]	97.06	75.8	*N. aobensis*
N. otitidiscaviarum group	*Nocardia* sp. SYP-A9097	*N. acidivorans* DSM 45049[T]	87.76	34.7	putative new species 8
N. otitidiscaviarum group	*Nocardia* sp. CT2-14	*N. niigatensis* DSM 44670[T]	87.98	35.2	putative new species 9
N. otitidiscaviarum group	*Nocardia* sp. ET3-3	*N. concava* DSM 44804[T]	86.55	32.3	putative new species 10
outgroup	*Nocardia* sp. 348MFTsu5.1	*Williamsia faeni* DSM 45372[T]	97.6	77.9	*Williamsia faeni*
outgroup	*Nocardia* sp. NRRL S-836	*Lechevalieria* spp	NA	NA	*Lechevalieria* spp
