# Supplementary material for: Whole genome sequencing reveals the genomic diversity, taxonomic classification, and evolutionary relationships of the genus Nocardia
Source: PLoS Negl Trop Dis. 2021 Aug 26;15(8):e0009665. doi: 10.1371/journal.pntd.0009665 (PMC8437295; doi:10.1371/journal.pntd.0009665)
Supplement: S7 Table — (PDF) [file pntd.0009665.s007.pdf]

S7 Table. General features of sequenced *Nocardia* species.

*Nocardia**	Strain ID	GSA ID	Isolation year	Source	Genome Size (Mb)	No. of CDSs	No. of scaffolds	%GC	No. of tRNAs	No. of rRNAs	No. of tmRNAs
abscessus	DSM 44557	CDC 71	2001	NA	8.08	7366	95	68.47	65	5	1
	isolate	CDC 167	2015	sputum	7.98	7209	69	68.37	70	6	1
africana	DSM 44500	CDC 73	2000	sputum	7.73	7083	32	67.93	55	3	1
amikacinitolerans	DSM 45538	CDC 100	2009	sputum	7.63	6718	35	68.6	68	4	2
asiatica	DSM 44700	CDC 104	2003	human	8.19	7430	46	68.82	69	6	1
	isolate	CDC193	2015	sputum	8.92	8219	77	68.39	65	6	1
	isolate	CDC194	2016	brain	7.66	6980	66	68.71	58	6	1
asteroides	DSM 43258	CDC 2	1990	soil	7.57	6898	24	68.46	65	7	1
beijingensis	isolate	CDC186	2015	sputum	7.20	6574	40	69.13	62	6	1
	isolate	CDC188	2015	sputum	7.44	6875	101	68.04	67	6	1
blacklockiae	DSM 45135^{T}	CDC 112	2007	human muscle abscess	8.29	7361	33	69.95	68	5	1
	isolate	CDC190	2015	sputum	8.10	7267	34	69.82	69	4	1
brasiliensis	DSM 43009	CDC126	1990	NA	9.58	8644	33	68.23	64	6	1
	DSM 46032	CDC128	1978	NA	9.09	8198	29	68.27	72	5	1
	DSM 46059	CDC129	1979	NA	9.30	8385	49	68.12	65	5	1
	isolate	CDC144	2015	sputum	9.39	8630	35	67.94	70	6	1
	isolate	CDC163	2015	pus from empysema	9.91	8913	41	67.88	74	5	1
	isolate	CDC196	2016	abscess	9.39	8584	19	68.1	72	6	1
carnea	DSM 44558	CDC122	2001	NA	7.88	7144	40	66.99	63	5	1
	DSM 44582	CDC123	2001	mineralic building material	7.97	7219	44	66.98	63	5	1
	DSM 46055	CDC124	NA	NA	7.60	6973	33	67.09	57	5	1
	DSM 46071	CDC125	1996	NA	8.17	7451	27	67.05	65	4	1
cyriacigeorgica	DSM 40350	CDC 133	1975	septicemic actinomycosis of dog	6.10	5524	21	68.48	60	5	1
	DSM 43004	CDC 134	1990	NA	6.54	6149	24	68.38	70	5	1
	DSM 43005	CDC 135	1990	NA	6.54	6144	25	68.38	70	5	1
	DSM 43208	CDC 136	1990	pus from empysema	6.65	5934	31	68.26	60	6	1
	DSM 46058	CDC 137	1979	NA	6.36	5865	54	68.4	61	5	1
	isolate	CDC 140	2015	sputum	6.20	5569	12	68.38	61	5	1
	isolate	CDC156	2015	sputum	6.68	6064	34	68.29	61	5	1
	isolate	CDC 182	2015	sputum	6.48	5826	11	68.38	63	5	1
	isolate	CDC 197	2016	lung	6.49	5863	12	68.35	62	2	1
	isolate	CDC 322	2016	pancreatic secretion	6.54	5857	28	68.26	60	5	1
	isolate	CDC 323	2016	special materials	6.42	5823	19	68.37	61	6	1
	isolate	CDC 327	2016	sputum	6.23	5616	19	68.46	59	4	2
	isolate	CDC 332	2016	alveolar lavage fluid	6.21	5647	26	68.46	60	5	1
	DSM 46028	CDC 98	1975	bovine farcy	6.07	5640	37	70.93	57	3	1
	isolate	CDC 25	2006	sputum	6.04	5607	23	70.92	57	3	1
	isolate	CDC 27	2011	sewer rate	6.26	5849	81	70.62	61	6	1
	isolate	CDC 30	2011	sewer rate	6.30	5888	77	70.61	61	6	1
	isolate	CDC 31	2011	sewer rate	6.26	5837	74	70.62	61	5	1
	isolate	CDC 32	2011	mole	6.26	5848	79	70.62	61	6	1
	isolate	CDC 35	2011	sputum	6.30	5886	78	70.61	61	6	1

farcinica	isolate	CDC 38	2011	sputum	6.30	5884	77	70.61	61	5	1
	isolate	CDC 42	2011	sputum	6.11	5705	39	70.89	56	6	1
	isolate	CDC 46	2010	sputum	6.39	5929	36	70.72	56	5	1
	isolate	CDC 48	2011	sputum	6.59	6093	51	70.77	57	4	1
	isolate	CDC 53	2010	sputum	6.39	5934	41	70.72	56	6	1
	isolate	CDC 56	2010	sputum	6.33	5854	50	70.76	56	5	1
	isolate	CDC 59	2010	sputum	6.39	5929	40	70.72	56	5	1
	isolate	CDC 65	2010	sputum	6.14	5771	40	70.86	62	3	1
	isolate	CDC 142	2015	sputum	6.11	5693	25	70.72	60	6	1
inohanensis	isolate	CDC 153	2015	eye secretion	8.22	7589	15	67.93	68	6	1
nova	DSM 43207	CDC 16	1990	foot sinus	7.86	7081	39	67.86	58	3	1
	DSM 40806	CDC 119	1993	NA	7.85	7141	30	67.79	63	6	1
	DSM 43209	CDC 120	1990	foot sinus	7.87	7090	43	67.86	58	4	1
	DSM 44559	CDC 121	2001	NA	7.81	7061	27	67.94	60	6	1
	isolate	CDC 141	2014	sputum	7.86	7376	50	68.57	59	5	1
	isolate	CDC 159	2015	sputum	7.87	7381	51	68.57	59	5	1
novocastrense	DSM 44692[T]	CDC 110	2003	bronchial lavage of a lung transplant patient	6.69	6308	60	71.66	75	4	1
otitidiscaviarum	DSM 44565	CDC 21	2001	sputum	7.33	6653	33	69.04	87	5	1
	DSM 43010	CDC 116	1990	NA	7.36	6729	43	68.99	89	6	2
	DSM 43398	CDC 117	1993	NA	7.51	6766	27	69.03	82	5	1
	isolate	CDC155	2015	sputum	7.25	6596	36	69.08	82	6	1
	isolate	CDC157	2015	liver puncture	7.61	6922	35	68.97	79	4	1
	isolate	CDC195	2016	lung	7.30	6645	42	69.03	88	6	1
	isolate	CDC324	2016	dialysate	7.66	6938	34	68.97	81	5	1
	isolate	CDC326	2016	sputum	7.57	6912	41	69.04	82	6	2
transvalensis	DSM 46068	CDC 115	1979	foot	8.72	8075	13	69.23	65	5	1
vaccinii	isolate	CDC 160	2015	sputum	8.57	7787	19	67.45	67	6	1
veterana	isolate	CDC 181	2015	alveolar lavage fluid	6.54	5890	74	68.29	59	3	1
wallacei	DSM 45136[T]	CDC 113	2007	sputum	8.04	7327	65	69.12	66	6	1
	DSM 45846	CDC 114	2013	human lungs- broncholaveolar lavage	7.29	6479	37	69.48	69	6	1

*16S rRNA identification result

[T] type strains
