# Supplementary figures and images for: Whole genome sequencing reveals the genomic diversity, taxonomic classification, and evolutionary relationships of the genus Nocardia
Source: PLoS Negl Trop Dis. 2021 Aug 26;15(8):e0009665. doi: 10.1371/journal.pntd.0009665 (PMC8437295; doi:10.1371/journal.pntd.0009665)

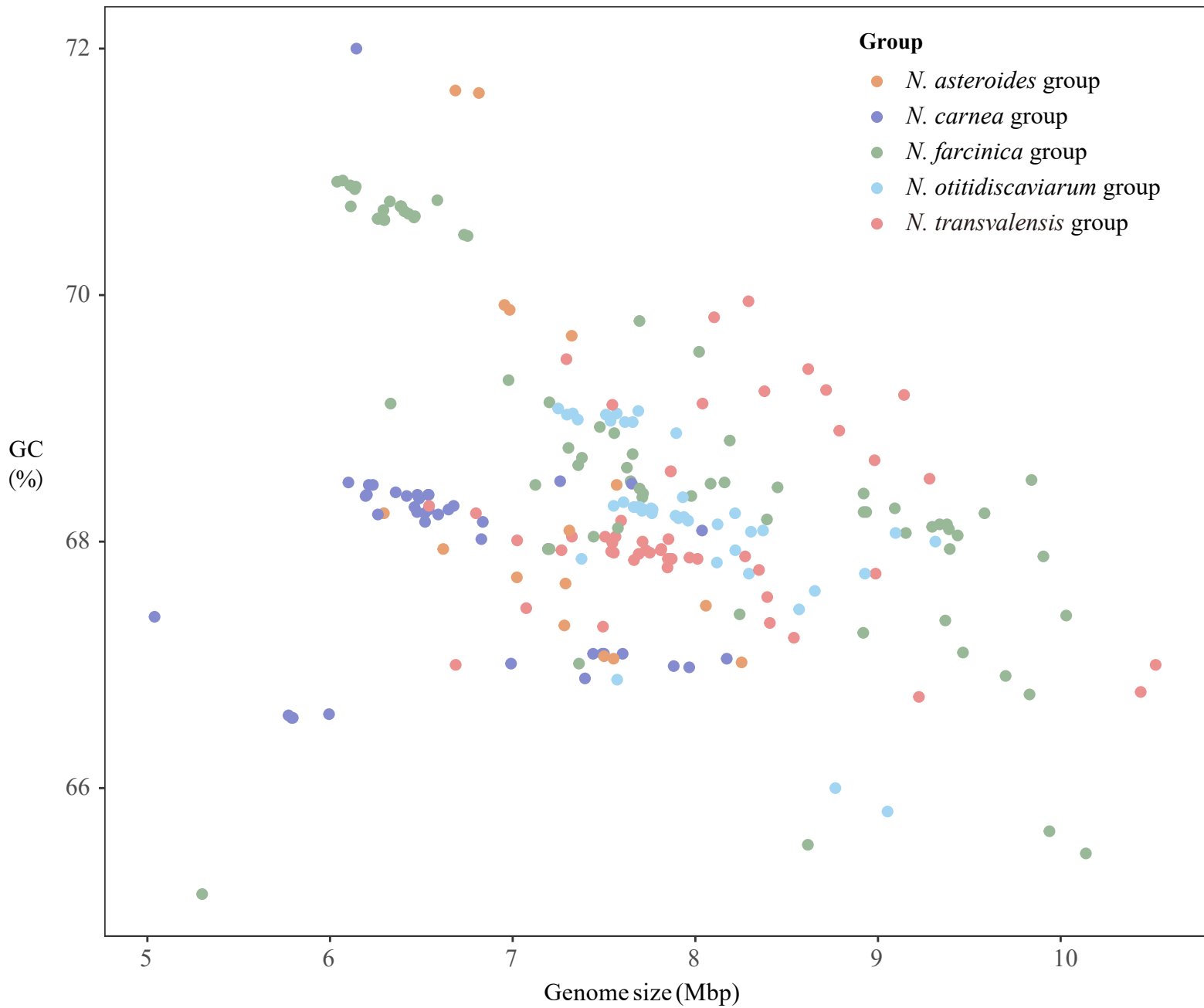

Supplement: S1 Fig — Different colors indicated taxonomic grouping as described in Fig 2. (PDF) [file pntd.0009665.s008.pdf]

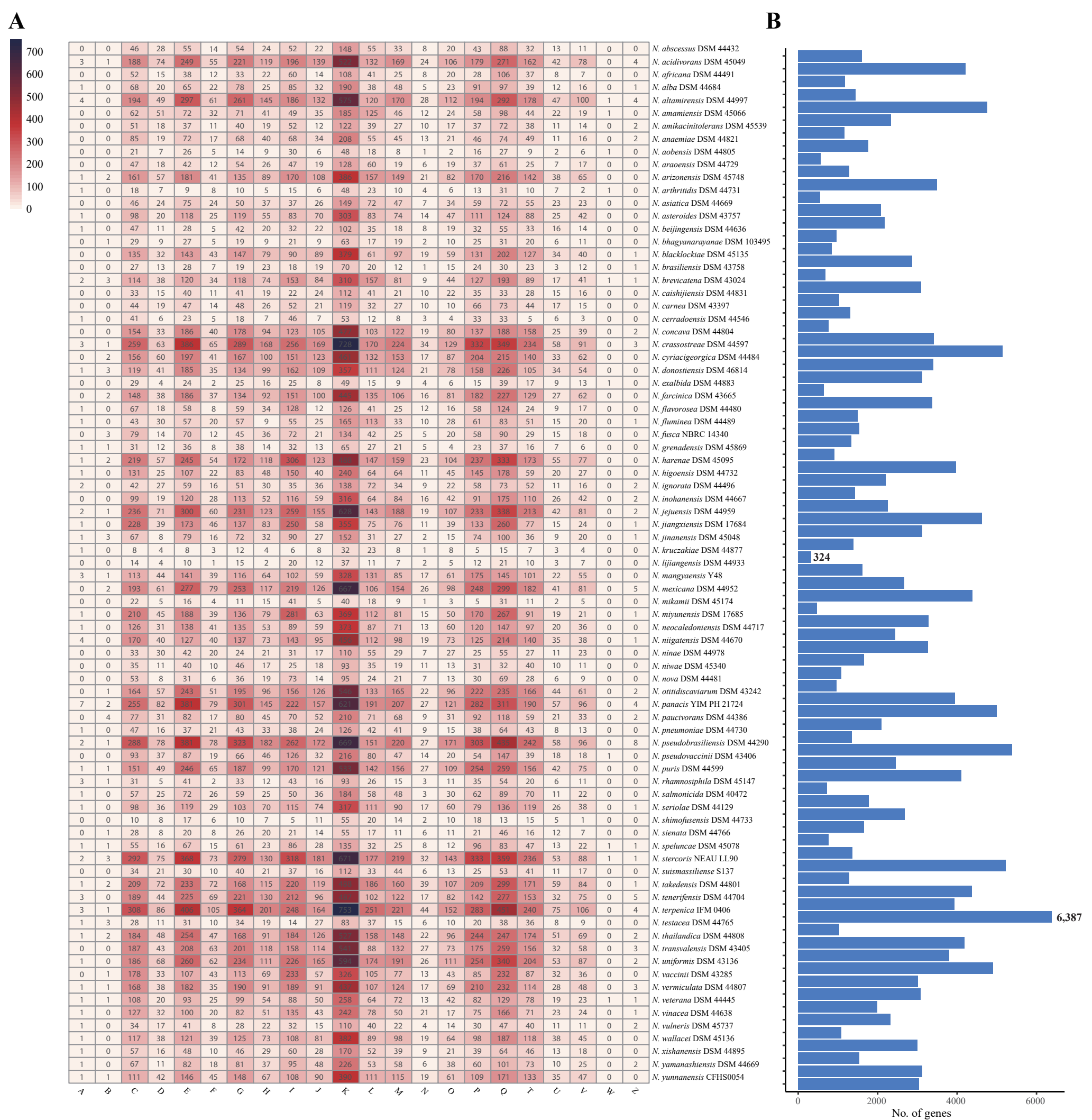

Supplement: S2 Fig — Genes with “Function unknown” were not included. (PDF) [file pntd.0009665.s009.pdf]

A

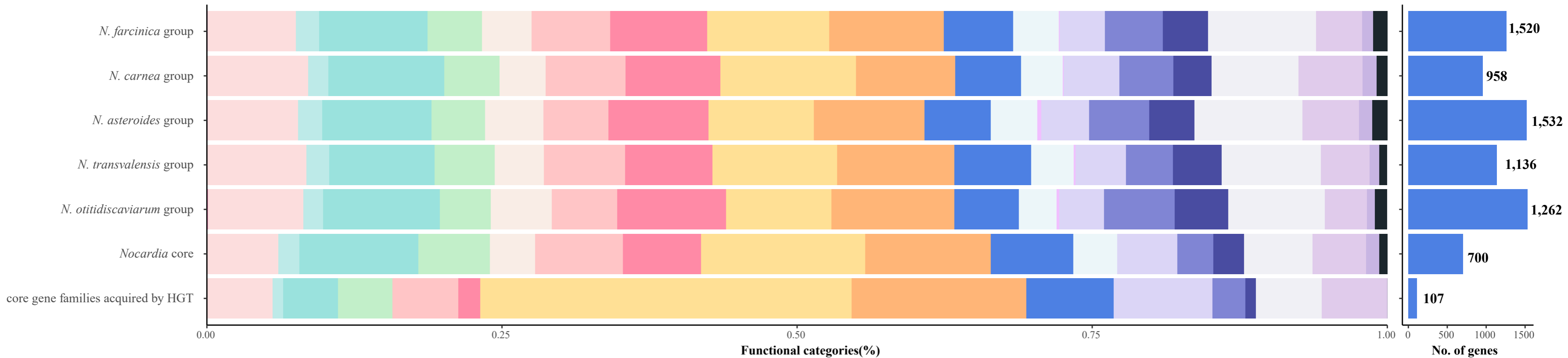

B

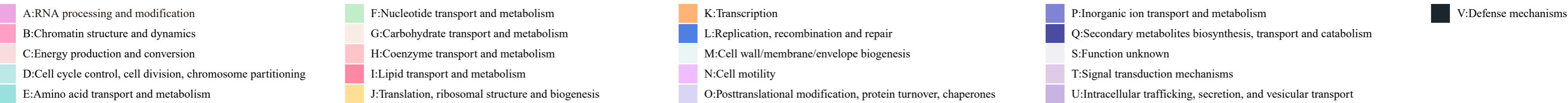

Supplement: S3 Fig — (A) Functional categories for core gene families in each phylogroup. (B) The number of core gene families in each phylogroup. (PDF) [file pntd.0009665.s010.pdf]

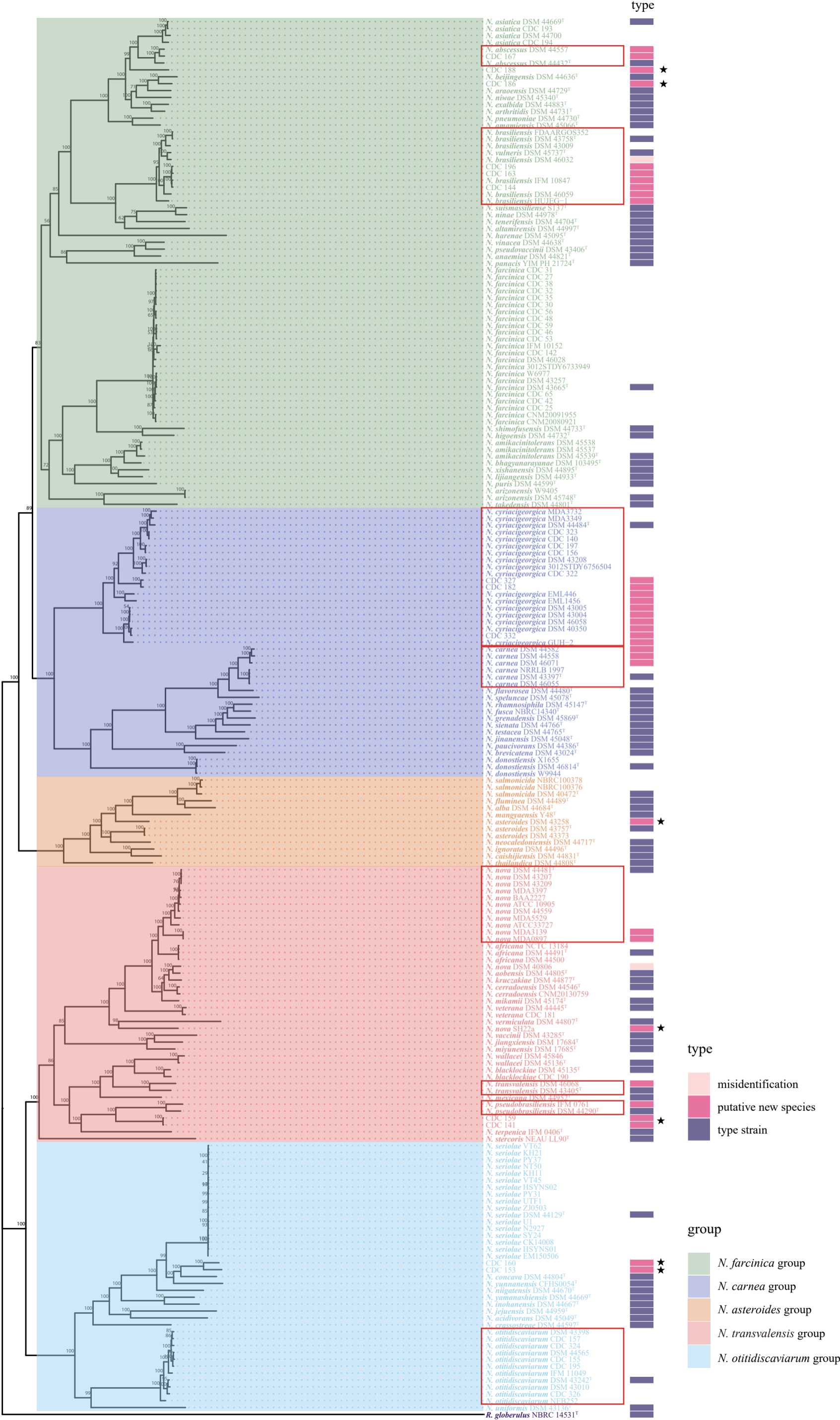

Supplement: S4 Fig — A maximum likelihood phylogenetic tree was constructed based on the concatenation of 241 single-copy genes of 81 type strains and 122 additional genomes of Nocardia spp. with 1000 bootstrap replicates using Rhodococcus globerulus NBRC 14531 as an outgroup. Bootstrap values are indicated on the nodes. Phylogenetic groups are highlighted in different colors. Red boxes indicate the same species has a type strain in different subgroup. The asterisk represents clusters lacking a type strain. (PDF) [file pntd.0009665.s011.pdf]
