# Supplementary material for: Whole genome sequencing reveals the genomic diversity, taxonomic classification, and evolutionary relationships of the genus Nocardia
Source: PLoS Negl Trop Dis. 2021 Aug 26;15(8):e0009665. doi: 10.1371/journal.pntd.0009665 (PMC8437295; doi:10.1371/journal.pntd.0009665)
Supplement: S5 Fig — Phylogenetic tree was constructed by the maximum likelihood method based on the five concatenated gene sequences (gyrB, 16S, secA1, hsp65, and rpoB) of 81 Nocardia type strains using Rhodococcus globerulus NBRC 14531 as an outgroup. Bootstrapping was carried out using 1000 replicates and values are shown at the nodes. (PDF) [file pntd.0009665.s012.PDF]

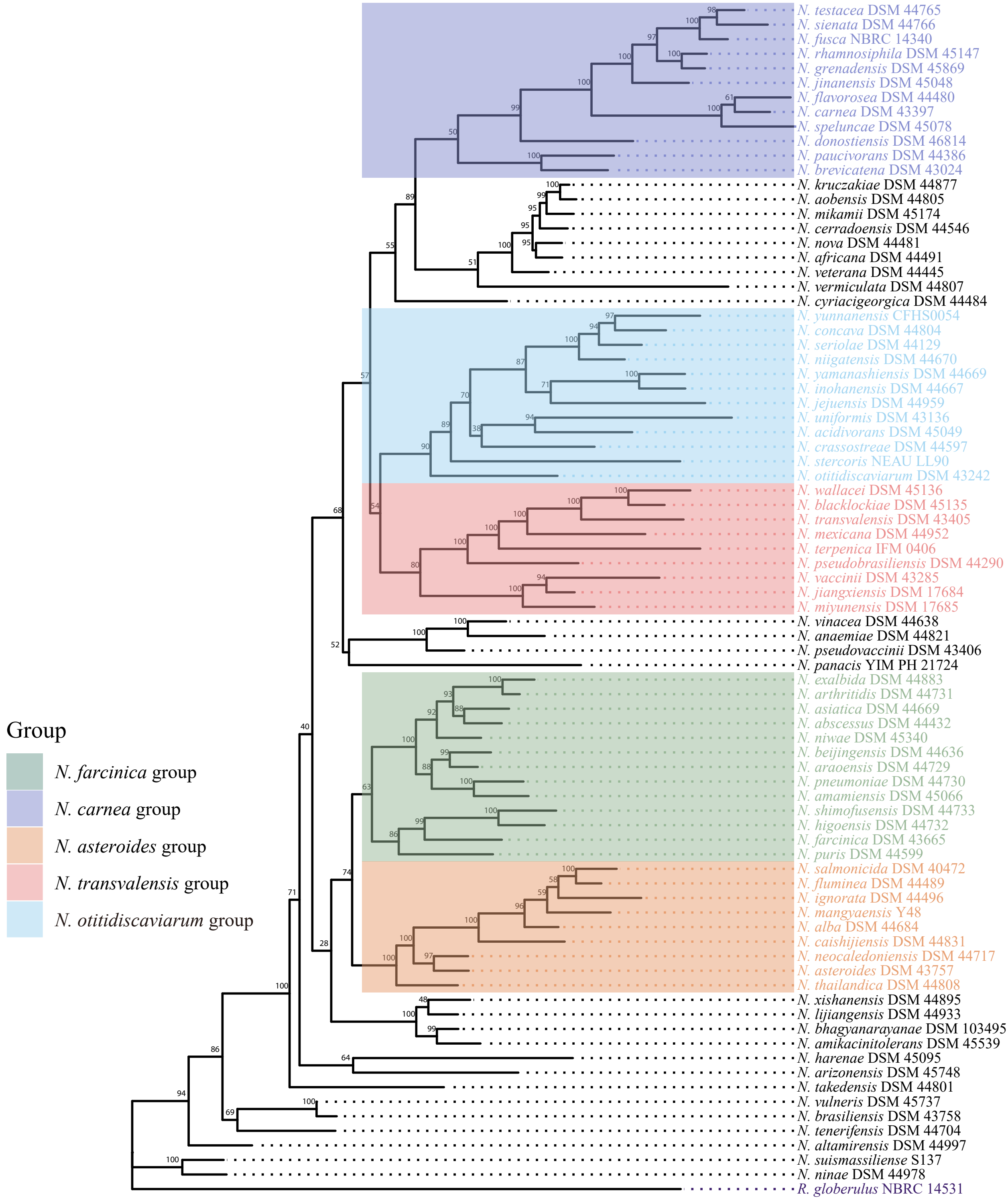
N. testacea DSM 44765
N. sienata DSM 44766
N. fusca NBRC 14340
N. rhamnosiphila DSM 45147
N. grenadensis DSM 45869
N. jinanensis DSM 45048
N. flavorosea DSM 44480
N. carnea DSM 43397
N. speluncae DSM 45078
N. donostiensis DSM 46814
N. paucivorans DSM 44386
N. brevicatena DSM 43024
N. kruczakiae DSM 44877
N. aobensis DSM 44805
N. mikamii DSM 45174
N. cerradoensis DSM 44546
N. nova DSM 44481
N. africana DSM 44491
N. veterana DSM 44445
N. vermiculata DSM 44807
N. cyriacigeorgica DSM 44484
N. yunnanensis CFHS0054
N. concava DSM 44804
N. seriolae DSM 44129
N. niigatensis DSM 44670
N. yamanashiensis DSM 44669
N. inohanensis DSM 44667
N. jejuensis DSM 44959
N. uniformis DSM 43136
N. acidivorans DSM 45049
N. crassostreae DSM 44597
N. stercoris NEAU LL90
N. otitidiscaviarum DSM 43242
N. wallacei DSM 45136
N. blacklockiae DSM 45135
N. transvalensis DSM 43405
N. mexicana DSM 44952
N. terpenica IFM 0406
N. pseudobrasiliensis DSM 44290
N. vaccinii DSM 43285
N. jiangxiensis DSM 17684
N. miyunensis DSM 17685
N. vinacea DSM 44638
N. anaemiae DSM 44821
N. pseudovaccinii DSM 43406
N. panacis YIM PH 21724
N. exalbida DSM 44883
N. arthritidis DSM 44731
N. asiatica DSM 44669
N. abscessus DSM 44432
N. niwae DSM 45340
N. beijingensis DSM 44636
N. araoensis DSM 44729
N. pneumoniae DSM 44730
N. amamiensis DSM 45066
N. shimofusensis DSM 44733
N. higoensis DSM 44732
N. farcinica DSM 43665
N. puris DSM 44599
N. salmonicida DSM 40472
N. fluminea DSM 44489
N. ignorata DSM 44496
N. mangyaensis Y48
N. alba DSM 44684
N. caishijiensis DSM 44831
N. neocaledoniensis DSM 44717
N. asteroides DSM 43757
N. thailandica DSM 44808
N. xishanensis DSM 44895
N. lijiangensis DSM 44933
N. bhagyanarayanae DSM 103495
N. amikacinitolerans DSM 45539
N. harenae DSM 45095
N. arizonensis DSM 45748
N. takedensis DSM 44801
N. vulneris DSM 45737
N. brasiliensis DSM 43758
N. tenerifensis DSM 44704
N. altamirensis DSM 44997
N. suismassiliense S137
N. ninae DSM 44978
R. globerulus NBRC 14531
Group
N. farcinica group
N. carnea group
N. asteroides group
N. transvalensis group
N. otitidiscaviarum group
